# A comprehensive simulation study on classification of RNA-Seq data

**DOI:** 10.1371/journal.pone.0182507

**Published:** 2017-08-23

**Authors:** Gökmen Zararsız, Dincer Goksuluk, Selcuk Korkmaz, Vahap Eldem, Gozde Erturk Zararsiz, Izzet Parug Duru, Ahmet Ozturk

**Affiliations:** 1 Turcosa Analytics Solutions Ltd Co, Erciyes Teknopark, 38039, Kayseri, Turkey; 2 Department of Biostatistics, Erciyes University, Kayseri, Turkey; 3 Department of Biostatistics, Hacettepe University, Ankara, Turkey; 4 Department of Biology, Istanbul University, Istanbul, Turkey; 5 Department of Physics, Marmara University, Istanbul, Turkey; Kumamoto University, JAPAN

## Abstract

RNA sequencing (RNA-Seq) is a powerful technique for the gene-expression profiling of organisms that uses the capabilities of next-generation sequencing technologies. Developing gene-expression-based classification algorithms is an emerging powerful method for diagnosis, disease classification and monitoring at molecular level, as well as providing potential markers of diseases. Most of the statistical methods proposed for the classification of gene-expression data are either based on a continuous scale (eg. microarray data) or require a normal distribution assumption. Hence, these methods cannot be directly applied to RNA-Seq data since they violate both data structure and distributional assumptions. However, it is possible to apply these algorithms with appropriate modifications to RNA-Seq data. One way is to develop count-based classifiers, such as Poisson linear discriminant analysis and negative binomial linear discriminant analysis. Another way is to bring the data closer to microarrays and apply microarray-based classifiers. In this study, we compared several classifiers including PLDA with and without power transformation, NBLDA, single SVM, bagging SVM (bagSVM), classification and regression trees (CART), and random forests (RF). We also examined the effect of several parameters such as overdispersion, sample size, number of genes, number of classes, differential-expression rate, and the transformation method on model performances. A comprehensive simulation study is conducted and the results are compared with the results of two miRNA and two mRNA experimental datasets. The results revealed that increasing the sample size, differential-expression rate and decreasing the dispersion parameter and number of groups lead to an increase in classification accuracy. Similar with differential-expression studies, the classification of RNA-Seq data requires careful attention when handling data overdispersion. We conclude that, as a count-based classifier, the power transformed PLDA and, as a microarray-based classifier, vst or rlog transformed RF and SVM classifiers may be a good choice for classification. An R/BIOCONDUCTOR package, MLSeq, is freely available at https://www.bioconductor.org/packages/release/bioc/html/MLSeq.html.

## Introduction

Transcriptome sequencing (RNA-Seq), with the advent of high-throughput NGS technologies, has become a popular experimental approach for generating a comprehensive catalog of protein-coding genes and non-coding RNAs and examining the transcriptional activity of genomes. Furthermore, RNA-Seq is a promising tool with a remarkably wide range of applications such that (i) discovering novel transcripts, (ii) detecting/quantifying the spliced isoforms, (iii) fusion detection and (iv) revealing sequence variations (e.g, SNPs, indels) [1]. In addition, beyond these common applications, RNA-Seq can be a method of choice for gene-expression-based classification to identify the significant transcripts, distinguishing biological samples and predicting the outcomes from large-scale gene-expression data which can be generated in a single run.This classification is widely used in medicine for diagnostic purposesand refers to the detection of a small subset of genes that achieves the maximal predictive performance. These genes are used afterwards for the classification of new observations into one ofthe disease classes (or tumor classes, cancer subtypes, cancer stage, etc.).

Microarray-based gene-expression classification has been widely used during the last decades. Recently, RNA-Seq replaced microarrays as the technology of choice in quantifying gene expression due to certain advantages such as providing less noisy data, detecting novel transcripts and isoforms or not requiring prearranged transcripts of interest [2–5]. Although microarray and RNA-Seq technologies can be used for measuring the expression levels of genes, there are differences in the resulting gene-expression data. Microarray technology produces continuous data while it is obtained in dicrete scale from RNA-Seq technology, which are related with the abundance of mRNA transcripts [6]. Hence, the algorithms which are proposed for microarray-based gene-expression data cannot be directly applied to RNA-Seq data since they violate both data structure and distributional assumptions. In addition, RNA-Seq generates gene-expression data with overdispersion where the variance exceeds the mean [7]. One should take overdispersion into account since it has a significant effect on model performances. Various studies have been conducted to deal with the overdispersion problem for the differential-expression (DE) analysis of RNA-Seq data [8–12].

Several alternatives have been proposed for the classification and clustering of RNA-Seq data. One alternative, perhaps the preferable option,is to use discrete probability distributions (e.g. Poisson, negative binomial) for both classification and clustering tasks. Witten et al. [6] proposed sparse Poisson linear discriminant analysis (PLDA) by extending the popular microarray classifier called the nearest shrunken centroids algorithm to discrete RNA-Seq data. The authors also suggested applying a power transformation within PLDA algorithm in order to handle overdispersion problem. Dong et al. [13], on the other hand, proposed negative binomial linear discriminant analysis (NBLDA) by extending Poisson distribution to negative binomial distribution. There are few methods based on discrete distributions compared to those on continuous distributions. Hence, another choice may be to use some transformation approaches (e.g. vst: variance stabilizing transformation, or rlog: regularized logarithmic transformation) to bring RNA-seq samples closer to microarrays and apply microarray-based algorithms for classification applications [7–9].

In this study, we applied algorithms based on both discrete and continuous distributions to RNA-Seq data. The NBLDA and PLDA are appliedto discrete gene-expression data (i.e, no transformation on counts), while support vector machines (SVM), bagging support vector machines (bagSVM), random forests (RF) and classification and regression trees (CART) are applied to transformed gene-expression data. A comprehensive simulation study is conducted to measure the effect of several parameters on model performances, such as overdispersion, sample size, number of genes, number of classes, DE rate and the transformation method. Four publicly available gene-expression datasetswere also analyzed and the results were compared to the simulation results. An R/BIOCONDUCTOR package, called MLSeq, is developed to analyze RNA-Seq data using the proposed algorithms in this paper.

## Materials and methods

### A workflow for RNA-Seq classification

**Fig 1:**
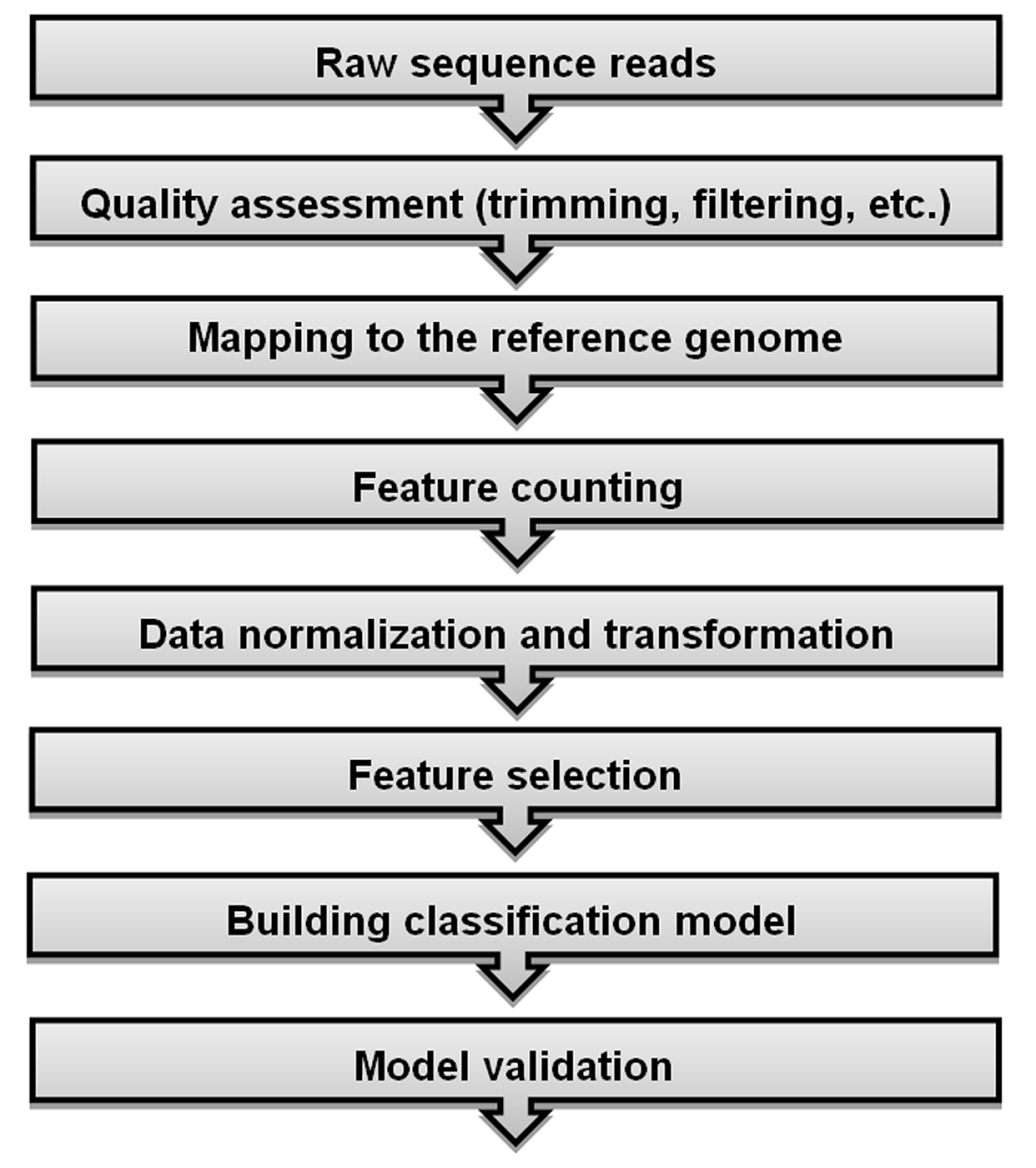
RNA-Seq classification workflow.

Providing a comprehensive and easy-to-understand workflow for RNA-Seq studies and its related algorithms helps researchers to find out the background of such studies. We outlined the count-based classification pipeline for RNA-Seq data in Fig 1 for providing a quick snapshot view of handling large-scale transcriptome data and establishing robust inferences by using well-known computer-aided learning algorithms. NGS platforms generate millions of raw sequence reads along with quality scores which correspond to each base-call. The very first step in RNA-Seq data analysis is to assess the quality of the sequenced data for further analysis. A number of pre-processing steps such as removal of the low-quality sequences, exclusion of the poor-quality reads with more than five unknown bases and trimming the sequencing adapters and primers should be taken into consideration to obtain a clean and ready to use RNA-Seq data for downstream analysis. Several tools/packages such as FASTQC (http://www.bioinformatics.babraham.ac.uk/projects/fastqc/), HTSeq [14], R ShortRead package [15], PRINSEQ (http://edwards.sdsu.edu/cgi-bin/prinseq/prinseq.cgi), FASTX Toolkit (http://hannonlab.cshl.edu/fastx_toolkit/) and QTrim [16] are available for quality assessment and filtering. Following pre-processing steps, high-quality reads are aligned to a reference genome or transcriptome. The number of reads mapped to the reference genome is reported to be linearly related to transcript abundance [6]. Hence, transcript quantification, which are calculated from the total number of mapped reads, is a prerequisite for further analysis. Splice-aware aligners such as Tophat2 [17], MapSplice [18] or Star [19] can be preferred rather than unspliced aligners (BWA, Bowtie, etc.) for aligning short reads. Number of mapped reads to each transcript is counted after the alignment process is completed. This count matrix can be accomplished by using several tools such as HTSeq [14], bedtools [20] and FeatureCounts [21]. The mapped counts are used as the expression levels of corresponding genes and the obtained count matrix is transferred to following steps for downstream analysis. However, these counts cannot be directly used for further analysis since there exists between sample differences in the count matrix. One should normalize the counts to adjust between-sample differences using one of the proposed normalization techniques. Although there are several methods in the literature, there is no standard or state-of-art method for the normalization task. Some of the proposed methods, which are popular and frequently used in RNA-Seq studies, are the deseq median ratio [8], trimmed mean of M values (TMM) [22], reads per kilobase per million mapped reads (RPKM) [23] and quantile [24] methods. The normalized gene-expression data can be used for classification and/or clustering tasks. As previously mentioned, there are two strategies for modeling RNA-seq data. Firstly, normalized counts can be directly modeled using algorithms based on discrete distributions such as the PLDA [6] and NBLDA [13]. Secondly, the counts are transformed to a continuous scale and microarray-based classification and clustering algorithms are performed on the transformed gene-expression data. Some of the popular transformation methods are vst [8], rlog [10] and voom [25]. Apart from these approaches, power transformation is considered to decrease the dispersion of data before applying the PLDA classifier [6].

The crucial steps of classification can be considered as feature selection, model fitting and validation. RNA-Seq data has a large dimensions, i.e there are a large number of genes in the count data. In the feature selection step, we aim to work with an optimal subset of data in order to overcome the curse of dimensionality. This process is also crucial to reduce the computational cost, decrease noise, improve the accuracy for classification of phenotypes and work with more interpretable features to better understand the domain [26]. As the number of variables increases, the model becomes more complex and the classification results are more likely to be over/under-estimated. Various feature selection methods have been reviewed in details and compared in [27]. After an optimal set of genes is selected, the next step is fitting the best classification model to the RNA-Seq data. Several machine-learning algorithms are applied to training data to find the best model which best discriminates cases among classes. Next, the trained model can be used to predict the class memberships of new biological samples. Some of the commonly used classifiers are SVM, RF and other tree-based classifiers, artificial neural networks and k-nearest neighbors.

In many real life problems, it is possible to find classification algorithms which perform well and perfectly classify training samples. However, it may perform poorly when classifying new samples. This problem is called overfitting/overestimation as previously mentioned. This problem arises during the model training steps. In order to overcome overfitting problem and obtain generalized results, the appropriate model is selected by validating the selected model on independent test samples. Holdout, *k*-fold cross-validation, leave-one-out cross-validation and bootsrapping are among the recommended approaches for model validation.

### Simulation study

#### Simulation setup

A comprehensive simulation study is conducted to investigate the effect of several parameters. Gene-expression data are simulated under 864 different scenarios using a negative binomial model as follows:

$$X_{ij}|y_{i}=k∼NB(s_{i}g_{j}d_{kj},\phi )$$ (1)

where, *g*_*j*_ is the total number of mapped counts per gene (i.e, gene total), *s*_*i*_ is the number of mapped counts per sample, *d*_*kj*_ is the differential-expression parameter of the *j*^*th*^ gene between classes *k* and *φ* is the dispersion parameter. The datasets contain all possible combinations of the following:

- different dispersion parameters such as *φ* = 0.01 (very slightly overdispersed), *φ* = 0.1 (substantially overdispersed), and *φ* = 1 (highly overdispersed);
- number of biological samples (*n*) changing as 40, 60, 80, 100;
- number of differentially-expressed genes (*p’*) as 25, 50, 75, 100;
- differentially expressed gene rates as (*d*_*kj*_) 1%, 5% and 10%;
- number of classes (*k*) as 2, 3 and 4;
- method of transformation as *rlog* and *vst*.

In the simulation setup, *s*_*i*_ and *g*_*j*_ are distributed identically and independently. Simulated datasets are generated using the CountDataSet function in the PoiClaClu package of R software [28] and manipulated based on the details given above. The seed number for random number generation is set to ‘10072013’ in all analysis steps.

#### Evaluation process

All datasets are initially simulated for *p* = 10,000 genes. Next, the data are split into training (70%) and test sets (30%). All the model building processes are performed on training datasets and model performances are evaluated in test sets.

#### Size factor estimation

The size factors are estimated using deseq median ratio approach [10]. Let *x*_*ij*_, the mapped read counts to *j*^th^ gene for *i*^th^ sample. Size factor of the *i*^*th*^ sample (${\hat{s}}_{i}$) can be estimated as below:

$$m_{i}={median}_{i}\left\{\frac{x_{ij}}{{\left(\prod_{i=1}^{n}x_{ij}\right)}^{\frac{1}{n}}}\right\}$$ (2)

$${\hat{s}}_{i}=\frac{m_{i}}{\sum_{i=1}^{n}m_{i}}$$ (3)

Size factor of a test sample can be estimated using the same parameters as for the training datasets. In more detail, the size factors of the test datasets are calculated based on the geometric means of the training data. Therefore, we guarantee that the training and test datasets are in the same scale and homoscedastic to each other. Let, x_*_ is a count vector of a new test observation, whose class label y_*_ will be predicted. Size factor of the test sample (${\hat{s}}_{*}$) is estimated as follows:

$$m_{*}={median}_{g}\left\{\frac{x_{g*}}{{\left(\prod_{i=1}^{n}x_{gi}\right)}^{1/n}}\right\}$$ (4)

$${\hat{s}}_{*}=\frac{m_{*}}{\sum_{i=1}^{n}m_{i}}$$ (5)

#### Dispersion function estimation

After size factor estimation, the datasets are transformed using either rlog or vst transformation for the SVM, bagSVM, RF and CART algorithms. The logarithmic transformation approach transforms the data into a less skewed distribution with less extreme values as well; however, the genewise variances are still unstabilized [10]. In vst transformation, a local dispersion function is fit to the training data. This function is frozen to reapply for the test samples. In rlog transformation, the log fold changes of the counts for each gene are regularized over an intercept. Rlog transformation is applied as follows:

$$rlog(q_{ij})=\beta_{0j}+\beta_{ij}$$ (6)

The dispersion function, beta prior variance and the intercept which are calculated from the training data are stored and directly used for the test dataset. More details can be found in DESeq2 paper [10].

#### Filtering

Next, we applied near-zero variance filtering to training data to filter the genes with low counts. The effect of the filtered genes is eliminated for further analysis [29]. Genes are filtered based on two criteria: (i) the frequency ratio of the most frequent value to the second most frequent value is higher than 19 (95/5), and (ii) the ratio of the number of unique values to the sample size is less than 10%. Filtered genes are removed from both the training and test sets. Next, the DESeq2 method is applied to detect the most DE 25, 50, 75 and 100 genes [10]. The same genes are selected for both test and training sets.

#### Normalization and transformation

After selecting the DE genes, training data are normalized with the estimated size factors to adjust sample specific differences [10]. After normalization, the datasets are transformed with the estimated dispersion functions using either rlog or vst transformation for the SVM, bagSVM, RF and CART algorithms. The normalized count datasets are directly used for the PLDA and NBLDA algorithms since both algorithms use discrete probability distributions to fit the models. Although the NBLDA takes overdispersion into account, the PLDA does not estimate the overdispersion parameter. Hence it assumes that there is no overdispersion in the data. Witten [6] suggested the use of power transformation on the raw counts when there is slight to moderate overdispersion in the data. Power transformation is useful to remove overdispersion in the data in such cases. However, one should explicitly estimate and consider overdispersion when data are highly overdispersed. In this paper, we performed power transformation for slightly or moderately overdispersed data. The results are given under PLDA_2_.

#### Model building

After the normalization and transformation processes, the parameters of each classifier are optimized to avoid overfitting and underfitting. A five-fold cross-validation is applied to the training data and the parameters that achieve the highest accuracy rate are selected as optimal parameters. Cross-validation folds are fixed for each classifier to make the results comparable. Each classifier is fitted with the optimal parameters. Fitted models are used in the test datasets for prediction and performance evaluation. The sample sizes are very low relative to the number of genes since we mimic the real datasets. Thus, the model performances may vary depending on the split ratio of the training and test sets. To overcome this limitation, we repeated the entire process 50 times and summarized the results in a single statistic, i.e. accuracy rates.

### Application to real datasets

In addition to the simulated data, four real datasets, including both miRNA and mRNA datasets were also used as real life examples (Table 1).

**Table 1:** Description of real RNA-Seq datasets used in this study.

Dataset	Number of groups	Sample size	Number of features
Cervical cancer [39]	2	58 (29 cervical cancer, 29 control)	714 miRNAs
Alzheimer [40]	2	70 (48 Alzheimer, 22 control)	416 miRNAs
Renal cell cancer [41]	3	1,020 (606 KIRP, 323 KIRC, 91 KICH)	20,531 mRNAs
Lung cancer [41]	2	1,128 (576 LUAD, 552 LUSC)	20,531 mRNAs

#### Cervical dataset

The cervical dataset is an miRNA sequencing dataset obtained from [30]. miRNAs are small non-coding RNA molecules with an average length of 21–23 bp. These small molecules regulate the gene expression levels. The objective of this study was to identify the novel miRNAs and detect the differentially expressed ones between normal and tumor cervical tissue samples. For this purpose, 58 small RNA libraries are constructed (29 with tumor and 29 without tumor). Among the29 tumor samples, 21 were diagnosed as squamous cell carcinoma, 6 of them were adenocarcinomas and 2 were unclassified. In our analysis, we used the gene-expression levels of 714 miRNAs belonging to 58 human cervical tissue samples.

#### Alzheimer dataset

This dataset is another miRNA dataset provided by Leidinger et al. [31]. The authors aimed to discover potential miRNAs from blood in diagnosing Alzheimer and related neurological diseases. For this purpose, the authors obtained gene-expression data from 48 Alzheimer patients who were evaluated after undergoing some tests and 22 age-matched control samples. RNA sequencing is performed using an Illumina HiSeq2000 platform. The miRNAs with less than 50 counts in each group are filtered. We used the data including 416 miRNA read counts of 70 samples, where 48 Alzheimer and 22 control samples are considered as two separate classes for classification.

#### Renal cell cancer dataset

The renal cell cancer (RCC) dataset is an RNA-Seq provided by The Cancer Genome Atlas (TCGA) [32]. The TCGA is a comprehensive community resource platform for researchers to explore, download, and analyze datasets. RCC data contain 20,531 known human RNA transcript counts belonging to 1,020 RCC samples. These RNA-Seq data include 606 kidney renal papillary cell (KIRP), 323 kidney renal clear cell (KIRC) and 91 kidney chromophobe carcinoma (KICH) samples. These three classes are known as the most common subtypes of RCC and treated as three separate classes in our analysis [33].

#### Lung cancer dataset

Lung cancer is another RNA-Seq dataset provided by the TCGA platform. This dataset contains the read counts of 20,531 transcripts of 1,128 samples. Samples are separated into two distinct subclasses. These subclasses are lung adenocarcinoma (LUAD) and lung squamous cell with carcinoma (LUSC) with 576 and 552 class sizes, respectively. These two classes are used as class labels in our analysis.

#### Evaluation process

Real datasets are analyzed using similar procedures to those in the simulation study. Model building is performed on the training set (70%) and the test set (30%) is used to evaluate model performance. Size factors and dispersion functions are estimated for training datasets. Similar to the simulation experiments, the size factors and dispersion functions of test datasets are directly estimated from the training data to make them in the same scale and homoscedastic to each other. Near-zero variance filtering is applied to the training set. Filtered genes are also removed from the test set. The renal cell and lung cancer datasets include 20,531 features which dramatically increase the computational cost. Hence, we initially selected 5,000 genes with the highest variances to eliminate the effect of non-informative mRNAs and decrease the computational cost. All of the miRNAs are used in the model building process for the cervical and Alzheimer datasets. Differential expression was performed on the training data using the DESeq2 method and genes are ranked from the most significant to the least significant with increasing number of genes in steps of 25 up to 250 genes. The differentially expressed genes selected in the training data are also selected in the test datasets. Differentially expressed genes in the training data are normalized using the median ratio approach and transformed using either the vst or rlog approaches. Since the sample sizes of the cervical and Alzheimer miRNA datasets are relatively small, the entire process is applied 50 times. The other model building processes applied are similar tothose in the simulation study.

### Implementation of classifiers

Both simulated and real data are modeled using support vector machines (SVM), bagging support vector machines (bagSVM), random forests (RF), classification and regression trees (CART), Poisson linear discriminant analysis without power transformaton (PLDA_1_), Poisson linear discriminant analysis with power transformaton (PLDA_2_) and negative binomial linear discriminant analysis (NBLDA). In this section, we will summarize the background and use of each method.

#### SVM

SVM is among popular classification methods based on the statistical learning theory [34]. It has attracted great attention because of its strong mathematical background, learning capability, good generalization ability and wide range of application area such as computational biology, text classification, image segmentation and cancer classification [34,35]. SVM is capable of linear/nonlinear classification and deals with high-dimensional data.

Let *x*_*i*_ denotes the training data points, *w* denotes the weight vector and *b* denotes the bias term.The decision function that correctly classifies the data points by their true class labels in a linearly separable space is represented as follows:

$$f_{w,b}=sign(w.x_{i}+b)\;i=1,2,\ldots ,n$$ (7)

In a binary classification, the SVM aims to find an optimal separating hyperplane in the feature space which maximizes the margin and minimizes the misclassification rate by choosing the optimum value of *w* and *b* in Eq (7). When the cases are not linearly separable, “slack variables” {*ξ*_1_,…,*ξ*_*n*_}, a penalty term which is proposed by Cortes and Vapnik [36] can be used to allow misclassified data points where *ξ*_*i*_ > 0. In most of theclassification problems, the separation surface is not linear. In this case, the SVM uses an implicit mapping Φ of the input vectors to a high-dimensional space defined by a kernel function (*K*(*x*,*y*) = Φ(*x*_*i*_)Φ(*x*_*j*_)) and the linear classification then applied in this high-dimensional space. Some of the most widely used kernel functions are linear: *K*(*x*,*y*) = *x*_*i*_*x*_*j*_, polynomial: *K*(*x*,*y*) = (*x*_*i*_*x*_*j*_ + 1)^*d*^, radial basis function: *K*(*x*,*y*) = *exp* (−*γ*‖*x*_*i*_−*x*_*j*_‖^2^) and sigmoidal: *K*(*x*,*y*) = *tanh*(*k*(*x*_*i*_*x*_*j*_) − *c*) where *c* is a constant, *d* is the degree and *γ* > 0 is sometimes parametrized as *γ* = 1/2*σ*^2^. Normalized and transformed (either using vst or rlog) datasets are used as input to the SVM classifier. The radial basis kernel function is used in the analysis.

#### bagSVM

bagSVM is a bootstrap ensemble extension of SVM which creates individuals for its ensemble by training each SVM classifier on a random subset of the training set. For a given data set, *k* random bootstrap samples are drawn with replacement. SVM classifiers are trained independently on each randomly selected subsets and aggregated via an aggregation technique.A test set is predicted on each of the SVM classifiers and the predicted class labels are determined using aggregated results likely in training sets. Normalized and transformed datasets are used as input to the bagSVM classifier. The number of bootstrap samples were set to 101 since small changes were observed over this number.

#### CART

CART, which was introduced by Breiman [37], is one of the most popular tree classifiers with a wide range of applications. It uses the Gini index, which maximizes the decrease in impurity at each node, to find the optimal path. If *p*(*i*|*j*) is the probability of class *i* at node *j*, the Gini index is calculated using the equation 1 − ∑_*i*_*p*^2^(*i*|*j*). It is possible to obtain very large CART trees in large data sets, i.e very large number of genes and samples. When CART grows a maximal tree, this tree is pruned upward to get a decreasing sequence of subtrees. Furthermore, pruning is preferred to overcome overfitting problem. The optimal tree that has the lowest misclassification rate is selected using a cross-validation.The assignment of each terminal node to a class is performed by choosing the class that minimizes the resubstitution estimate of the misclassification probability [37, 38]. Normalized and transformed datasets are used as input to the CART classifier.

#### RF

An RF is a collection of many CART trees combined by averaging the predictions of individual trees in the forest [39]. RF aims to combine many weak classifiers to produce a significantly better and strong classifier. First, training set is generated by drawing a bootstrap sample from the original data. This bootstrap sample includes 2/3 of the original data. The remaining partisused as a test set to predict the out-of-bag error of the classification. A subset of features are randomly selected at each node and the best split is used to split the corresponding nodes. If there are *m* features, for example, *m*_*try*_ out of *m* features is randomly selected at each node while growing the forest. Different splitting criterias can be used such as the Gini index, information gain and node impurity. The value of *m*_*try*_ is approximately equal to either $\frac{\sqrt{m}}{2}$, $\sqrt{m}$ or $2\sqrt{m}$ and is constant during the forest growing. Unlike CART, an unpruned tree is grown for each of the bootstrap samples. Finally, class labels of new cases are predicted by aggregating (i.e. majority voting) the predictions fromall trees [40, 41]. Normalized and transformed datasets are used as input to the RF classifier. The number of trees was set to 500 in the analysis.

#### PLDA_1_ and PLDA_2_

Let ***X*** be an *n* × *p* matrix of the sequencing data where *n* is the number of observations and *p* is the number of features. For sequencing data, *X*_*ij*_ indicates the total number of reads mapping to gene *j* in observation *i*. The observed counts are fitted to the Poisson log-linear model as given in Eq (8),

$$X_{ij}∼Poisson(N_{ij}),\;N_{ij}=s_{i}g_{j}$$ (8)

where *s*_*i*_ is the total number of reads per sample and *g*_*j*_ is the total number of reads per region of interest. For RNA-seq data, Eq (8) can be extended as follows:

$$X_{ij}|y_{i}=k∼Poisson(N_{ij}d_{jk}),\;N_{ij}=s_{i}g_{j}$$ (9)

where *y*_*i*_ ∈ {1,…,*K*} is the class label of the *i*^*th*^ observation. The *d*_1*j*_,…,*d*_*Kj*_ terms allow the *j*^*th*^ feature to be differentially expressed between classes.

Let (*x*_*i*_,*y*_*i*_), *i* = 1,…,*n* be a training set and $x^{*}=(X_{1}^{*},\ldots ,X_{p}^{*}{)}^{T}$ be a test set. A new sample x* is assigned to one of the classes with highest probability(or discrimination score) using the Bayes’ rule as follows:

$$P(y^{*}=k|x^{*})\propto f_{k}(x^{*})\pi_{k}$$ (10)

where y* denotes the unknown class label, *f*_*k*_ is the probability density of an observation in class *k* and *π*_*k*_ is the prior probability that an observation belongs to class *k*. If *f*_*k*_ is a Gaussian density with a class-specific mean and common variance, then a standard LDA is used to assign a new observation to the class [42]. When the model uses class-specific mean and a common diagonal matrix, then diagonal LDA is used for the classification [43]. However, the normality and common covariance matrix assumptions are not appropriate for sequencing data. Witten [6] assumes that the data follow a Poisson model as given in Eq (11),

$$X_{ij}|y_{i}=k∼Poisson(N_{ij}d_{kj}),\;N_{ij}=s_{i}g_{j}$$ (11)

where *y*_*i*_ is the class of the *i*^*th*^ observation and the features are independent. Eq (9) specifies that $X_{j}^{*}|y^{*}=k∼Poisson(s^{*}g_{j}d_{kj})$. First,

$$logP(\hat{y^{*}=k|}x^{*})=\log{\hat{f}}_{k}(x^{*})+\log{\hat{\pi }}_{k}+c=\sum_{j=1}^{p}X_{j}^{*}log\hat{d_{kj}}-s^{*}\sum_{j=1}^{p}{\hat{g}}_{j}log{\hat{d}}_{kj}+\log{\hat{\pi }}_{k}+c^{\prime }$$ (12)

where *c* and *c*′ are constants and do not depend on the class label. A new observation is assigned to one of the classes for which Eq (12) is the largest [6].

Normalized count data are used as input to the PLDA_1_ classifier. After normalization, a power transformation ($X_{ij}^{\prime }=\sqrt{X_{ij}+3/8}$) is applied to reduce the overdispersion effect and make genes have constant variance. These normalized and power transformed datasets are used as input to the PLDA_2_ classifier. To optimize the tuning parameter, a grid search (30 searches) is applied and the sparsest model with the highest accuracy rates is selected for classification.

#### NBLDA

Dong et al. [13] generalized the PLDA using an extra dispersion parameter (*φ*) of negative binomial distribution and called the method negative binomial linear discriminant analysis (NBLDA). This extra dispersion parameter is estimated using a shrinkage approach detailed in [44]. A new test observation will be assigned to its class based on the following NBLDA discriminating function:

$$\begin{array}{l} logP(\hat{y^{*}=k|}x^{*})=\sum_{j=1}^{p}X_{j}^{*}[log\hat{d_{kj}}-log(1+s^{*}{\hat{g}}_{j}d_{kj}\phi_{j})]\\ \;-\sum_{j=1}^{p}\phi_{j}^{-1}\log\left(1+s^{*}{\hat{g}}_{j}d_{kj}\phi_{j}\right)+\log{\hat{\pi }}_{k}+c^{\prime } \end{array}$$ (13)

As the dispersion decreases, NBLDA approximates to PLDA. More details on NBLDA can be found in [13].

#### Evaluation criteria

To validate each classifier model, five-fold cross-validation is used. It is repeated 10 times and accuracy rates are calculated to evaluate the performance of each model. Cross-validation folds are fixed for all classifiers to make the results comparable to each other. Accuracy rates are calculated as (*TP* + *TN*)/*n* based on the confusion matrices of test set class labels and test set predictions. For multiclass scenarios, these measures are calculated via the one-versus-all approach. Since, the class sizes are unbalanced in the Alzheimer and renal cell cancer datasets, accuracies are balanced using the following formula: (*Sensitivity* + *Specificity*)/2.

### MLSeq R/BIOCONDUCTOR package

We presented an R package in the BIOCONDUCTOR network for classification of the RNA-seq data. The MLSeq package accepts gene expression data which can be obtained from feature counting tools (e.g. HTSeq [14], bedtools [20] and FeatureCounts [21] etc.). It also has the ability to normalize and transform the gene-expression data. Finally, data are fitted to the selected model such as SVM, bagSVM, RF and CART. Users can access the MLSeq package from https://www.bioconductor.org/packages/release/bioc/html/MLSeq.html.

## Results and discussion

**Fig 2:**
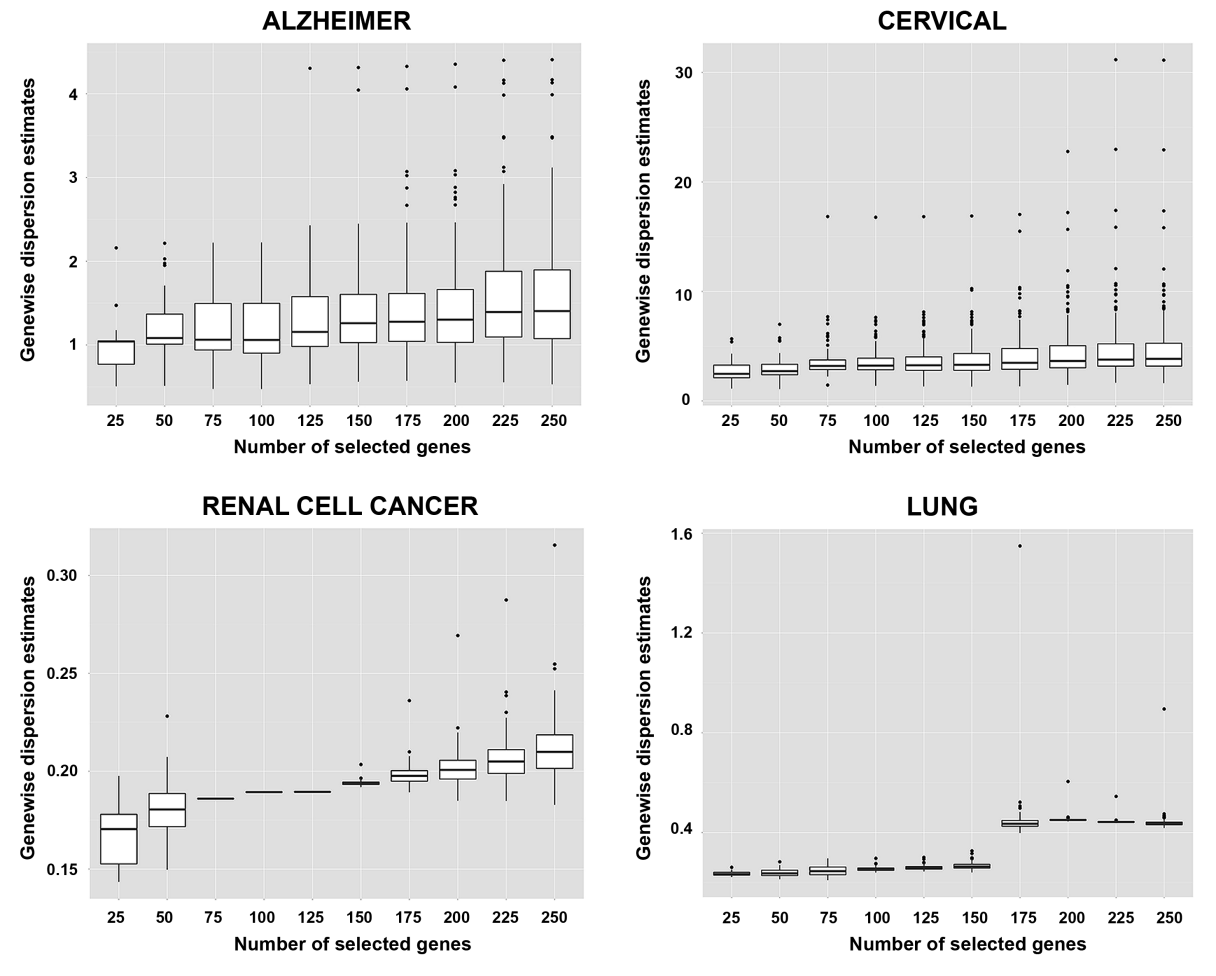
Genewise dispersion estimations for real datasets.

**Fig 3:**
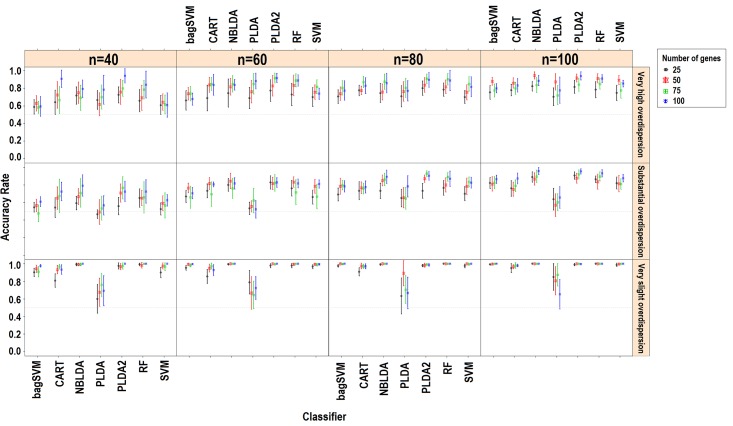
Simulation results for *k* = 2, *d*_*kj*_ = 10%, transformation: rlog. Figure shows the performance results of classifiers with changing parameters of sample size (*n*), number of genes (*p*) and type of dispersion (*φ = 0*.*01*: very slight, *φ = 0*.*1*: substantial, *φ = 1*: very high).

**Fig 4:**
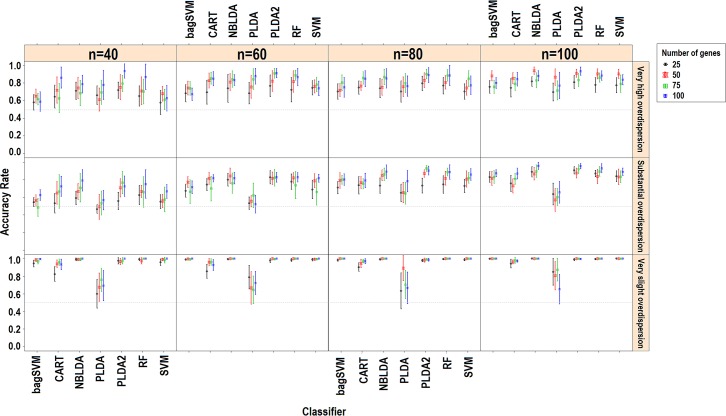
Simulation results for *k* = 3, *d*_*kj*_ = 10%, transformation: rlog. Figure shows the performance results of classifiers with changing parameters of sample size (*n*), number of genes (*p*) and type of dispersion (*φ = 0*.*01*: very slight, *φ = 0*.*1*: substantial, *φ = 1*: very high).

**Fig 5:**
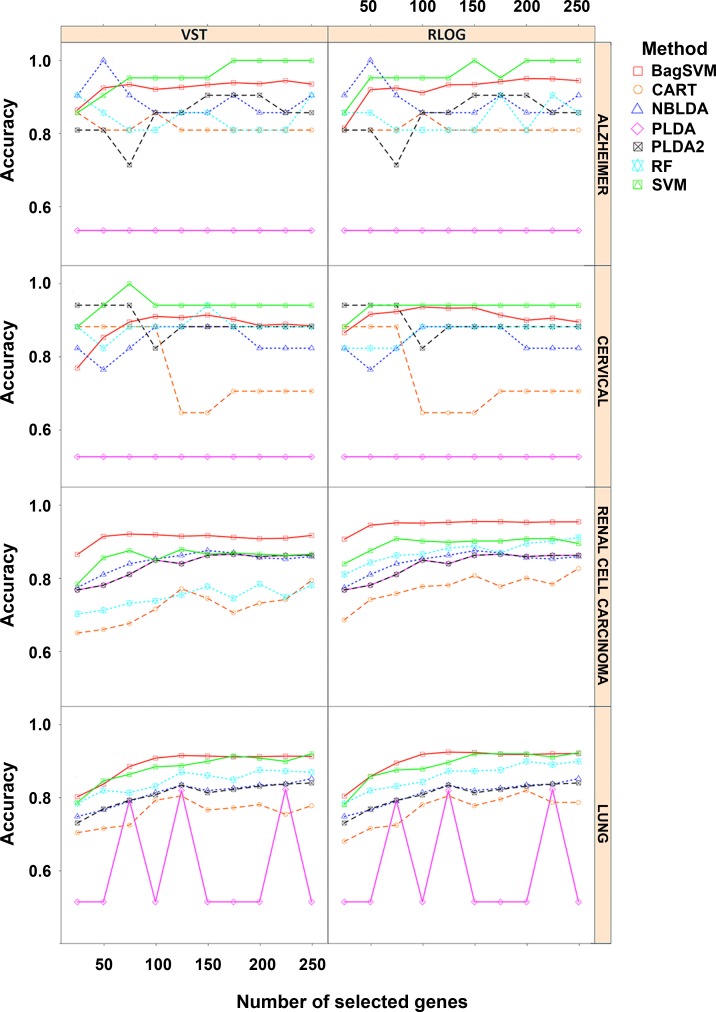
Results obtained from real datasets. Figure shows the performance results of classifiers for datasets with changing number of most significant number of genes. Note that PLDA and NBLDA methods are not performed on the transformed data. However, the results for both transformed and non-transformed data are given in the same figure for the comparison purpose.

Genewise dispersion parameters are estimated for each classifier usingthe method of moments. Distribution of the estimated overdispersions is given in Fig 2. It is seen from the figure that the cervical and Alzheimer miRNA datasets are very highly overdispersed (*φ*>1), while the lung and renal cell cancer datasets are substantially overdispersed. The simulation results for *k* = 2 and *k* = 3, *d*_*kj*_ = 10% for rlog transformations are given in Fig 3 and Fig 4. All other simulation results are given in http://www.biosoft.hacettepe.edu.tr/MLSeqSupplementary/ and the S1 File. Results for real datasets are given in Fig 5.

### Effect of simulation parameters

Since combining each significant gene on class conditions is equivalent to combining their predictive abilities, an increased number of differentially expressed genes leads to an increase in the classification accuracy (Fig 2 and Fig 3). Similarly, in most scenarios, working with more samples and genes has a positive impact on the overall model accuracies. This relationship between the number of genes and accuracy is mostly available when *d*_*kj*_ = 10%. Likewise, classification accuracies slightly increase in real datasets since increasing the number of genes leads to an increase in the probability of a differentially expressed gene being included in the classification model. However, this may not be true for all cases. Since, the classification accuracy may decrease from certain point as more genes were included in the model. Since, increasing the number of genes will lead to an increase in the model complexity, we expect that the classification error of test samples will be reduced. For the PLDA classifier, a high number of selected genes provides alternative options for the lasso shrinkage method to test more genes in classification models. The RF algorithm builds trees with the bagging approach. As the number of genes increases, the RF algorithm uses more genes and may perform better to specify the optimal tree. Increasing the sample size, on the other hand, improves the discrimination power and classification accuracy. Conversely, overall accuracies decrease as the number of classes increases. This is due to the fact that the probability of misclassifying an observation may arise depending on the complexity of the model. As the number of classes increases, the samples become less likely to be linearly (or non-linearly) separable.

### Effect of dispersion on classification accuracies

The overall performance of each model increases as the data become less dispersed. Decreasing the dispersion parameter makes a significant contribution to classification accuracy, as expected, even for the same data and the same scenario. This is more obvious when *k* = 2 and *d*_*kj*_ = 10%. As the data become more spread out (i.e, increasing overdispersion), the variance increases; thus, we need more samples to better learn from data and achieve the same discrimination power. When we stabilize the sample size and increase the dispersion parameter, this leads to a decrease in the discrimination power and classification accuracies. Nagalakshmi et al. [7] reported that using biological replicates instead of technical replicates leads to an increase in the dispersion of the data. Based on this idea, increasing the biological variance of the observations results in an increase in the dispersion, which decreases the discrimination power. In DE studies of RNA-Seq data, overdispersion is one of the major problems to be carefully handled. Many studies are available in the literature which focus onthe overdispersion problem [9–10, 25, 45–46]. When we look at the classification accuracy results, overdispersion alsoseems to be a major challenge in classification studies. Unless we work with technical replicates, RNA-Seq data are overdispersed and the mapped counts from different biological replicates on the same gene have variance exceeding the mean [7]. This overdispersion can be seen in other studies [9, 46–49]. In conclusion, the results of our study revealed that overdispersion has a significant and negative effect on classification accuracies and should be taken into account before model building.

### Microarray-based classifiers and transformation effect on classification accuracies

Hundreds of microarray-based classifiers have been developed and are able to work in large *p* and small *n* settings. However, technological improvements make RNA-Seqa state-of-the-art approach for quantified transcriptomics. Currently, much of these microarray-based classifiers cannot be directly applied to RNA-Seq data because of the discrete nature of RNA-Seq data. Microarray data consist of the continuous log-intensities of probes while RNA-Seq data consist of the discrete and overdispersed mapped read counts of sequencing technologies. The results of this study revealed that, transforming the RNA-Seq data and bringing them hierarchically closer tomicroarrays (e.g. through rlog and vst) might be a suitable approach to make the microarray-based classifier applicable for RNA-Seq data.

Witten et al. [6] stated that the normalization strategy has little impact on classification performance but may be important in differential expression analysis. However, data transformation has a direct effect on classification results since it changes the underlying distribution of the data. In this study, we used deseq normalization with vst and rlog transformations and obtained satisfactory classification performances. Love et al. [10] reported that vst transformation does not consider the size factors during transformation. However, in both simulated and real datasets, there were no substantial differences between rlog and vst transformation approaches in terms of classification accuracies. Both transformations can be applied to RNA-Seq data.

### Power transformed PLDA and other count-based classifiers

Without transformation, the PLDA seemed to perform well in very slightly overdispersed datasets. This can be seen in both simulated and real datasets. For instance, in the renal cell carcinoma dataset, the dispersion parameter is very low and the data seem to follow Poisson distribution. In this case, the overdispersion is negligible and no power transformation is needed. Hence, PLDA_1_ and PLDA_2_ show similar performances (Fig 5). However, the performance of this method decreases when the data become more overdispersed. The reason is that the PLDA classifies the data assuming that the underlying distribution is Poisson even though it should be negative binomial. Although the Poisson distribution assumption might be valid after power transformation for moderately overdispersed data, it is invalid for highly overdispersed data. Therefore, based on the results for both simulated and real datasets, we suggest that this transformation is very useful and should be applied for moderately overdispersed data before building the model.When power transformation fails, one should use negative binomial distribution rather than Poisson distribution. The NBLDA extends this classifier using a negative binomial model. We expect that the NBLDA should give better predictive performances compared to the PLDA. However, the classification accuracy of this method is not as high that of the PLDA with power transformation. This might be for several reasons. First, the NBLDA algorithm is not sparse. Hence, it uses more features than the PLDA; as a result of overdispersion, even these genes are not differentially expressed among classes. Second, there are several methods for estimating the overdispersion parameter. The selected method for overdispersion estimation might be another reason for lower predictive accuracy. In conclusion, novel or improved count-based classifiers are still needed for accurate and robust classification of RNA-Seq data.

In lung cancer dataset, the odd behavior of PLDA may appear based on its own built-in variable selection algorithm. In this data set, when PLDA is able to select some of the features in the classification task, it gives higher classification accuracy. However, if this algorithm was not able to select any feature in the discrimination, it classifies all the observations into first class. Hence the accuracy is the ratio of the samples in the corresponding class. However, when PLDA is used with power transformation, the accuracy of the model significantly increases and the variable selection algorithm works well in most of the model fitting process.

### Overall performances of classifiers

In simulated datasets, the power transformed PLDA was found to be the best classifier. The RF and NBLDA performed in a moderately similar manner. On the other hand, the SVM and bagSVM had the highest classification accuracies in real datasets. The PLDA_2_, RF and NBLDA give comparable and high classification accuracies, but they are lower than SVM and bagSVM. These slight differences may arise from the differences between negative binomial distribution, which is used in the simulation settings, and the exact distributions of real RNA-Seq data. In real datasets, SVM and bagSVM classifiers display their classification abilities. Moreover, it can be seen from the simulated and real datasets that the performance of the bagSVM classifier increases as the sample size increases. A possible explanation for such an observation is that the bagSVM uses the bootstrap technique and trains better models in datasets with a high number of samples. We also observed that PLDA_2_ and NBLDA performed more accurate in cervical and alzheimer datasets. Similar with the simulation results, we can say that these two algorithms are more efficient in highly overdispersed datasets. The performances of CART and PLDA_1_ were found to be lower than those of the other classifiers. This result is consistent with the results of simulated data.

All assessments in this study are made based on the classification accuracies. Another important measure may be the sparsity of classifiers. Since we included mostly the non-sparse classifiers in this study, we will leave the effect of the dispersion parameter on sparsity as a topic for further research.

## Conclusions

A considerable amount of evidence collected from genome-wide gene-expression studies suggests that the identification and comparison of differentially expressed genes have been a promising approach for diagnosis and prognosis purposes. Although microarray-based gene-expression studies have been widely used for discovering potential biomarkers related to disease status [50–53], it has limitations in terms of novel transcript discovery and abundance estimation with a large dynamic range. Thus, one choice is to utilize the power of RNA-Seq techniques in the analysis of the transcriptome for diagnostic classification to overcome the limitations of microarray-based experiment. As mentioned in earlier sections, working with less noisy data may improve the predictive performance of classifiers and novel transcripts may be discovered as a new biomarker in the studied disease or phenotypes.

Hundreds of studies have been published on microarray-based classification. The goal of these studies was to develop or adapt novel approaches to identify a small subset of genes and predict the class labels of a new observation. This has particular importance in biomedical studies for the molecular diagnosis of diseases. In this study, we demonstrated how researchers can classify the RNA-Seq data which is the state-of-the-art technique for the quantification of gene expression. We conducted a comprehensive simulation study and also used four real experimental miRNA/mRNA datasets.

Besides RNA-Seq’s advantages over microarrays, the gene-expression data from RNA-Seq are overdispersed due to inherent variability. This overdispersion seemed to be a drawback for differential expression studies of RNA-Seq data. In this study, we showed that this overdispersion is also a drawback for classification studies since an increase in the variance will lead to a decrease in the discrimination power. We reached the conclusion that three solutions are available to handle the classification of overdispersed RNA-Seq data: (i) increasing the sample size, (ii) transforming the data to bring RNA-Seq data hierarchically closer to microarrays usingvariance stabilizers, e.g. vst and rlog transformations and (iii) using count-based classifiers such as the PLDA_2_ and NBLDA. Our simulation study revealed that both microarray-based classifiers after rlog/vst transformations and count-based classifiers (that deal with overdispersion) can be efficiently used for the classification of RNA-Seq data.

To make an overall assessment for the performances of classifiers, the PLDA after a power transformation may be a good choice as a count-based classifier. Furthermore, its sparsity seems to be an advantage for researchers. However, further studies are needed. Surprisingly, the performance of the NBLDA was not satisfactory as a count-based classifier. Dong et al. [13] reported that the NBLDA performs better than the PLDA in moderate and highly overdispersed data. However, these comparisons were made with the same number of genes. Our analyses were performed based on the sparse PLDA classifiers where the best subset of genes is used in classification. The sparse PLDA classifier after a power transformation performed more accurately in all dispersion settings. We believe that extending the NBLDA algorithm into a sparse classifier may improve its classification performance by selecting the most significant genomic features.

Moreover, an alternative option may be to bring the data closer to microarrays and use microarray-based classifiers. Our results revealed that the RF, SVM and bagSVM may give accurate results after an rlog or vst transformation. Moreover, the efficiency of the bagSVM is improved markedly with increasing sample size.

We conclude that data with less overdispersion, highly differentially expressed genes, a lower number of groups and large sample size may improve the accuracy of the classifiers. Finally, we developed an R/BIOCONDUCTOR package, called MLSeq, for the classification of RNA-Seq data. This package can be accessed and downloaded through https://www.bioconductor.org/packages/release/bioc/html/MLSeq.html.

## Supporting information

S1 File All figures for simulation results. (ZIP)

S2 File MLSeq package source. (ZIP)

S3 File Simulation R codes. (ZIP)

S4 File Computational infrastructure. (DOCX)

S5 File Computational costs of classifiers. (DOCX)

## Abbreviations

RNA: Ribonucleic acid
NGS: Next-generation sequencing
PLDA: Poisson linear discriminant analysis
NBLDA: Negative binomial linear discriminant analysis
DE: Differential expression
vst: Variance stabilizing transformation
rlog: Regularized logarithmic transformation
SVM: Support vector machines
bagSVM: Bagging support vector machines
CART: Classification and regression trees
RF: Random forests
BWA: Burrows-Wheeler algorithm
TMM: Trimmed mean of M values
RPKM: Reads per kilobase per million mapped reads
NB: Negative binomial
ADAS-Cog: Alzheimer Disease Assessment Scale-cognitive subscale
WMS: Wechsler Memory Scale
MMSE: Mini-Mental State Exam
CDR: Clinical Dementia Rating
RCC: Renal cell cancer
TCGA: The Cancer Genome Atlas
KIRP: Kidney renal papillary cell
KIRC: Kidney renal clear cell
KICH: Kidney chromophobe carcinomas
LUAD: lung adenocarcinoma
LUSC: lung squamous cell with carcinoma
