# Supplementary figures and images for: A comprehensive simulation study on classification of RNA-Seq data
Source: PLoS One. 2017 Aug 23;12(8):e0182507. doi: 10.1371/journal.pone.0182507 (PMC5568128; doi:10.1371/journal.pone.0182507)

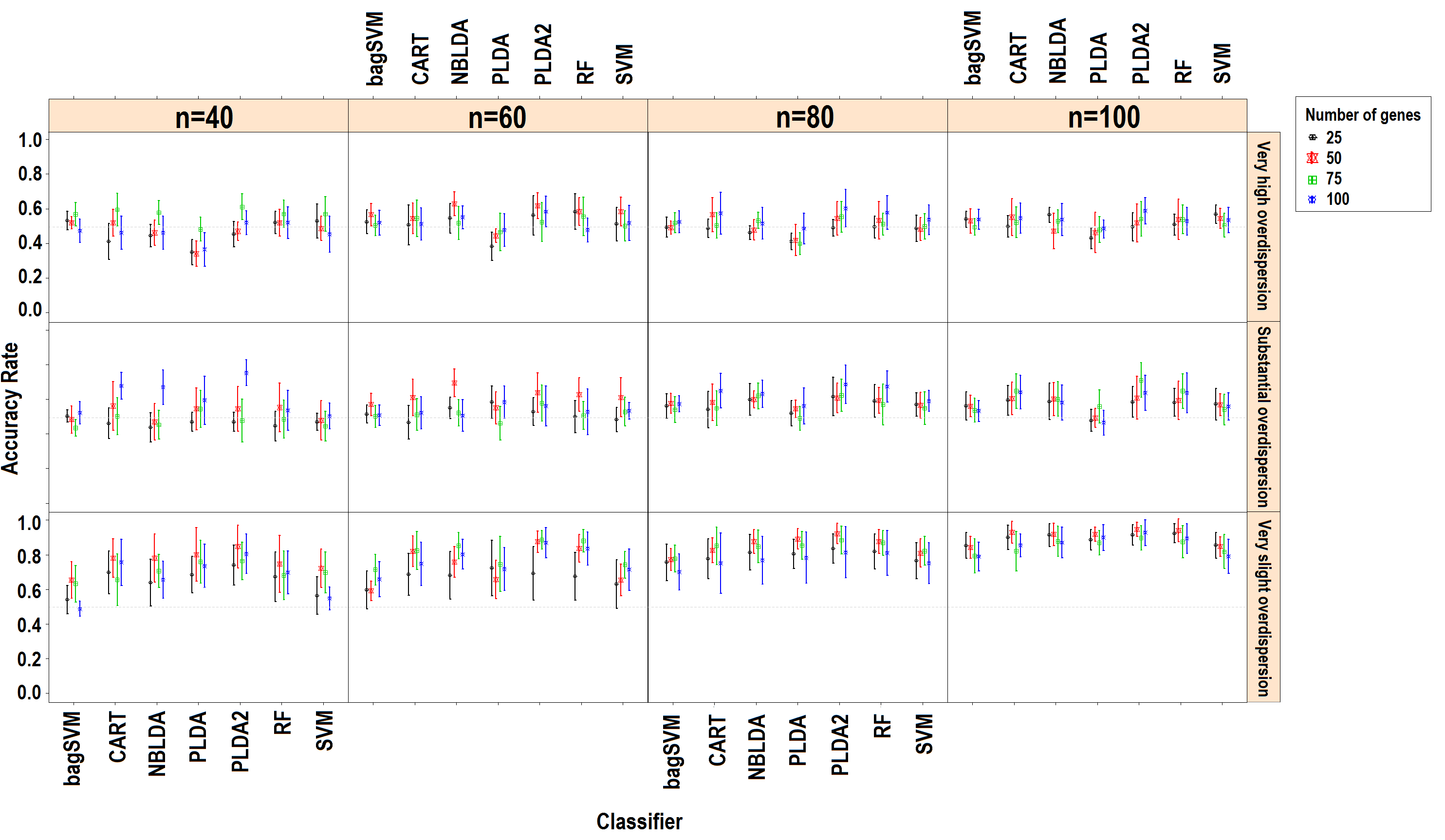

Supplement: S1 File — (ZIP) [file pone.0182507.s001.zip › Additional File 1ΓÇô All figures for simulation results/0.01rlog2.png]

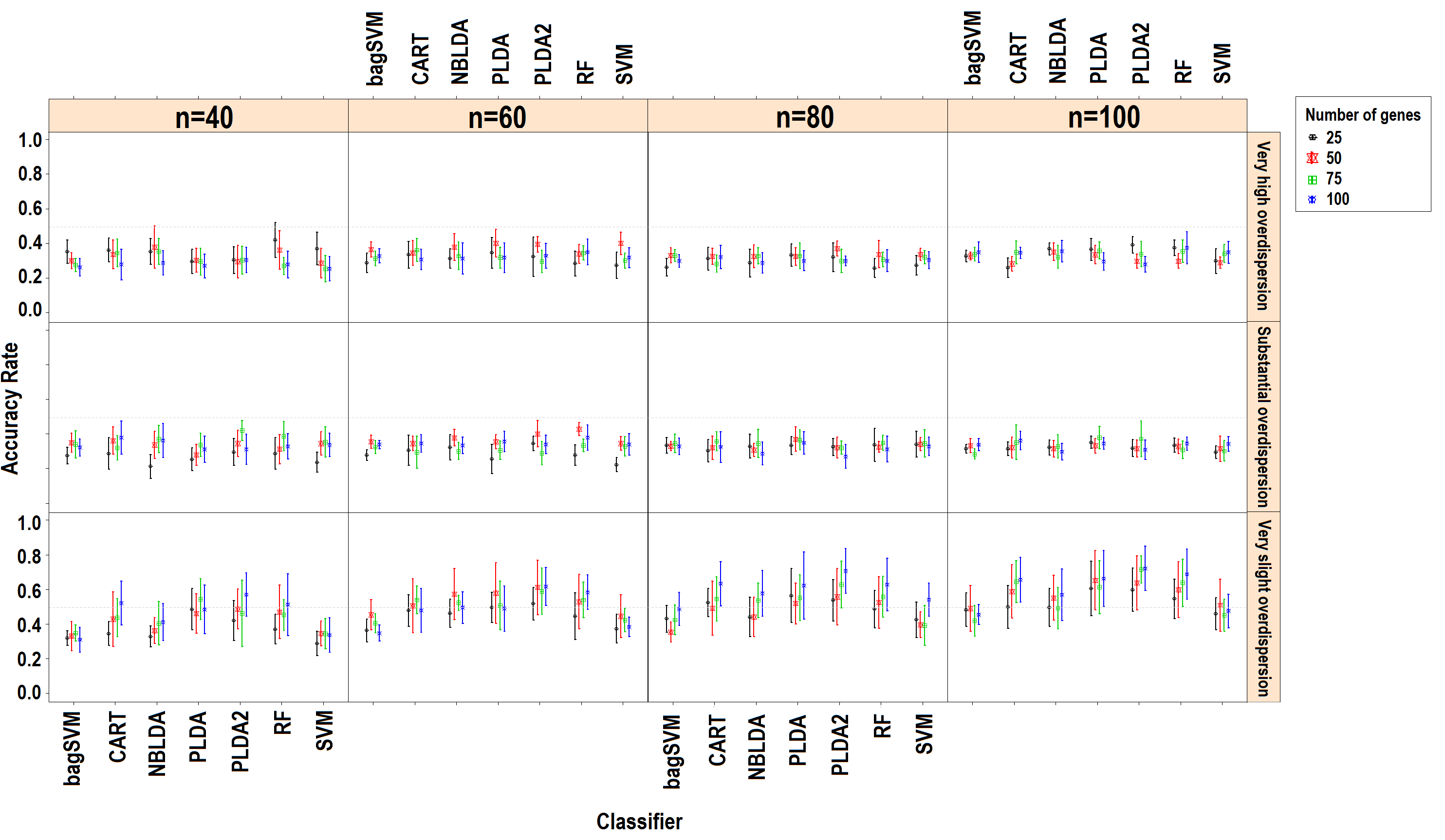

Supplement: S1 File — (ZIP) [file pone.0182507.s001.zip › Additional File 1ΓÇô All figures for simulation results/0.01rlog3.png]

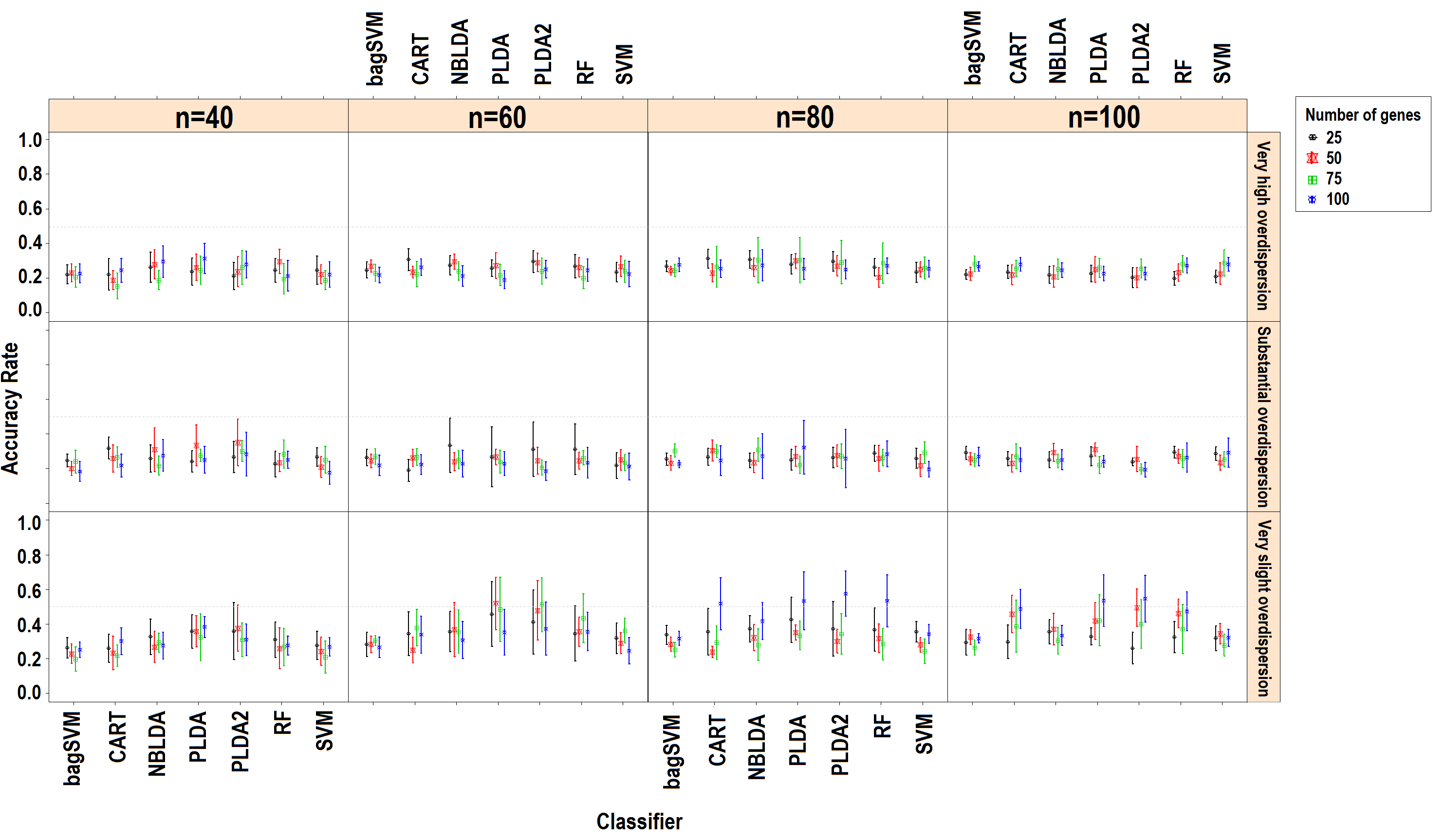

Supplement: S1 File — (ZIP) [file pone.0182507.s001.zip › Additional File 1ΓÇô All figures for simulation results/0.01rlog4.png]

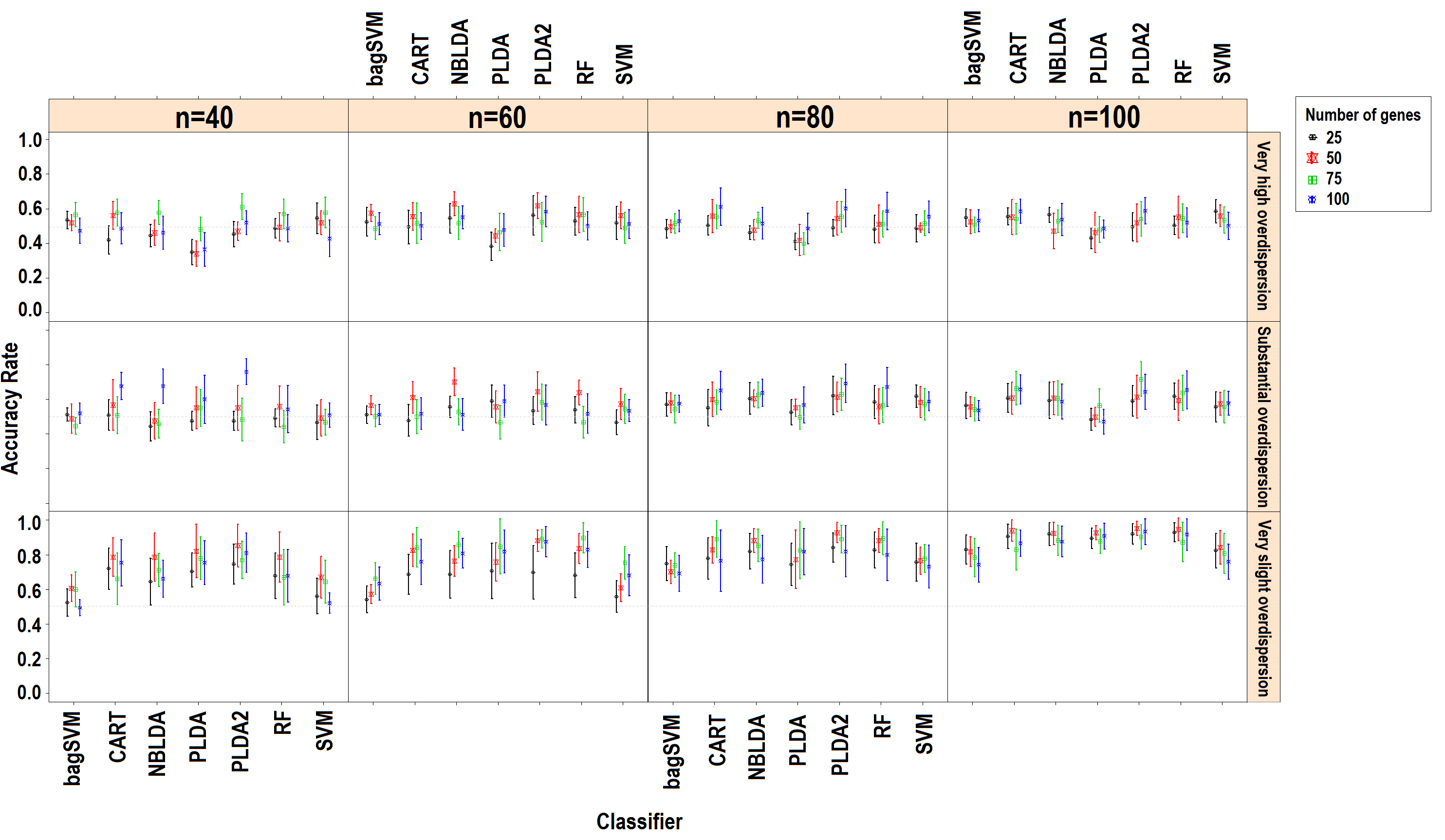

Supplement: S1 File — (ZIP) [file pone.0182507.s001.zip › Additional File 1ΓÇô All figures for simulation results/0.01vst2.png]

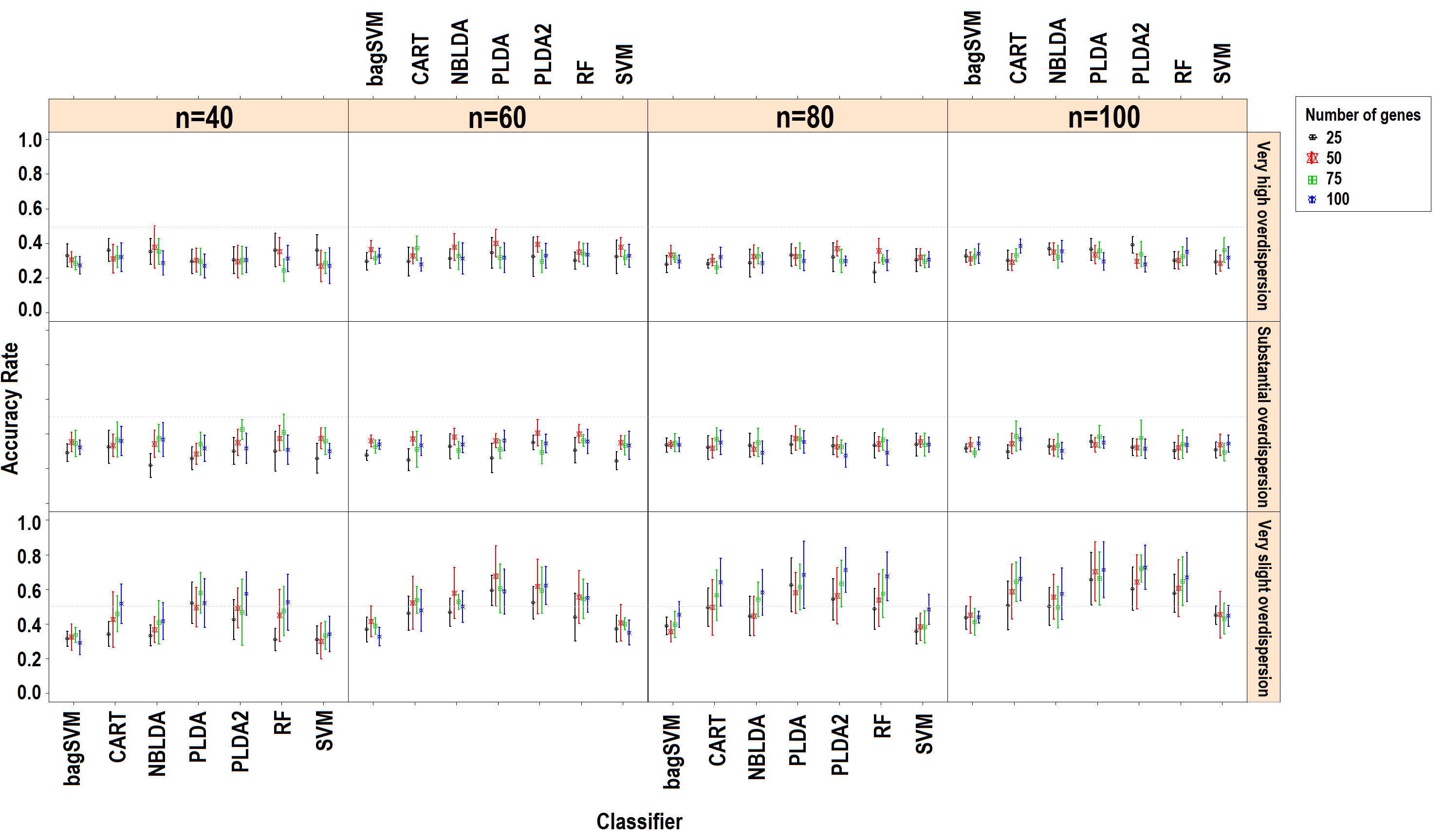

Supplement: S1 File — (ZIP) [file pone.0182507.s001.zip › Additional File 1ΓÇô All figures for simulation results/0.01vst3.png]

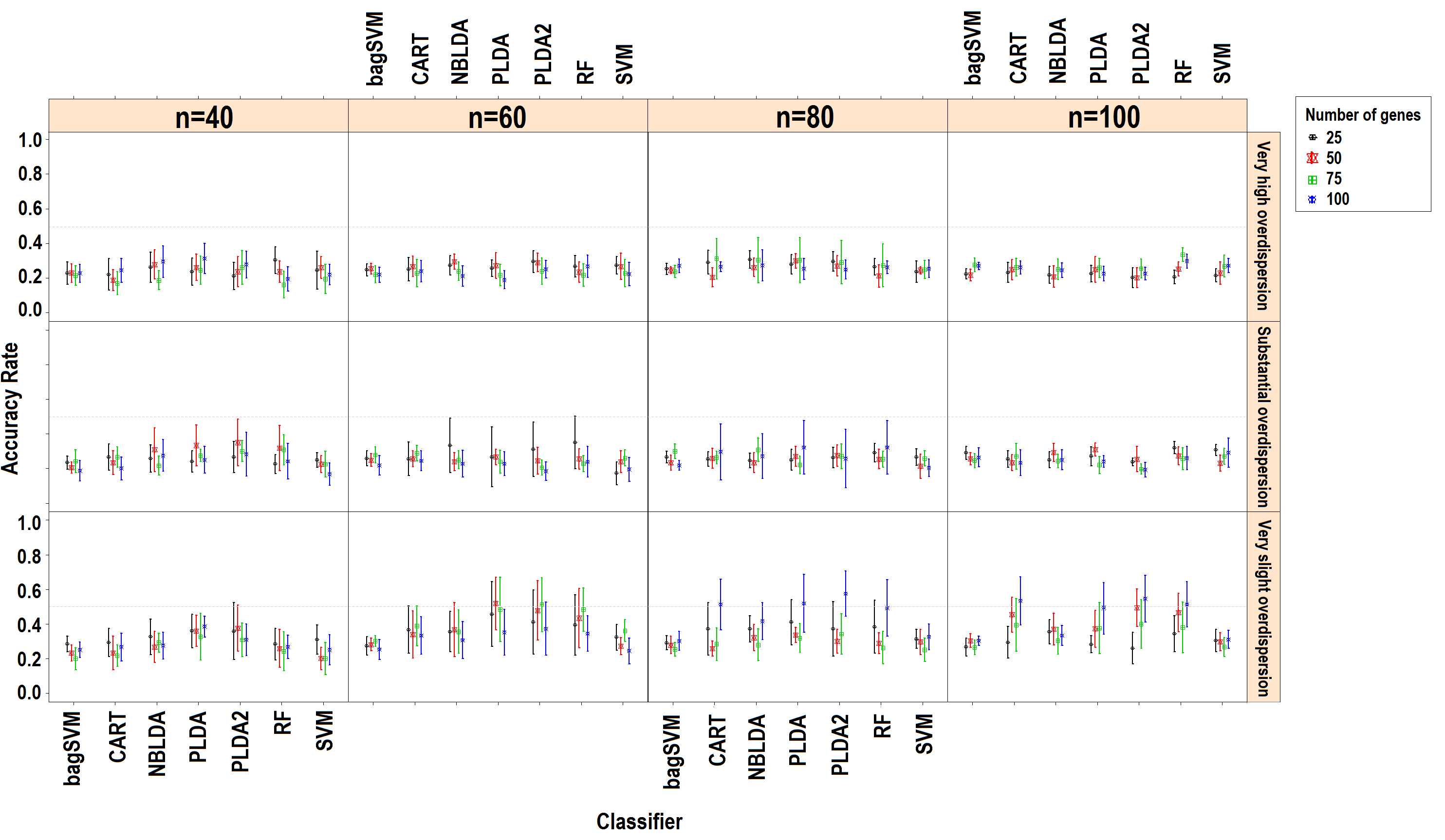

Supplement: S1 File — (ZIP) [file pone.0182507.s001.zip › Additional File 1ΓÇô All figures for simulation results/0.01vst4.png]

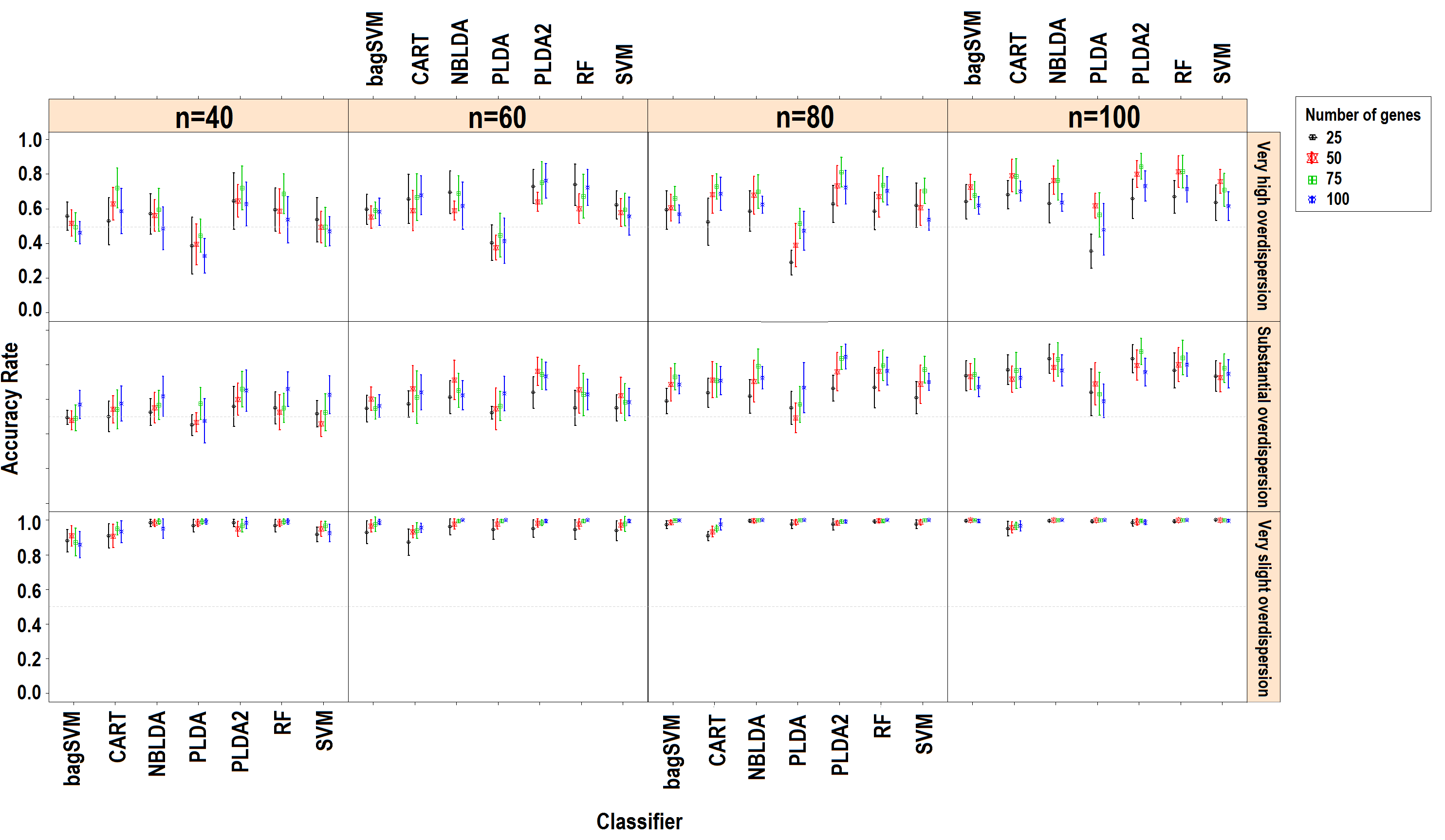

Supplement: S1 File — (ZIP) [file pone.0182507.s001.zip › Additional File 1ΓÇô All figures for simulation results/0.05rlog2.png]

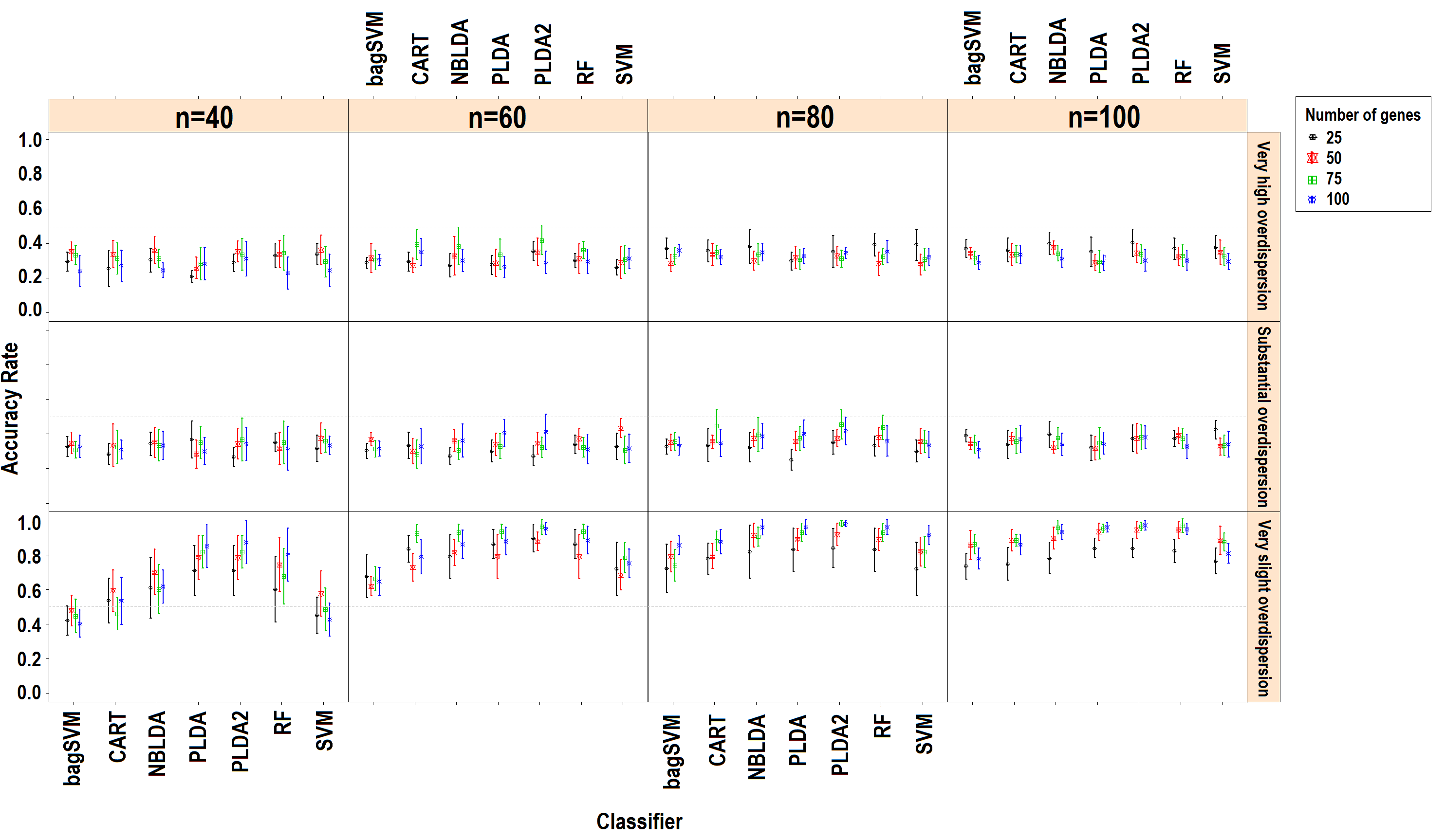

Supplement: S1 File — (ZIP) [file pone.0182507.s001.zip › Additional File 1ΓÇô All figures for simulation results/0.05rlog3.png]

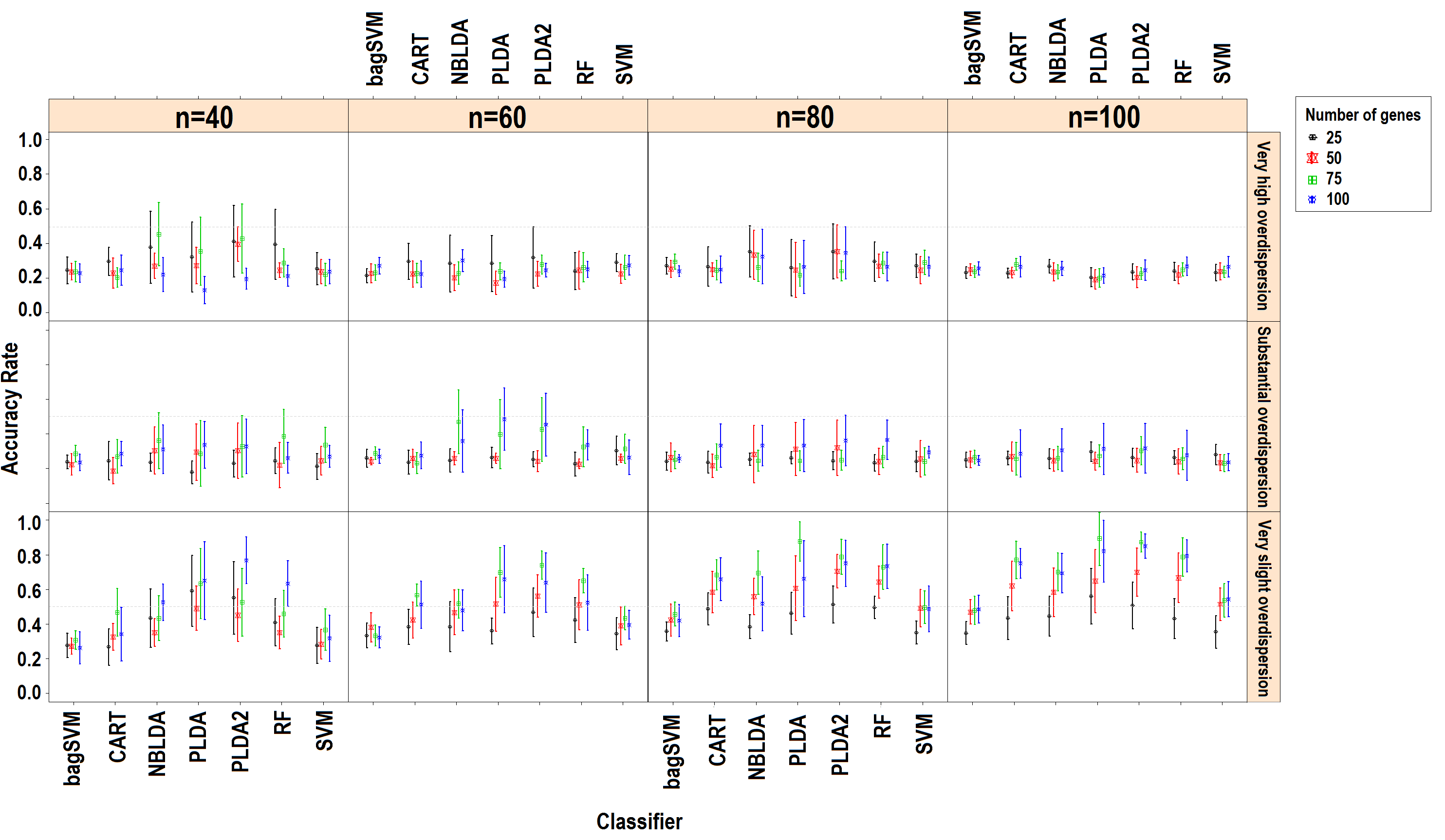

Supplement: S1 File — (ZIP) [file pone.0182507.s001.zip › Additional File 1ΓÇô All figures for simulation results/0.05rlog4.png]

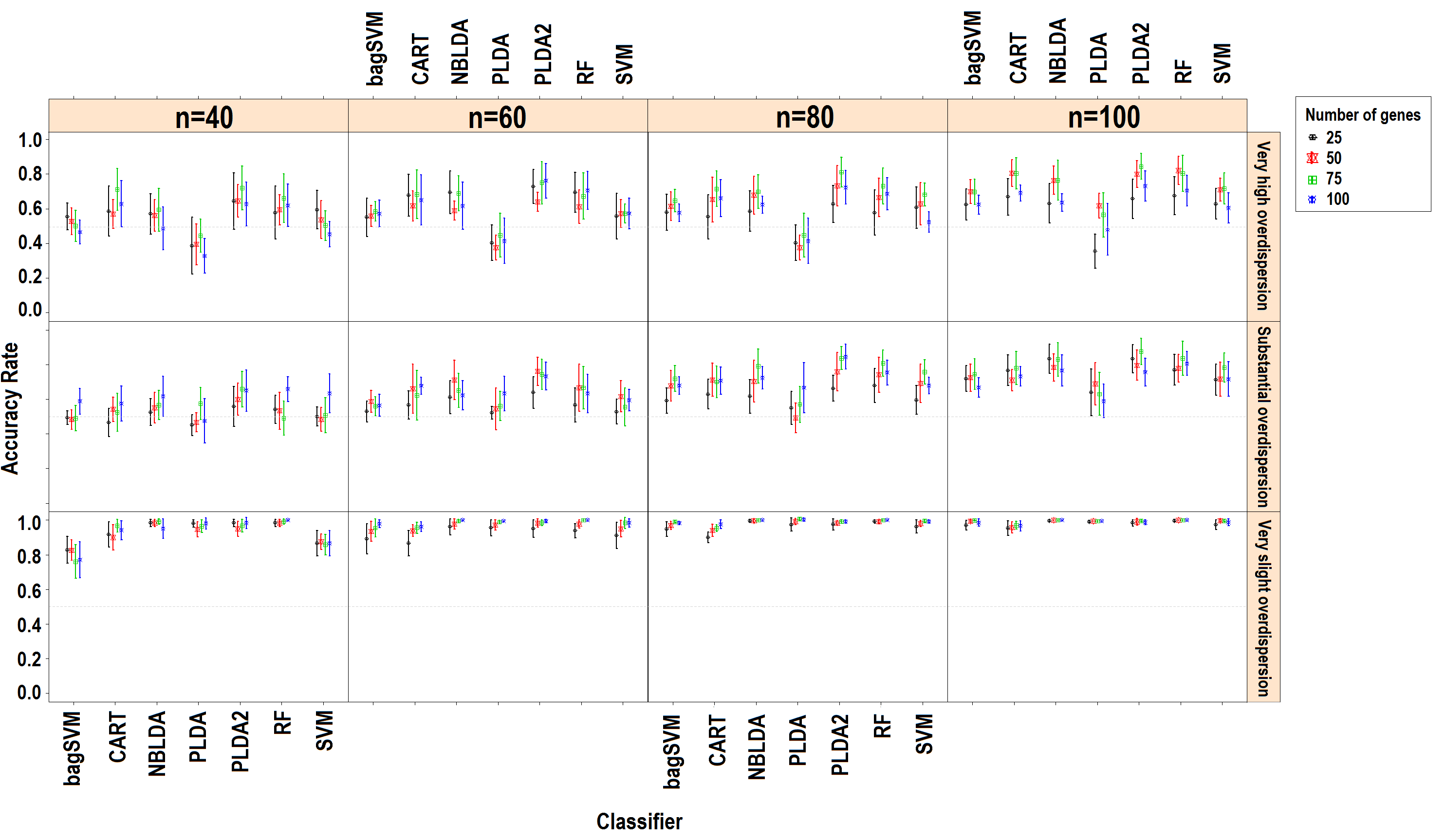

Supplement: S1 File — (ZIP) [file pone.0182507.s001.zip › Additional File 1ΓÇô All figures for simulation results/0.05vst2.png]

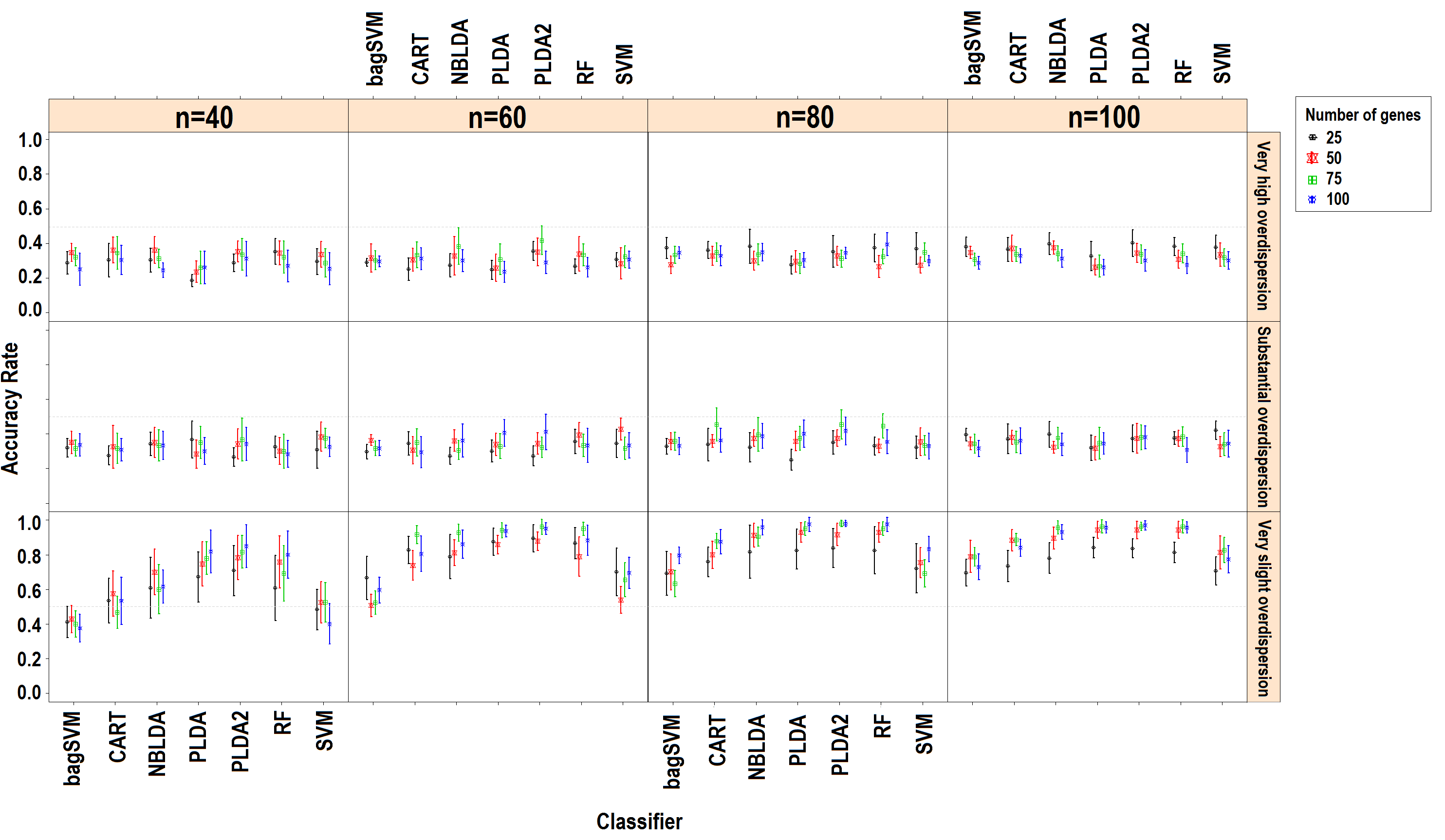

Supplement: S1 File — (ZIP) [file pone.0182507.s001.zip › Additional File 1ΓÇô All figures for simulation results/0.05vst3.png]

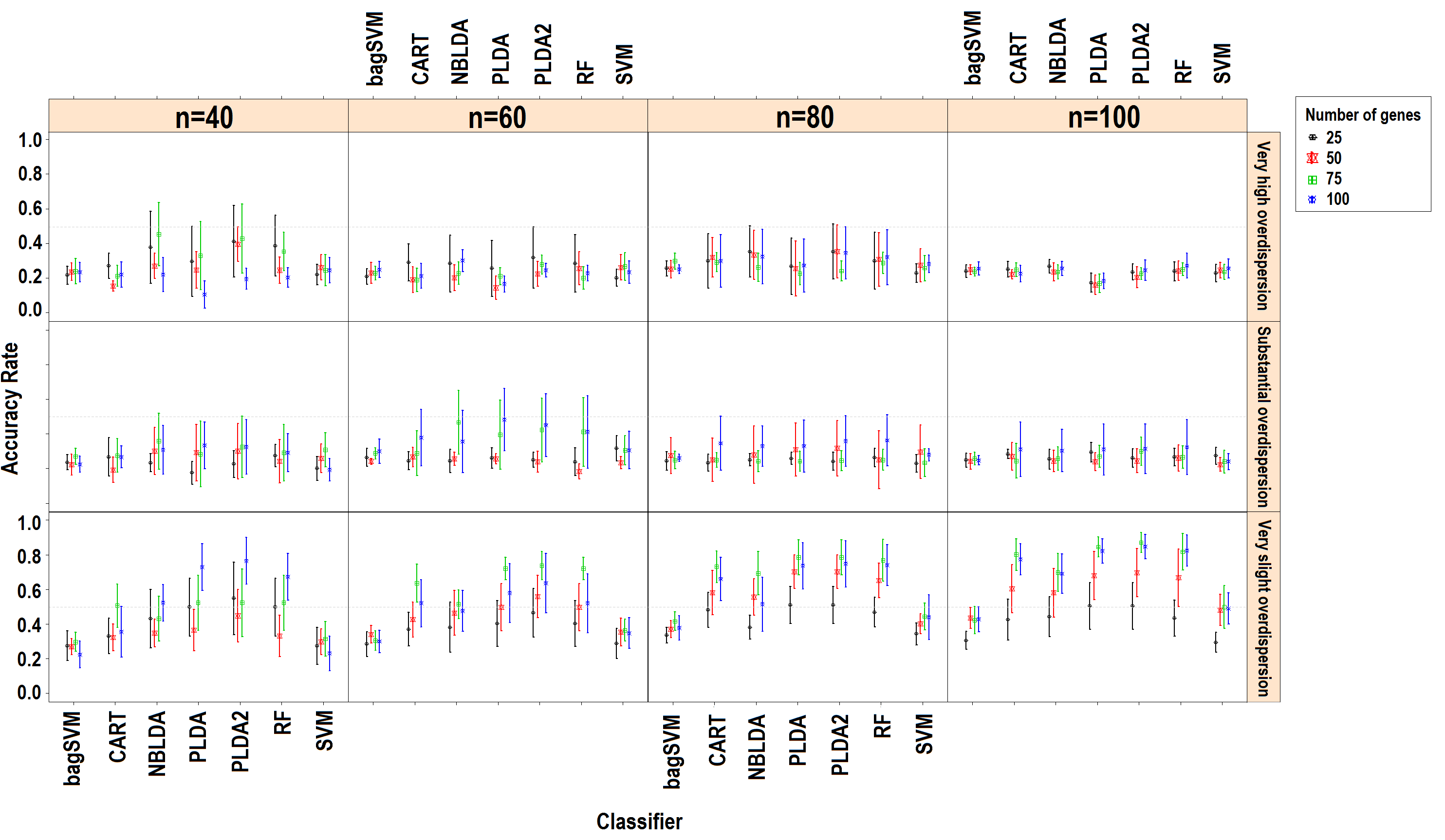

Supplement: S1 File — (ZIP) [file pone.0182507.s001.zip › Additional File 1ΓÇô All figures for simulation results/0.05vst4.png]

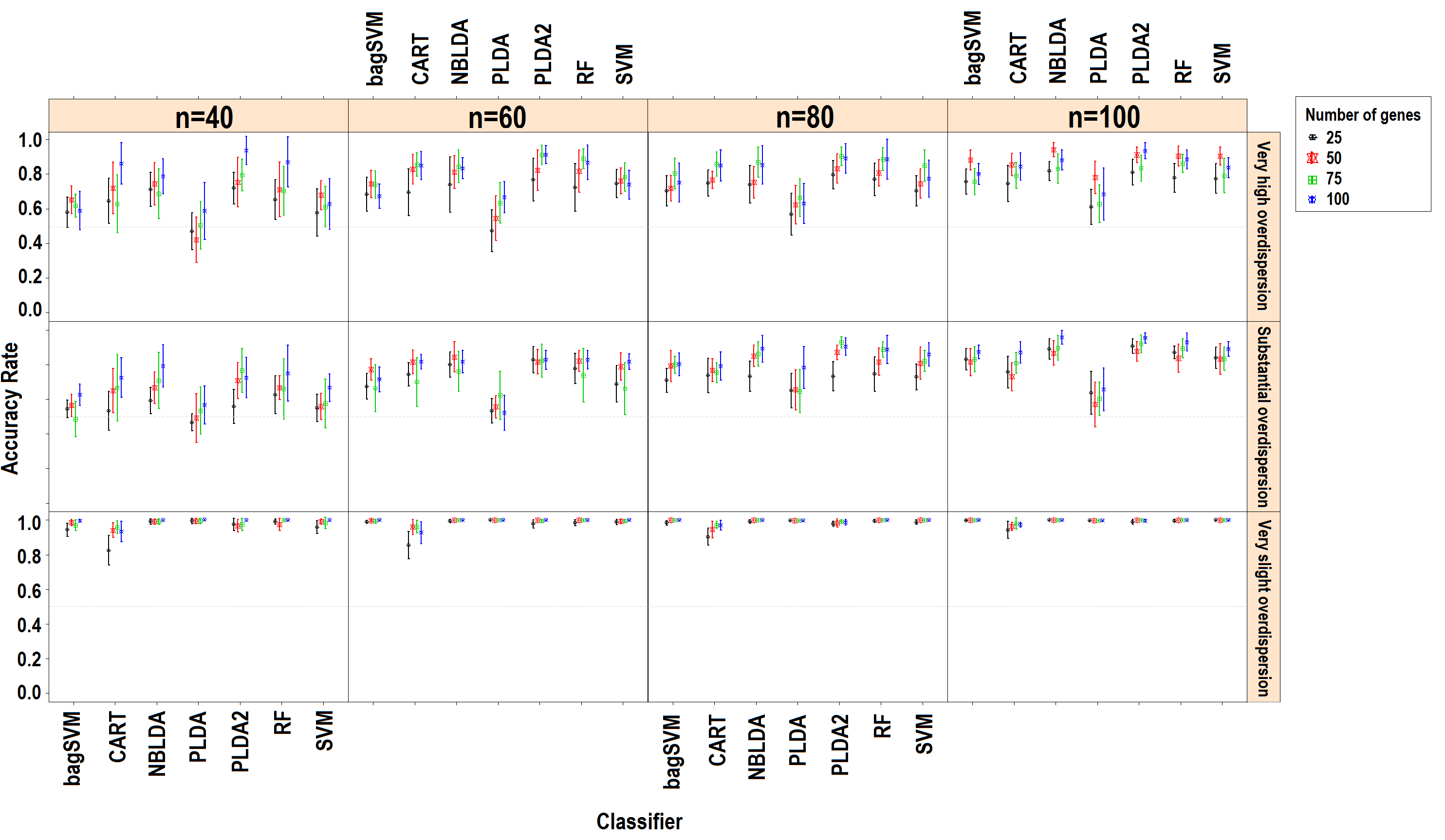

Supplement: S1 File — (ZIP) [file pone.0182507.s001.zip › Additional File 1ΓÇô All figures for simulation results/0.1rlog2.png]

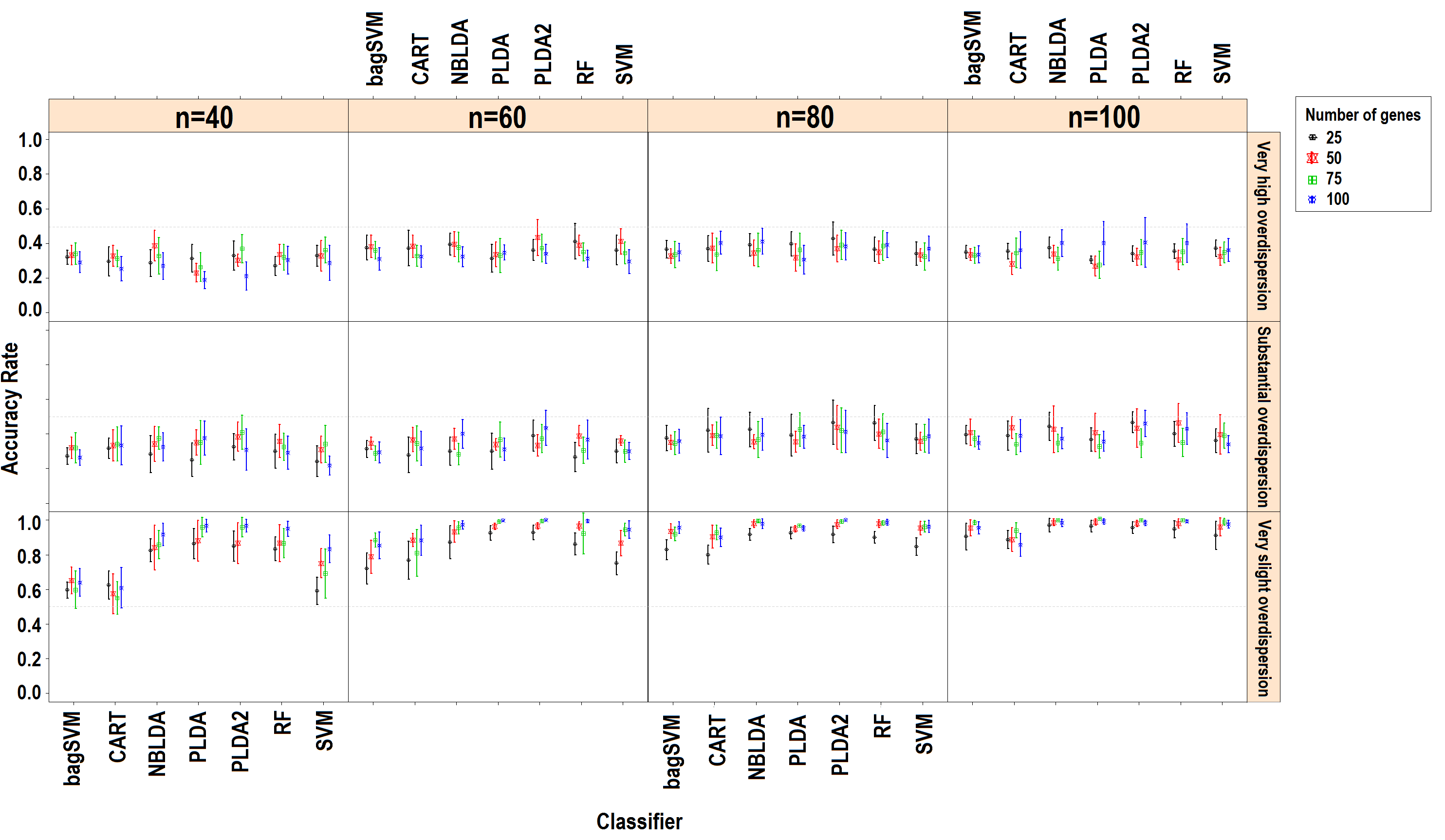

Supplement: S1 File — (ZIP) [file pone.0182507.s001.zip › Additional File 1ΓÇô All figures for simulation results/0.1rlog3.png]

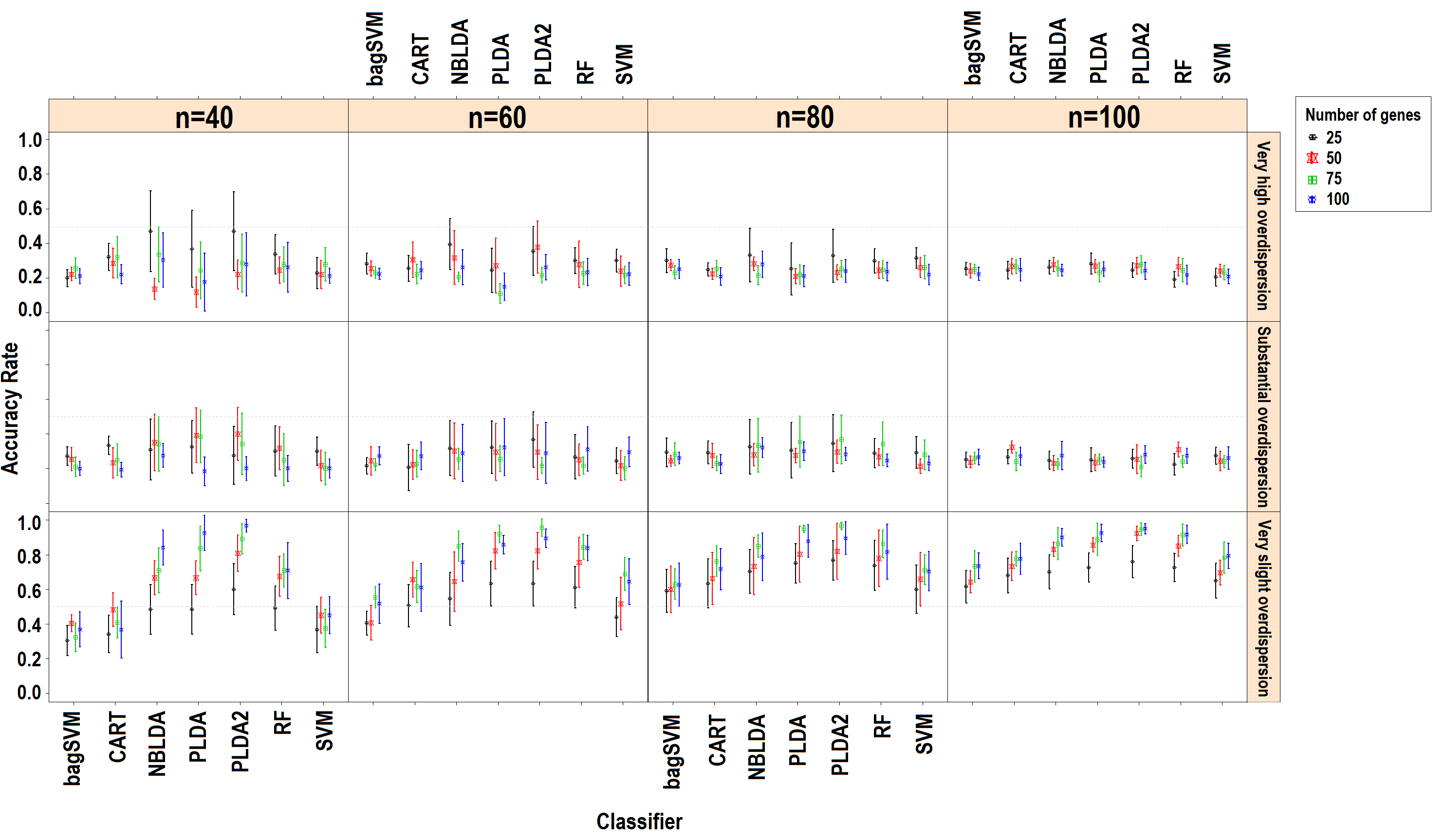

Supplement: S1 File — (ZIP) [file pone.0182507.s001.zip › Additional File 1ΓÇô All figures for simulation results/0.1rlog4.png]

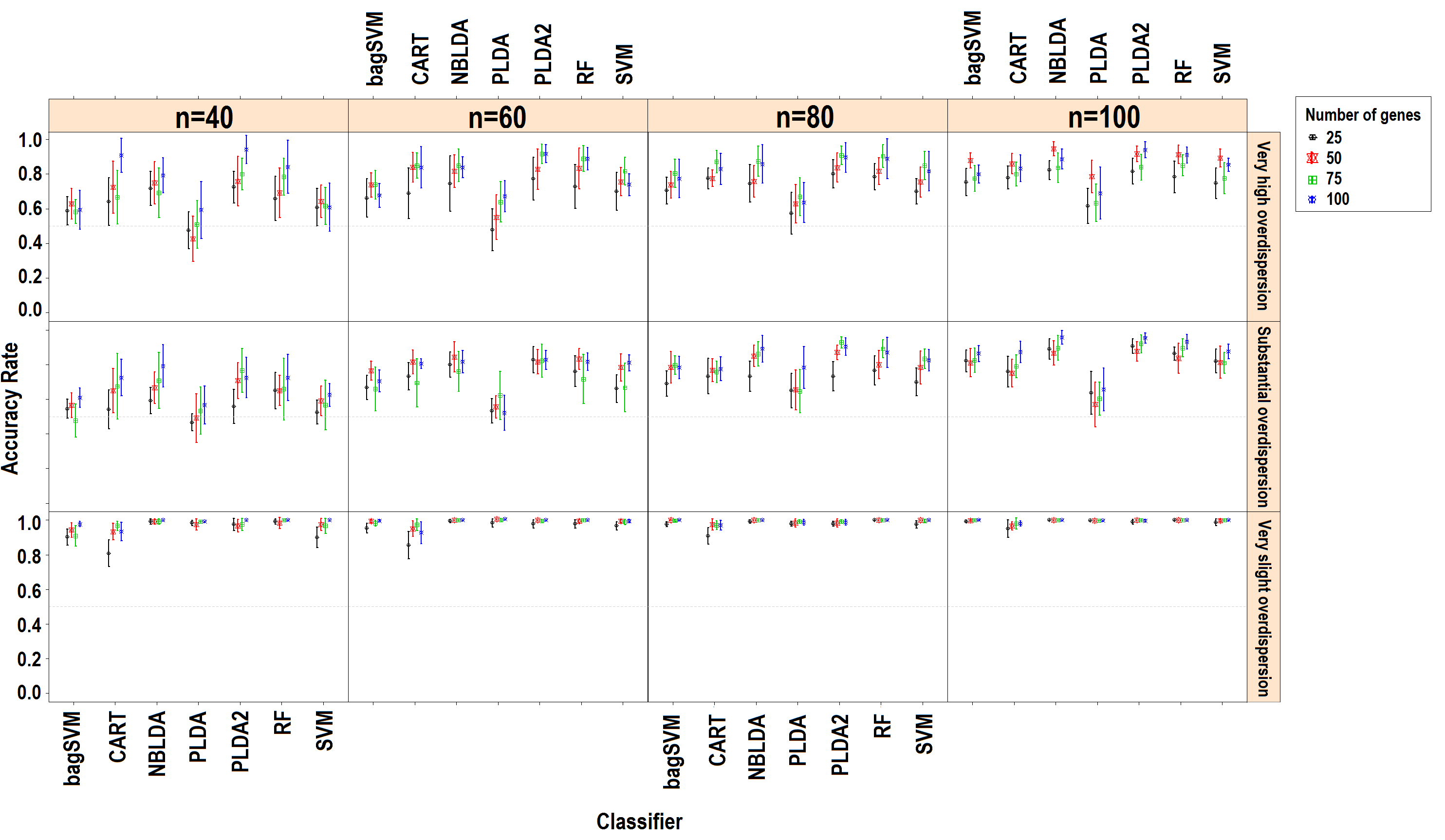

Supplement: S1 File — (ZIP) [file pone.0182507.s001.zip › Additional File 1ΓÇô All figures for simulation results/0.1vst2.png]

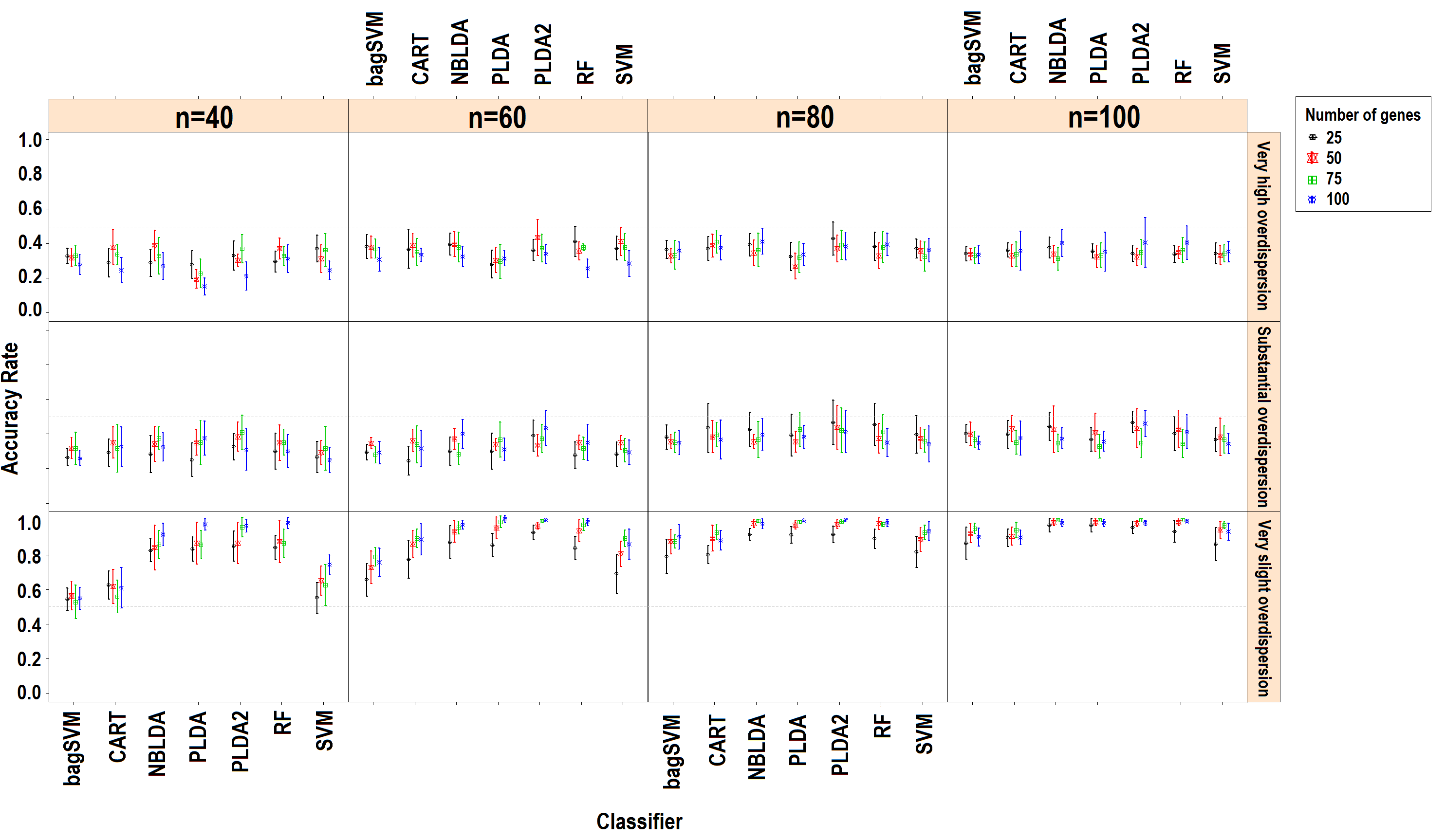

Supplement: S1 File — (ZIP) [file pone.0182507.s001.zip › Additional File 1ΓÇô All figures for simulation results/0.1vst3.png]

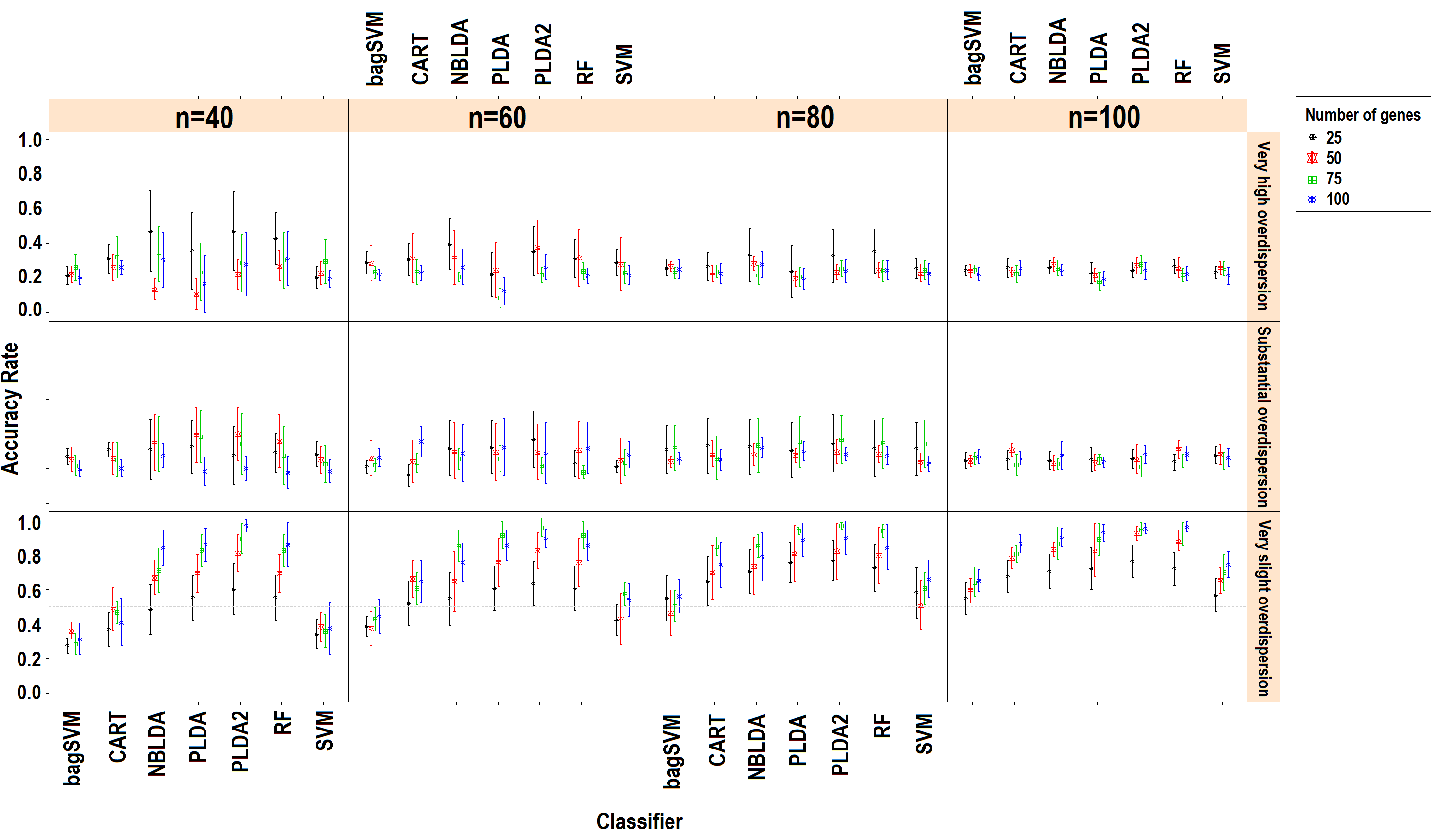

Supplement: S1 File — (ZIP) [file pone.0182507.s001.zip › Additional File 1ΓÇô All figures for simulation results/0.1vst4.png]
