# Supplementary material for: A comprehensive simulation study on classification of RNA-Seq data
Source: PLoS One. 2017 Aug 23;12(8):e0182507. doi: 10.1371/journal.pone.0182507 (PMC5568128; doi:10.1371/journal.pone.0182507)
Supplement: S2 File — (ZIP) [file pone.0182507.s002.zip › MLSeq/inst/doc/MLSeq.pdf]

# MLSeq package: Machine Learning Interface to RNA-Seq Data

Gokmen Zararsiz[1], Dincer Goksuluk[2], Selcuk Korkmaz[2], Vahap Eldem[3], Izzet Parug Duru[4], Turgay Unver[5], Ahmet Ozturk[5]

[1]Erciyes University, Faculty of Medicine, Department of Biostatistics, Kayseri, TURKEY
[2]Hacettepe University, Faculty of Medicine, Department of Biostatistics, Ankara, TURKEY
[3]Istanbul University, Faculty of Science, Department of Biology, Istanbul, TURKEY
[4]Marmara University, Faculty of Science, Department of Physics, Istanbul, TURKEY
[5]Cankiri University, Faculty of Science, Department of Biology, Cankiri, TURKEY
[1]`gokmenzararsiz@erciyes.edu.tr`

**Abstract**

`MLSeq` package provides several algorithms including support vector machines (SVM), bagging support vector machines (bagSVM), random forest (RF) and classification and regression trees (CART) to classify sequencing data. To achieve this, `MLSeq` package requires a count table, which contains the number of reads mapped to each transcript for each sample. This kind of count data can be obtained from RNA-Seq experiments, also from other sequencing experiments such as DNA or ChIP-sequencing. This vignette is presented to guide researchers how to use this package.

## Contents

**1 Introduction** **2**

**2 Preparation of input data** **2**

**3 Data normalization and transformation** **5**

**4 Cross-validation concept** **5**

**5 Building classification models** **5**

**6 Prediction of new samples** **7**

**7 Session info** **8**

# 1 Introduction

With the recent developments in molecular biology, it is feasible to measure the expression levels of thousands of genes simultaneously. Using this information, one major task is the gene-expression based classification. With the use of microarray data, numerous classification algorithms are developed and adapted for this type of classification. RNA-Seq is a recent technology, which uses the capabilities of next-generation sequencing (NGS) technologies. It has some major advantages over microarrays such as providing less noisy data and detecting novel transcripts and isoforms. These advantages can also affect the performance of classification algorithms. Working with less noisy data can improve the predictive performance of classification algorithms. Further, novel transcripts may be a biomarker in related disease or phenotype. `MLSeq` package includes several classification algorithms, also normalization and transformation approaches for RNA-Seq classification. `MLSeq` package can be loaded as below:

```
library(MLSeq)
```

# 2 Preparation of input data

`MLSeq` package expects a count table that contains the number of reads mapped to each transcript for each sample and class label information of samples in an `S4` class `DESeqDataSet` format.

After mapping the RNA-Seq reads to a reference genome or transcriptome, number of reads mapped to the reference genome can be counted to measure transcript abundance. It is very important that the count values must be raw sequencing read counts to implement the methods given in `MLSeq` package. There are a number of functions in Bioconductor packages which summarizes mapped reads to a count data format. These tools include `featureCounts` function in `Rsubread` package [1], `summarizeOverlaps` function in `GenomicRanges` package [2] and `easyRNASeq` package [3]. It is also possible to access this type of count data from Linux-based softwares as `htseq-count` function in `HTSeq` [4] and `multicov` function in `bedtools` [5] software.

In this vignette, we will work with the cervical count data. Cervical data is from an experiment that measures the expressions of 714 miRNA's of human samples [6]. There are 29 tumor and 29 non-tumor cervical samples and these two groups can be treated as two separete classes for classification purpose. We can define the file path with `system.file` function:

```
filepath = system.file("extdata/cervical.txt", package = "MLSeq")
filepath

## [1] "/tmp/RtmpqI4ldU/Rinst3ae1731fb562/MLSeq/extdata/cervical.txt"
```

Next, we can load the data using `read.table` function:

```
cervical = read.table(filepath, header = TRUE)
```

After loading the data, we can check the counts as follows. These counts are the number of mapped miRNA reads to each transcript.

```
head(cervical[, 1:5])
```

```
##          N1   N2   N3    N4   N5
## let-7a   865  810 5505  6692 1456
## let-7a*    3   12   30    73    6
## let-7b   975 2790 4912 24286 1759
## let-7b*   15   18   27   119   11
## let-7c   828 1251 2973  6413  713
## let-7c*    0    0    0     1    0
```

Cervical data is in data.frame format which contains 714 miRNA mapped counts given in rows, belonging to 58 samples given in columns:

```
class(cervical)

## [1] "data.frame"

dim(cervical)

## [1] 714  58
```

First 29 columns of the data contain the miRNA mapped counts of non-tumor samples, while the last 29 columns contain the count information of tumor samples. We need to create a class label information in order to apply classification models.

```
class = data.frame(condition = factor(rep(c("N", "T"), c(29, 29))))
as.factor(class[, 1])

##  [1] N N N N N N N N N N N N N N N N N N N N N N N N N N N N N
## [30] T T T T T T T T T T T T T T T T T T T T T T T T T T T T T
## Levels: N T
```

For simplicity, we can work with a subset of cervical data with first 150 features.

```
data = cervical[c(1:150), ]
```

Now, we can split the data into two parts as training and test sets. Training set can be used to build classification models, and test set can be used to assess the performance of each model. We can use `set.seed` function to specify initial value of random-number seed and use `sample` function for selection.

```
nTest = ceiling(ncol(data) * 0.2)
set.seed(12345)
ind = sample(ncol(data), nTest, FALSE)
```

Now, training and test sets can be created based on this sampling process:

```
data.train = data[, -ind]
data.train = as.matrix(data.train + 1)
classtr = data.frame(condition = class[-ind, ])
```

```
data.test = data[, ind]
data.test = as.matrix(data.test + 1)
classts = data.frame(condition = class[ind, ])
```

Now, we have 46 samples which will be used to train the classification models and have remaining 12 samples to be used to test the model performances:

```
dim(data.train)

## [1] 150  46

dim(data.test)

## [1] 150  12
```

We can now transform our training and test data to `DESeqDataSet` instance, which is the main data structure in the `MLSeq` package. For this purpose, we use the `DESeqDataSetFromMatrix` function of `DESeq2` package [7]:

```
data.trainS4 = DESeqDataSetFromMatrix(countData = data.train, colData = classtr,
    formula(~condition))
data.trainS4 = DESeq(data.trainS4, fitType = "local")
data.trainS4

## class: DESeqDataSet
## dim: 150 46
## exptData(0):
## assays(5): counts mu cooks replaceCounts replaceCooks
## rownames(150): let-7a let-7a* ... miR-18a* miR-18b
## rowData metadata column names(26): baseMean baseVar ...
##   maxCooks replace
## colnames(46): 1 2 ... 45 46
## colData names(3): condition sizeFactor replaceable
```

```
data.testS4 = DESeqDataSetFromMatrix(countData = data.test, colData = classts,
    formula(~condition))
data.testS4 = DESeq(data.testS4, fitType = "local")
data.testS4

## class: DESeqDataSet
## dim: 150 12
## exptData(0):
## assays(3): counts mu cooks
## rownames(150): let-7a let-7a* ... miR-18a* miR-18b
## rowData metadata column names(25): baseMean baseVar ...
##   deviance maxCooks
## colnames(12): 1 2 ... 11 12
## colData names(2): condition sizeFactor
```

Counts and class label information are adequate for classification analysis. However, users can also enter other information. Furthermore, users can directly call the count data obtained from `HTSeq` software using `DESeqDataSetFromHTSeqCount` function of `DESeq2` package.

## 3 Data normalization and transformation

In differential expression analysis of RNA-Seq data, it is crucial to normalize the count data to adjust between-sample differences. In our experiments, we have also seen that normalization significantly increase the performance of most classifiers. In `MLSeq` package two normalization methods are available. First one is the "deseq normalization", which estimates the size factors by dividing each sample by the geometric means of the transcript counts [7]. Median statistic is mostly used as a size factor for each sample. Another normalization method is "trimmed mean of M values (TMM)". TMM first trims the data in both lower and upper side by log-fold changes (default 30%) to minimize the log-fold changes between the samples and by absolute intensity (default 5%). After trimming, TMM calculates a normalization factor using the weighted mean of data. These weights are calculated based on the inverse approximate asymptotic variances using the delta method [8].

After the normalization process, it is useful to transform the data for classification analysis. There are two transformation methods available in `MLSeq` package. First one is the "voom transformation" which applies a logarithmic transformation to normalized count data and computes gene weights using the mean-dispersion relationship [9]. Second transformation method is the "vst transformation". This approach uses an error modeling and the concept of variance stabilizing transformations to estimate the mean-dispersion relationship of data [7].

If the normalization method is selected as "TMM", then `MLSeq` package automatically applies "voom" transformation. However, it is possible to select either "vst" or "voom" transformations after "deseq" normalization.

Further details on these normalization and transformation methods can be found in referenced papers.

## 4 Cross-validation concept

One essential goal of classification analysis is to build a generalizable model that will have a low misclassification error when applied to new samples. One way is to use $k$-fold cross-validation to validate obtained model. $k$-fold cross-validation technique randomly splits the data into $k$ non-overlapping and equally sized subsets. A classification model is trained on $(k-1)$ subsets and tested in the remaining subsets. This process is repeated $k$ times, thus all subsets are used as a test set in each step. `MLSeq` package also has the repeat option to obtain more generalizable models. Giving a number of $m$ repeats, cross validation concept is applied $m$ times.

## 5 Building classification models

Now, we can train our data, data.trainS4, using one of the classifiers among SVM, bagSVM, RF and CART algorithms. To build a classification model, we simply use the `classify` function. First, let us use SVM classifier and choose "deseq" as normalization method, "vst" as transformation method and assign this model in "svm" object. We also define the number of cross validation fold as "cv=5" and number of repeats as "rpt=3" for model validation. The reference class is considered as "T" via `ref = "T"`.

```
svm = classify(data = data.trainS4, method = "svm", normalize = "deseq",
    deseqTransform = "vst", cv = 5, rpt = 3, ref = "T")
svm

##
##   An object of class  MLSeq
##
##             Method  :  svm
##
##        Accuracy(%)  :  97.83
##     Sensitivity(%)  :  100
##     Specificity(%)  :  95.65
##
##   Reference Class   :  T
```

After running the code given above, we obtain the results in MLSeq class. SVM successfully fits a model with 97.8% true classification accuracy by misclassifying only one non-tumor sample.

"svm" object also stores information about model training and the parameters used to build this model.

```
getSlots("MLSeq")

##             method    deseqTransform     normalization
##        "character"       "character"       "character"
##       confusionMat           trained               ref
## "confusionMatrix"           "train"       "character"

trained(svm)

## Support Vector Machines with Radial Basis Function Kernel
##
##  46 samples
## 150 predictors
##   2 classes: 'T', 'N'
##
## No pre-processing
## Resampling: Cross-Validated (5 fold, repeated 3 times)
##
## Summary of sample sizes: 36, 37, 37, 36, 38, 36, ...
##
## Resampling results across tuning parameters:
##
##   C    Accuracy  Kappa  Accuracy SD  Kappa SD
##   0.2  0.9       0.7    0.1          0.2
##   0.5  0.9       0.8    0.09         0.2
##   1    0.9       0.8    0.09         0.2
##
## Tuning parameter 'sigma' was held constant at a value
##  of 0.004331
```

```
## Accuracy was used to select the optimal model using
##  the largest value.
## The final values used for the model were sigma = 0.004 and C
##  = 1.
```

Now, let us train another model with same parameters using RF classifier and save this model in "rf" object.

```
rf = classify(data = data.trainS4, method = "randomforest", normalize = "deseq",
    deseqTransform = "vst", cv = 5, rpt = 3, ref = "T")
rf

##
##   An object of class  MLSeq
##
##             Method  :  randomforest
##
##        Accuracy(%)  :  100
##     Sensitivity(%)  :  100
##     Specificity(%)  :  100
##
##   Reference Class   :  T
```

We can see that RF method successfully trained the model without misclassifying any samples.

# 6 Prediction of new samples

Now, we will predict the class labels of our test data "data.testS4" and test the performance of classifiers based on the models we built using `classify` function. Here, we use `predictClassify` function in order to achieve this goal.

```
pred.svm = predictClassify(svm, data.testS4)
pred.svm

##  [1] T T T T T T N N T T N N
## Levels: T N
```

```
pred.rf = predictClassify(rf, data.testS4)
pred.rf

##  [1] T N T T N T N N T T N N
## Levels: T N
```

To assess the predictive performance of each method, we can cross the actual class labels and predictions in a table :

```
table(pred.svm, relevel(data.testS4$condition, 2))
```

```
##
## pred.svm T N
##        T 6 2
##        N 0 4

table(pred.rf, relevel(data.testS4$condition, 2))

##
## pred.rf T N
##       T 5 1
##       N 1 5
```

We can see that SVM and RF showed similar predictive performances and both methods correctly classified 10 out of 12 test samples with 83.3% classification accuracy. However, note that the true classification rate for `predictClassify` is dependent on number of repeats and selected folds. Therefore, users may have different results that we have obtained above.

# 7 Session info

```
sessionInfo()

## R version 3.1.0 alpha (2014-03-12 r65175)
## Platform: x86_64-unknown-linux-gnu (64-bit)
##
## locale:
##  [1] LC_CTYPE=en_US.UTF-8       LC_NUMERIC=C
##  [3] LC_TIME=en_US.UTF-8        LC_COLLATE=C
##  [5] LC_MONETARY=en_US.UTF-8    LC_MESSAGES=en_US.UTF-8
##  [7] LC_PAPER=en_US.UTF-8       LC_NAME=C
##  [9] LC_ADDRESS=C               LC_TELEPHONE=C
## [11] LC_MEASUREMENT=en_US.UTF-8 LC_IDENTIFICATION=C
##
## attached base packages:
## [1] parallel  stats     graphics  grDevices utils     datasets
## [7] methods   base
##
## other attached packages:
##  [1] e1071_1.6-3               MLSeq_0.99.3
##  [3] edgeR_3.5.27              limma_3.19.28
##  [5] kernlab_0.9-19            randomForest_4.6-7
##  [7] Biobase_2.23.6            DESeq2_1.3.58
##  [9] RcppArmadillo_0.4.100.2.1 Rcpp_0.11.1
## [11] GenomicRanges_1.15.40     GenomeInfoDb_0.99.23
## [13] IRanges_1.21.36           BiocGenerics_0.9.3
## [15] caret_6.0-24              ggplot2_0.9.3.1
## [17] lattice_0.20-27           knitr_1.5
##
## loaded via a namespace (and not attached):
```

```
##  [1] AnnotationDbi_1.25.16 DBI_0.2-7
##  [3] MASS_7.3-30           RColorBrewer_1.0-5
##  [5] RSQLite_0.11.4        XML_3.98-1.1
##  [7] XVector_0.3.7         annotate_1.41.2
##  [9] car_2.0-19            class_7.3-9
## [11] codetools_0.2-8       colorspace_1.2-4
## [13] compiler_3.1.0        dichromat_2.0-0
## [15] digest_0.6.4          evaluate_0.5.1
## [17] foreach_1.4.1         formatR_0.10
## [19] genefilter_1.45.2     geneplotter_1.41.1
## [21] grid_3.1.0            gtable_0.1.2
## [23] highr_0.3             iterators_1.0.6
## [25] labeling_0.2          locfit_1.5-9.1
## [27] munsell_0.4.2         nnet_7.3-7
## [29] plyr_1.8.1            proto_0.3-10
## [31] reshape2_1.2.2        scales_0.2.3
## [33] splines_3.1.0         stats4_3.1.0
## [35] stringr_0.6.2         survival_2.37-7
## [37] tools_3.1.0           xtable_1.7-3
```

# References


[1] Liao Y, Smyth GK, Shi W (2013). featureCounts: an efficient general purpose program for assigning sequence reads to genomic features. *Bioinformatics*.

[2] Lawrence M, Huber W, Pages H, et al. (2013). Software for Computing and Annotating Genomic Ranges. *Plos Computational Biology*, DOI: 10.1371/journal.pcbi.1003118.

[3] Delhomme N, Padioleau I, Furlong EE, et al. (2012). easyRNASeq: a bioconductor package for processing RNA-Seq data. *Bioinformatics*, 28(**19**):2532-2533.

[4] http://www-huber.embl.de/users/anders/HTSeq/doc/overview.html

[5] Quinlan AR, Hall IM (2010). BEDTools: a flexible suite of utilities for comparing genomic features. *Bioinformatics*, 26(**6**):841-842.

[6] Witten D, Tibshirani R, Gu S, et al. (2010). Ultra-high throughput sequencing-based small RNA discovery an discrete statistical biomarker analysis in a collection of cervical tumors and matched controls. *BMC Biology*, 8(**58**).

[7] Anders S, Huber W (2010). Differential expression analysis for sequence count data. *Genome Biology*, 11(**10**):R106.

[8] Robinson MD, Oshlack A (2010). A scaling normalization method for differential expression analysis of RNA-Seq data. *Genome Biology*, 11:R25, doi:10.1186/gb–2010–11–3–r25.

[9] Charity WL, Chen Y, Shi W, et al. (2014) Voom: precision weights unlock linear model analysis tools for RNA-Seq read counts, *Genome Biology*, 15:R29, doi:10.1186/gb–2014–15–2–r29.
