# Supplementary material for: A comprehensive simulation study on classification of RNA-Seq data
Source: PLoS One. 2017 Aug 23;12(8):e0182507. doi: 10.1371/journal.pone.0182507 (PMC5568128; doi:10.1371/journal.pone.0182507)
Supplement: S3 File — (ZIP) [file pone.0182507.s003.zip › R Codes/PDFs/BreimanBaggingPredictors.pdf]

Machine Learning, 24, 123–140 (1996)
© 1996 Kluwer Academic Publishers, Boston. Manufactured in The Netherlands.

Bagging Predictors

LEO BREIMAN leo@stat.berkeley.edu
Statistics Department, University of California, Berkeley, CA 94720

Editor: Ross Quinlan

Abstract. Bagging predictors is a method for generating multiple versions of a predictor and using these to get an aggregated predictor. The aggregation averages over the versions when predicting a numerical outcome and does a plurality vote when predicting a class. The multiple versions are formed by making bootstrap replicates of the learning set and using these as new learning sets. Tests on real and simulated data sets using classification and regression trees and subset selection in linear regression show that bagging can give substantial gains in accuracy. The vital element is the instability of the prediction method. If perturbing the learning set can cause significant changes in the predictor constructed, then bagging can improve accuracy.

Keywords: Aggregation, Bootstrap, Averaging, Combining

1. Introduction

A learning set of $\mathcal{L}$ consists of data $\{(y_n, \boldsymbol{x}_n), n = 1, \ldots, N\}$ where the y's are either class labels or a numerical response. Assume we have a procedure for using this learning set to form a predictor $\varphi(\boldsymbol{x}, \mathcal{L})$ — if the input is $\boldsymbol{x}$ we predict y by $\varphi(\boldsymbol{x}, \mathcal{L})$. Now, suppose we are given a sequence of learnings sets $\{\mathcal{L}_k\}$ each consisting of N independent observations from the same underlying distribution as $\mathcal{L}$. Our mission is to use the $\{\mathcal{L}_k\}$ to get a better predictor than the single learning set predictor $\varphi(\boldsymbol{x}, \mathcal{L})$. The restriction is that all we are allowed to work with is the sequence of predictors $\{\varphi(\boldsymbol{x}, \mathcal{L}_k)\}$.

If y is numerical, an obvious procedure is to replace $\varphi(\boldsymbol{x}, \mathcal{L})$ by the average of $\varphi(\boldsymbol{x}, \mathcal{L}_k)$ over k, i.e. by $\varphi_A(\boldsymbol{x}) = E_{\mathcal{L}}\varphi(\boldsymbol{x}, \mathcal{L})$ where $E_{\mathcal{L}}$ denotes the expectation over $\mathcal{L}$, and the subscript A in φ_A denotes aggregation. If $\varphi(\boldsymbol{x}, \mathcal{L})$ predicts a class $j \in \{1, \ldots, J\}$, then one method of aggregating the $\varphi(\boldsymbol{x}, \mathcal{L}_k)$ is by voting. Let $N_j = nr\{k; \varphi(\boldsymbol{x}, \mathcal{L}_k) = j\}$ and take $\varphi_A(\boldsymbol{x}) = \text{argmax}_j N_j$, that is, the j for which N_j is maximum.

Usually, though, we have a single learning set $\mathcal{L}$ without the luxury of replicates of $\mathcal{L}$. Still, an imitation of the process leading to φ_A can be done. Take repeated bootstrap samples $\{\mathcal{L}^{(B)}\}$ from $\mathcal{L}$, and form $\{\varphi(\boldsymbol{x}, \mathcal{L}^{(B)})\}$. If y is numerical, take φ_B as

$$\varphi_B(\boldsymbol{x}) = av_B\varphi(\boldsymbol{x}, \mathcal{L}^{(B)}).$$

If y is a class label, let the $\{\varphi(\boldsymbol{x}, \mathcal{L}^{(B)})\}$ vote to form $\varphi_B(\boldsymbol{x})$. We call this procedure "**b**ootstrap **agg**regat**ing**" and use the acronym **bagging**.

The $\{\mathcal{L}^{(B)}\}$ form replicate data sets, each consisting of N cases, drawn at random, *but with replacement*, from $\mathcal{L}$. Each $(y_n, \boldsymbol{x}_n)$ may appear repeated times or not at all in any particular $\mathcal{L}^{(B)}$. The $\{\mathcal{L}^{(B)}\}$ are replicate data sets drawn from the bootstrap distribution approximating the distribution underlying $\mathcal{L}$. For background on bootstrapping, see Efron

and Tibshirani [1993]. A critical factor in whether bagging will improve accuracy is the stability of the procedure for constructing φ. If changes in $\mathcal{L}$, i.e. a replicate $\mathcal{L}$, produces small changes in φ, then φ_B will be close to φ. Improvement will occur for unstable procedures where a small change in $\mathcal{L}$ can result in large changes in φ. Instability was studied in Breiman [1994] where it was pointed out that neural nets, classification and regression trees, and subset selection in linear regression were unstable, while k-nearest neighbor methods were stable.

For unstable procedures bagging works well. In Section 2 we bag classification trees on a variety of data sets. The reduction in test set misclassification rates ranges from 6% to 77%. In Section 3 regression trees are bagged with reduction in test set mean squared error on data sets ranging from 21% to 46%. Section 4 goes over some theoretical justification for bagging and attempts to understand when it will or will not work well. This is illustrated by the results of Section 5 on subset selection in linear regression using simulated data. Section 6 gives concluding remarks. These discuss how many bootstrap replications are useful, bagging nearest neighbor classifiers and bagging class probability estimates. The Appendix gives brief descriptions of the data sets.

The evidence, both experimental and theoretical, is that bagging can push a good but unstable procedure a significant step towards optimality. On the other hand, it can slightly degrade the performance of stable procedures. There has been recent work in the literature with some of the flavor of bagging. In particular, there has been some work on averaging and voting over multiple trees. Buntine [1991] gave a Bayesian approach, Kwok and Carter [1990] used voting over multiple trees generated by using alternative splits, and Heath et al. [1993] used voting over multiple trees generated by alternative oblique splits. Dietterich and Bakiri [1991] showed that a method for coding many class problems into a large number of two class problems increases accuracy. There is some commonality of this idea with bagging.

2. Bagging Classification Trees

2.1. *Results for Moderate Sized Data Sets*

Bagging was applied to classification trees using the following data sets

waveform (simulated)
heart
breast cancer (Wisconsin)
ionosphere
diabetes
glass
soybean

All of these except the heart data are in the UCI repository (ftp to ics.uci.edu/pub/machine-learning-databases). Table 1 gives a numerical summary of the data sets and brief descriptions are contained in the Appendix:

Table 1. Data Set Summary

Data Set	# Samples	# Variables	# Classes
waveform	300	21	3
heart	1395	16	2
breast cancer	699	9	2
ionosphere	351	34	2
diabetes	768	8	2
glass	214	9	6
soybean	683	35	19

In all runs the following procedure was used:

i) The data set is randomly divided into a test set $\mathcal{T}$ and a learning set $\mathcal{L}$. In the real data sets $\mathcal{T}$ is 10% of the data. In the simulated waveform data, 1800 samples are generated. $\mathcal{L}$ consists of 300 of these, and $\mathcal{T}$ the remainder.

ii) A classification tree is constructed from $\mathcal{L}$ using 10-fold cross-validation. Running the test set $\mathcal{T}$ down this tree gives the misclassification rate $e_S(\mathcal{L}, \mathcal{T})$.

iii) A bootstrap sample $\mathcal{L}_B$ is selected from $\mathcal{L}$, and a tree grown using $\mathcal{L}_B$. The original learning set $\mathcal{L}$ is used as test set to select the best pruned subtree (see Section 4.3). This is repeated 50 times giving tree classifiers $\phi_1(\boldsymbol{x}), \ldots, \phi_{50}(\boldsymbol{x})$.

iv) If $(j_n, \boldsymbol{x}_n) \in \mathcal{T}$, then the estimated class of $\boldsymbol{x}_n$ is that class having the plurality in $\phi_1(\boldsymbol{x}_n), \ldots, \phi_{50}(\boldsymbol{x}_n)$. If there is a tie, the estimated class is the one with the lowest class label. The proportion of times the estimated class differs from the true class is the bagging misclassification rate $e_B(\mathcal{L}, \mathcal{T})$.

v) The random division of the data into $\mathcal{L}$ and $\mathcal{T}$ is repeated 100 times and the reported $\bar{e}_S$, $\bar{e}_B$ are the averages over the 100 iterations. For the waveform data, 1800 new cases are generated at each iteration. Standard errors of $\bar{e}_S$ and $\bar{e}_B$ over the 100 iterations are also computed.

Table 2 gives the values of $\bar{e}_S$, $\bar{e}_B$, and Table 3 their estimated standard errors.

Table 2. Misclassification Rates (%)

Data Set	$\bar{e}_S$	$\bar{e}_B$	Decrease
waveform	29.1	19.3	34%
heart	4.9	2.8	43%
breast cancer	5.9	3.7	37%
ionosphere	11.2	7.9	29%
diabetes	25.3	23.9	6%
glass	30.4	23.6	22%
soybean	8.6	6.8	21%

Table 3. Standard Errors of Misclassification

Data Set	$SE(\bar{e}_S)$	$SE(\bar{e}_B)$
waveform	.2	.1
heart	.2	.1
breast cancer	.3	.2
ionosphere	.5	.4
diabetes	.4	.4
glass	1.1	.9
soybean	.4	.3

For the waveform data, its known that the lowest possible error rate is 14%. Bagging reduces the excess error by about two-thirds. We conjecture that the small decrease in the diabetes data set is because bagging is pushing close to the minimal attainable error rate. For instance, in the comparison by Michie et al. [1994] of 22 classifiers on this data set, the smallest error rate achieved (estimated by 12-fold cross-validation) was 22.3%.

2.2. *Statlog Comparisons for Larger Data Sets*

The Statlog Project [Michie et al., 1994] compared 22 classification methods over a wide variety of data sets. For most of these data sets, error rates were estimated using a single cross-validation. Without knowing the random subdivisions used in these cross-validations, the variability in the resulting error estimates makes comparisons chancey.

However, there were larger data sets used in the project which were divided into training and test sets. Four are publically available, and we used these as a basis for comparison. They can be accessed by ftp to ftp.strath.ac.uk and are described both in the Michie et al. [1994] book and in the data repository. Their numerical characteristics are given in Table 4 with brief descriptions in the Appendix.

Table 4. Statlog Data Set Summary

Data Set	#Training	#Variables	#Classes	#Test Set
letters	15,000	16	26	5000
satellite	4,435	36	6	2000
shuttle	43,500	9	7	14,500
DNA	2,000	60	3	1186

In each data set, a random 10% of the training set was set aside and a tree grown on the other 90%. The set aside 10% was then used to select the best pruned subtree. In bagging, 50 bootstrap replicates of the training set were generated and a large tree grown on each one. The original training set is used to select the best pruned subtree (see Section 4.3). The test set errors are listed in Table 5.

Table 5. Test Set Misclassification Rates (%)

Data Set	e_S	e_B	Decrease
letters	12.6	6.4	49%
satellite	14.8	10.3	30%
shuttle	.062	.014	77%
DNA	6.2	5.0	19%

Compared to the 22 classifiers in the Statlog Project, bagged trees ranked 2nd in accuracy on the DNA data set, 1st on the shuttle, 2nd on the satellite and 1st on letters. Following the Statlog method of ordering classifiers by their average rank, bagged trees was the top classifier on these four data sets with an average rank of 1.8. The next highest of the 22 has an average rank of 6.3. Average ranks for well-known classifiers are given in Table 6.

Table 6. Average Ranks of Classifiers

Algorithm	Average Rank
Radial Basis Functions	8.0
K-NN	8.5
$C4.5$	9.3
Quad. Discriminant	10.8
Neural Net	12.3

Some of the misclassification rates for the CART algorithm in Table 5 differ from those listed in the Statlog results. Possible sources for these differences are:

i) Different strategies may have been used to grow and prune the tree. I used the 90%-10% method specified above. Its not clear what was done in the Statlog project.

ii) An important setting is the minimum node size. This setting is not specified in the Statlog project. We used a minimum node size of one throughout.

iii) In the DNA data, different preprocessing of the input variable was used (see Appendix).

3. Bagging Regression Trees

Bagging trees was used on five data sets with numerical responses.

Boston Housing	
Ozone	
Friedman #1	(simulated)
Friedman #2	(simulated)
Friedman #3	(simulated)

Table 7. Summary of Data Sets

Data Set	#Cases	# Variables	# Test Set
Boston Housing	506	12	51
Ozone	330	8	33
Friedman #1	200	10	1000
Friedman #2	200	4	1000
Friedman #3	200	4	1000

A summary of these data sets is given in Table 7.

Brief descriptions of the data are in the Appendix. A procedure similar to that used in classification was followed:

i) Each real data set is randomly divided into a test set of consisting of 10% of the data and a learning set $\mathcal{L}$ consisting of the other 90%. In the 3 simulated data sets, 1200 cases are generated, 200 used as learning and 1000 as test.

ii) A regression tree is constructed from $\mathcal{L}$ using 10-fold cross-validation. Running the test set down this tree gives the squared error $e_S(\mathcal{L}, \mathcal{T})$.

iii) A bootstrap sample $\mathcal{L}_B$ is selected from $\mathcal{L}$ and a tree grown using $\mathcal{L}_B$ and $\mathcal{L}$ used to select the pruned subtree. This is repeated 25 times giving tree predictors $\phi_1(\boldsymbol{x}), \ldots, \phi_{25}(\boldsymbol{x})$.

iv) For $(y_n, \boldsymbol{x}_n) \in \mathcal{T}$, the bagged predictor is $\hat{y}_n = av_k\phi_k(\boldsymbol{x}_n)$, and the squared error $e_B(\mathcal{L}, \mathcal{T})$ is $av_n(y_n - \hat{y}_n)^2$

v) The random division of the data into $\mathcal{L}$ and $\mathcal{T}$ is repeated 100 times and the errors averaged to give $\bar{e}_S$, $\bar{e}_B$. For the simulated data, the 1200 cases are newly generated for each repetition.

Table 8 lists the values of $\bar{e}_S$, $\bar{e}_B$, and Table 9 gives their estimated standard errors.

Table 8. Mean Squared Test Set Error

Data Set	$\bar{e}_S$	$\bar{e}_B$	Decrease
Boston Housing	20.0	11.6	42%
Ozone	23.9	18.8	21%
Friedman #1	11.4	6.1	46%
Friedman #2	31,100	22,100	29%
Friedman #3	.0403	.0242	40%

Table 9. Standard Errors

**Data Set**	$SE(\bar{e}_S)$	$SE(\bar{e}_B)$
Boston Housing	1.0	.6
Ozone	.8	.6
Friedman #1	.10	.06
Friedman #2	300	100
Friedman #3	.0005	.0003

4. Why Bagging Works

4.1. Numeric Prediction

Let each $(y, \boldsymbol{x})$ case in $\mathcal{L}$ be independently drawn from the probability distribution P. Suppose y is numerical and $\phi(\boldsymbol{x}, \mathcal{L})$ the predictor. Then the aggregated predictor is the average over $\mathcal{L}$ of $\phi(\boldsymbol{x}, \mathcal{L})$, i.e.

$$\phi_A(\boldsymbol{x}) = E_{\mathcal{L}}\phi(\boldsymbol{x}, \mathcal{L}).$$

Take $\boldsymbol{x}$ to be a fixed input value and y an output value. Then

$$E_{\mathcal{L}}(y - \phi(\boldsymbol{x}, \mathcal{L}))^2 = y^2 - 2yE_{\mathcal{L}}\phi(\boldsymbol{x}, \mathcal{L}) + E_{\mathcal{L}}\phi^2(\boldsymbol{x}, \mathcal{L}). \tag{4.1}$$

Using $E_{\mathcal{L}}\phi(x, \mathcal{L}) = \phi_A(x)$ and applying the inequality $EZ^2 \geq (EZ)^2$ to the third term in (4.1) gives

$$E_{\mathcal{L}}(y - \phi(\boldsymbol{x}, \mathcal{L}))^2 \geq (y - \phi_A(\boldsymbol{x}))^2. \tag{4.2}$$

Integrating both sides of (4.2) over the joint $y, \boldsymbol{x}$ output-input distribution, we get that the mean-squared error of $\phi_A(\boldsymbol{x})$ is lower than the mean-squared error averaged over $\mathcal{L}$ of $\phi(\boldsymbol{x}, \mathcal{L})$.

How much lower depends on how unequal the two sides of

$$[E_{\mathcal{L}}\varphi(\boldsymbol{x}, \mathcal{L})]^2 \leq E_{\mathcal{L}}\varphi^2(\boldsymbol{x}, \mathcal{L})$$

are. The effect of instability is clear. If $\varphi(\boldsymbol{x}, \mathcal{L})$ does not change too much with replicate $\mathcal{L}$ the two sides will be nearly equal, and aggregation will not help. The more highly variable the $\varphi(\boldsymbol{x}, \mathcal{L})$ are, the more improvement aggregation may produce. But φ_A always improves on φ.

Now ϕ_A depends not only on $\boldsymbol{x}$ but also the underlying probability distribution P from which $\mathcal{L}$ is drawn, i.e. $\phi_A = \phi_A(\boldsymbol{x}, P)$. But the bagged estimate is not $\varphi_A(\boldsymbol{x}, P)$, but rather

$$\varphi_B(\boldsymbol{x}) = \varphi_A(\boldsymbol{x}, P_{\mathcal{L}}),$$

where $P_{\mathcal{L}}$ is the distribution that concentrates mass $1/N$ at each point $(y_n, \boldsymbol{x}_n) \in \mathcal{L}$, ($P_{\mathcal{L}}$ is called the bootstrap approximation to P). Then φ_B is caught in two currents: on the one hand, if the procedure is unstable, it can give improvement through aggregation. On the

other side, if the procedure is stable, then $\varphi_B = \varphi_A(\boldsymbol{x}, P_{\mathcal{L}})$ will not be as accurate for data drawn from P as $\varphi_A(\boldsymbol{x}, P) \simeq \varphi(\boldsymbol{x}, \mathcal{L})$.

There is a cross-over point between instability and stability at which φ_B stops improving on $\varphi(\boldsymbol{x}, \mathcal{L})$ and does worse. This has a vivid illustration in the linear regression subset selection example in the next section. There is another obvious limitation of bagging. For some data sets, it may happen that $\varphi(\boldsymbol{x}, \mathcal{L})$ is close to the limits of accuracy attainable on that data. Then no amount of bagging will do much improving. This is also illustrated in the next section.

4.2. Classification

In classification, a predictor $\varphi(\boldsymbol{x}, \mathcal{L})$ predicts a class label $j \in \{1, \ldots, J\}$. Denote

$$Q(j|\boldsymbol{x}) = P(\phi(\boldsymbol{x}, \mathcal{L}) = j).$$

The interpretation of $Q(j|\boldsymbol{x})$ is this: over many independent replicates of the learning set $\mathcal{L}$, ϕ predicts class label j at input $\boldsymbol{x}$ with relative frequency $Q(j|\boldsymbol{x})$. Let $P(j|\boldsymbol{x})$ be the probability that input $\boldsymbol{x}$ generates class j. Then the probability that the predictor classifies the generated state at $\boldsymbol{x}$ correctly is

$$\sum_j Q(j|\boldsymbol{x})P(j|\boldsymbol{x}). \tag{4.3}$$

The overall probability of correct classification is

$$r = \int [\sum_j Q(j|\boldsymbol{x})P(j|\boldsymbol{x})] P_X(d\boldsymbol{x})$$

where $P_X(d\boldsymbol{x})$ is the $\boldsymbol{x}$ probability distribution.

Looking at (4.3) note that for any $Q(j|\boldsymbol{x})$,

$$\sum_j Q(j|\boldsymbol{x})P(j|\boldsymbol{x}) \leq \max_j P(j|\boldsymbol{x})$$

with equality only if

$$Q(j|\boldsymbol{x}) = \begin{cases} 1 & \text{if } P(j|\boldsymbol{x}) = \max_i P(i|\boldsymbol{x}) \\ 0 & \text{else} \end{cases}.$$

The predictor $\phi^*(\boldsymbol{x}) = \text{argmax}_j P(j|\boldsymbol{x})$ (known as the Bayes predictor) leads to the above expression for $Q(j|\boldsymbol{x})$ and gives the highest attainable correct classification rate:

$$r^* = \int \max_j P(j|\boldsymbol{x}) P_X(\boldsymbol{x}).$$

Call ϕ order-correct at the input $\boldsymbol{x}$ if

$$\text{argmax}_j Q(j|\boldsymbol{x}) = \text{argmax}_j P(j|\boldsymbol{x}).$$

This means that if input $\boldsymbol{x}$ results in class j more often than any other class, then ϕ also predicts class j at $\boldsymbol{x}$ more often than any other class. An order-correct predictor is not necessarily an accurate predictor. For instance, in a two class problem, suppose $P(1|\boldsymbol{x}) = .9$, $P(2|\boldsymbol{x}) = .1$ and $Q(1|\boldsymbol{x}) = .6$, $Q(2|\boldsymbol{x}) = .4$. Then the probability of correct classification by ϕ at $\boldsymbol{x}$ is .58, but the Bayes predictor gets correct classification with probability .90.

The aggregated predictor is: $\phi_A(\boldsymbol{x}) = \text{argmax}_j Q(j|\boldsymbol{x})$. For the aggregated predictor the probability of correct classification at $\boldsymbol{x}$ is

$$\sum_j I(\text{argmax}_i Q(i|\boldsymbol{x}) = j) P(j|\boldsymbol{x}) \tag{4.4}$$

where $I(\cdot)$ is the indicator function. If ϕ is order-correct at $\boldsymbol{x}$, then (4.4) equals $\max_j P(j|\boldsymbol{x})$. Letting C be the set of all inputs $\boldsymbol{x}$ at which ϕ is order-correct, we get for the correct classification probability of ϕ_A the expression

$$r_A = \int_{\boldsymbol{x}\in C} \max_j P(j|\boldsymbol{x}) P_X(d\boldsymbol{x}) + \int_{\boldsymbol{x}\in C'} [\sum_j I(\phi_A(\boldsymbol{x}) = j) P(j|\boldsymbol{x})] P_X(\boldsymbol{x}).$$

Even if ϕ is order-correct at $\boldsymbol{x}$ its correct classification rate can be far from optimal. But ϕ_A is optimal. If a predictor is good in the sense that it is order-correct for most inputs $\boldsymbol{x}$, then aggregation can transform it into a nearly optimal predictor. On the other hand, unlike the numerical prediction situation, poor predictors can be transformed into worse ones. The same behavior regarding stability holds. Bagging unstable classifiers usually improves them. Bagging stable classifiers is not a good idea.

4.3. Using the Learning Set as a Test Set

In bagging trees, the training set $\mathcal{L}_B$ is generated by sampling from the distribution $P_{\mathcal{L}}$. Using $\mathcal{L}_B$ a large tree T is constructed. The standard CART methodology finds the sequence of minimum cost pruned subtrees of T. The "best" pruned subtree in this sequence is selected using either cross-validation or a test set.

The idea of a test set is that it is formed by independently sampling from the same underlying distribution that gave rise to the learning set. In the present context, a test set is formed by independently sampling from $P_{\mathcal{L}}$, i.e. we can get a test set by sampling *with replacement*, from the original learning set $\mathcal{L}$.

Consider sampling, with replacement, a large number of times N' from $\mathcal{L}$. If $k(n)$ is the number of times that $(y_n, \boldsymbol{x}_n)$ is selected, then the intuitive content of my argument is that $k(n)/k(n') \simeq 1$, i.e. each case $(y_n, \boldsymbol{x}_n)$ will be selected about the same number of times (ratio-wise) as any other case. Thus, using a very large test set sampled from $P_{\mathcal{L}}$ is equivalent to just using $\mathcal{L}$ as a test set.

A somewhat more convincing argument is this: if there are N cases $(y_n, \boldsymbol{x}_n)$ then the number of times any particular $(y_n, \boldsymbol{x}_n)$ is selected has a binomial distribution with $p = 1/N$, and N' trials. The expected number of times $(y_n, \boldsymbol{x}_n)$ is selected is $N'p = N'/N$. The standard deviation of the number of times $(y_k, \boldsymbol{x}_n)$ is selected is $\sqrt{N'pq} \simeq \sqrt{N'/N}$.

Thus, for all n,

$$k(n)/(N'/N) \simeq 1 + o(1).$$

The fact that $\mathcal{L}$ can be used as a test set for predictors grown on a bootstrap sample $\mathcal{L}_B$ is more generally useful than just in a tree predictor context. For instance, in neural nets early stopping depends on the use of a test set. Thus, in bagging neural nets, the optimal point of early stopping can be estimated using the original learning set as a test set.

5. A Linear Regression Illustration

5.1. Forward Variable Selection

Subset selection in linear regression gives an illustration of the points made in the previous section. With data of the form $\mathcal{L} = \{(y_n, \boldsymbol{x}_n), n = 1, \ldots, N\}$ where $\boldsymbol{x} = (x_1, \ldots, x_M)$ consists of M predictor variables, a popular prediction method consists of forming predictors $\varphi_1(\boldsymbol{x}), \ldots, \varphi_M(\boldsymbol{x})$ where φ_m is linear in $\boldsymbol{x}$ and depends on only m of the M x-variables. Then one of the $\{\varphi_m\}$ is chosen as the designated predictor. For more background, see Breiman and Spector [1992].

A common method for constructing the $\{\varphi_m\}$, and one that is used in our simulation, is forward variable entry. If the variables used in φ_k are $x_{m_1}, \ldots, x_{m_k}$, then for each $m \notin \{m_1, \ldots, m_k\}$ form the linear regression of y on $(x_{m_1}, \ldots, x_{m_k}, x_m)$, compute the residual sum-of-squares RSS(m) and take $x_{m_{k+1}}$ such that m_{k+1} minimizes RSS(m) and $\varphi_{k+1}(\boldsymbol{x})$ the linear regression based on $(x_{m_1}, \ldots, x_{m_{k+1}})$.

There are other forms of variable selection e.g. best subsets, backwards variable selection, and variants thereof. What is clear about all of them is that they are unstable procedures (see Breiman [1994]). The variables are competing for inclusion in the $\{\varphi_m\}$ and small changes in the data can cause large changes in the $\{\varphi_m\}$.

5.2. Simulation Structure

The simulated data used in this section are drawn from the model.

$$y = \sum_m \beta_m x_m + \epsilon$$

where ϵ is $N(0, 1)$ (normally distributed with mean zero and variance one). The number of variables $M = 30$ and the sample size is 60. The $\{x_m\}$ are drawn from a mean-zero joint normal distribution with $EX_iX_j = \rho^{|i-j|}$ and at each iteration, ρ is selected from a uniform distribution on $[0, 1]$.

It is known that subset selection is nearly optimal if there are only a few large non-zero β_m, and that its performance is poor if there are many small but non-zero β_m. To bridge the spectrum, three sets of coefficients are used. Each set of coefficients consists of three clusters; one is centered at $m = 5$, one at $m = 15$ and the other at $m = 25$. Each cluster is of the form

$$\beta_m = c[(h - |m - k|)^+]^2, \quad m = 1, \ldots, 30$$

where k is the cluster center, and $h = 1, 3, 5$ for the first, second and third set of coefficients respectively. Thus, for $h = 1$, there are only three non-zero $\{\beta_m\}$. For $h = 3$ there are 15 non-zero $\{\beta_m\}$, and for $h = 5$, there are 27 non-zero $\{\beta_m\}$, all relatively small. The normalizing constant c is taken so that the R^2 for the data is $\simeq .75$ where R is the correlation between the output variable y and the full least squares predictor.

For each set of coefficients, the following procedure was replicated 250 times:

i) Data $\mathcal{L} = \{(y_n, \boldsymbol{x}_n), n = 1, \ldots, 60\}$ was drawn from the model

$$y = \sum \beta_m x_m + \epsilon$$

where the $\{x_m\}$ were drawn from the joint normal distribution described above.

ii) Forward entry of variables was done using $\mathcal{L}$ to get the predictors $\varphi_1(\boldsymbol{x}), \ldots, \varphi_M(\boldsymbol{x})$. The mean-squared prediction error of each of these was computed giving $e_1, \ldots, e_M$.

iii) Fifty bootstrap replicates $\{\mathcal{L}^{(B)}\}$ of $\mathcal{L}$ were generated. For each of these, forward stepwise regression was applied to construct predictors $\{\varphi_1(\boldsymbol{x}, \mathcal{L}^{(B)}), \ldots, \varphi_M(\boldsymbol{x}, \mathcal{L}^{(B)})\}$. These were averaged over the $\mathcal{L}^{(B)}$ to give the bagged sequence $\varphi_1^{(B)}(\boldsymbol{x}), \ldots, \varphi_M^{(B)}(\boldsymbol{x})$. The prediction errors $e_1^{(B)}, \ldots, e_M^{(B)}$ for this sequence were computed.

These computed mean-squared-errors were averaged over the 250 repetitions to give two sequences $\{\bar{e}_m^{(S)}\}$, $\{\bar{e}_m^{(B)}\}$. For each set of coefficients, these two sequences are plotted vs. m in Figure 1a,b,c.

5.3. Discussion of Simulation Results

Looking at Figures 1a,b,c, an obvious result is that the most accurate bagged predictor is at least as good as the most accurate subset predictor. When $h = 1$ and subset selection is nearly optimal, there is no improvement. For $h = 3$ and 5 there is substantial improvement. This illustrates the obvious: bagging can improve only if the unbagged is not optimal.

The second point is less obvious. Note that in all three graphs there is a point past which the bagged predictors have larger prediction error than the unbagged. The explanation is this: linear regression using *all* variables is a fairly stable procedure. The stability decreases as the number of variables used in the predictor decreases. As noted in Section 4, for a stable procedure $\varphi_B = \varphi_A(\boldsymbol{x}, P_{\mathcal{L}})$ is not as accurate as $\varphi \simeq \varphi(\boldsymbol{x}, P)$. The higher values of $\bar{\varphi}_m^{(B)}$ for large m reflect this fact. As m decreases, the instability increases and there is a cross-over point at which $\varphi_m^{(B)}$ becomes more accurate than φ_m.

6. Concluding Remarks

6.1. Bagging Class Probability Estimates

Some classification methods estimate probabilities $\hat{p}(j|\boldsymbol{x})$ that an object with prediction vector $\boldsymbol{x}$ belongs to class j. Then the class corresponding to $\boldsymbol{x}$ is estimated as $\arg\max_j \hat{p}(j|\boldsymbol{x})$.

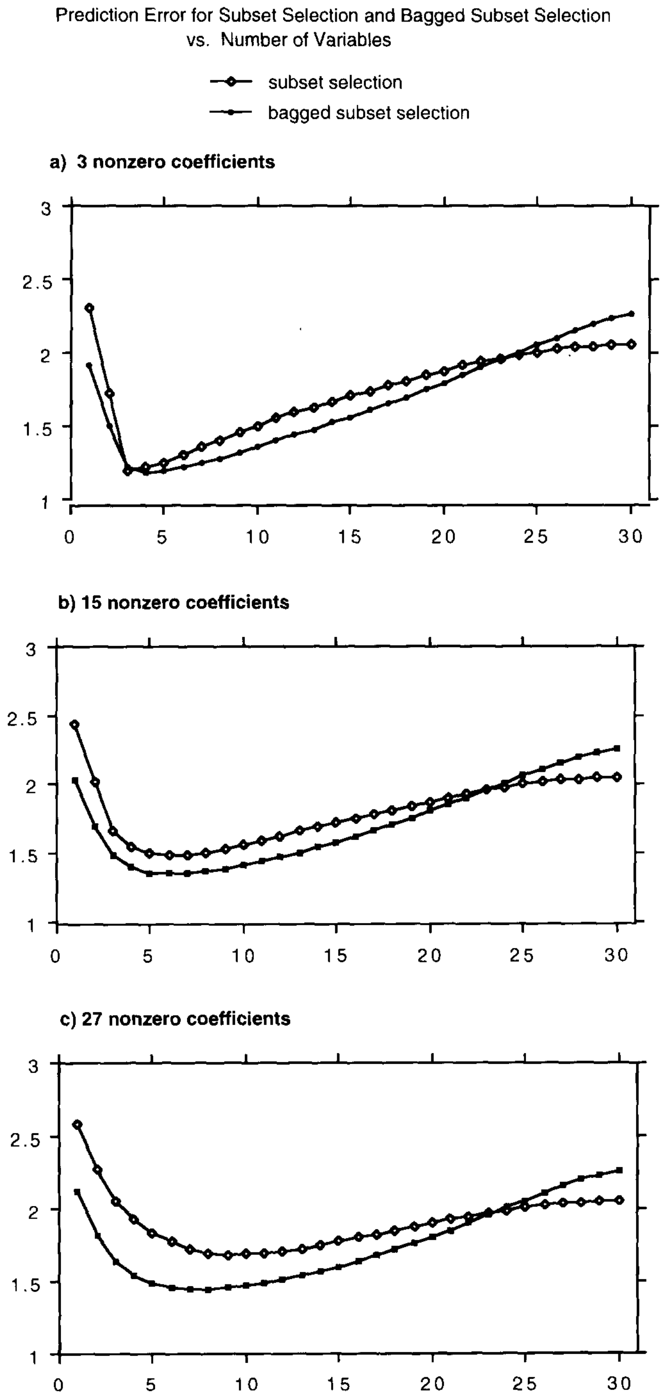

Figure 1. Prediction error for subset selection and bagged subset selection.

For such methods, a natural competitor to bagging by voting is to average the $\hat{p}(j|\boldsymbol{x})$ over all bootstrap replications, getting $\hat{p}_B(j|\boldsymbol{x})$, and then use the estimated class $\arg\max_j \hat{p}_B(j|\boldsymbol{x})$. This estimate was computed in every classification example we worked on. The resulting misclassification rate was always virtually identical to the voting misclassification rate.

In some applications, estimates of class probabilities are required instead of, or along with, the classifications. The evidence so far indicates that bagged estimates are likely to be more accurate than the single estimates. To verify this, it would be necessary to compare both estimates with the true values $p^*(j|\boldsymbol{x})$ over the $\boldsymbol{x}$ in the test set. For real data the true values are unknown. But they can be computed for the simulated waveform data, where they reduce to computing an expression involving error functions.

Using the waveform data, we did a simulation similar to that in Section 2 with learning and test sets both of size 300, and 25 bootstrap replications. In each iteration, we computed the average over the test set and classes of $|\hat{p}(j|\boldsymbol{x}) - p^*(j|\boldsymbol{x})|$ and $|\hat{p}_B(j|\boldsymbol{x}) - p^*(j|\boldsymbol{x})|$. This was repeated 50 times and the results averaged. The single tree estimates had an error of .189. The error of the bagged estimates was .124, a decrease of 34%.

6.2. How Many Bootstrap Replicates Are Enough?

In our experiments, 50 bootstrap replicates was used for classification and 25 for regression. This does not mean that 50 or 25 were necessary or sufficient, but simply that they seemed reasonable. My sense of it is that fewer are required when y is numerical and more are required with an increasing number of classes.

The answer is not too important when procedures like CART are used, because running times, even for a large number of bootstraps, are very nominal. But neural nets progress much more slowly and replications may require many days of computing. Still, bagging is almost a dream procedure for parallel computing. The construction of a predictor on each $\mathcal{L}^{(B)}$ proceeds with no communication necessary from the other CPU's.

To give some ideas of what the results are as connected with the number of bootstrap replicates we ran the waveform data using 10, 25, 50 and 100 replicates using the same simulation scheme as in Section 2. The results appear in Table 10.

Table 10. Bagged Misclassification Rates (%)

No. Bootstrap Replicates	Misclassification Rate
10	21.8
25	19.4
50	19.3
100	19.3

The unbagged rate is 29.1, so its clear that we are getting most of the improvement using only 10 bootstrap replicates. More than 25 bootstrap replicates is love's labor lost.

6.3. *How Big Should the Bootstrap Learning Set Be?*

In all of our runs we used bootstrap replicates $\mathcal{L}^{(B)}$ of the same size as the initial learning set $\mathcal{L}$. While a bootstrap replicate may have $2, 3, \ldots$ duplicates of a given instance, it also leaves out about .37 of the instances. A reader of the technical report on which this paper is based remarked that this was an appreciable loss of data, and that accuracy might improve if a larger bootstrap set was used. We experimented with bootstrap learning sets twice the size of $\mathcal{L}$. These left out about $e^{-2} = .14$ of the instances. There was no improvement in accuracy.

6.4. *Bagging Nearest Neighbor Classifiers*

Nearest neighbor classifiers were run on all the data sets described in Section 2 except for the soybean data whose variables were nominal. The same random division into learning and test sets was used with 100 bootstrap replicates, and 100 iterations in each run. A Euclidean metric was used with each coordinate standardized by dividing by its standard deviation over the learning set. See Table 11 for the results.

Table 11. Misclassification Rates for Nearest Neighbor

**Data Set**	$\bar{e}_S$	$\bar{e}_B$
waveform	26.1	26.1
heart	5.1	5.1
breast cancer	4.4	4.4
ionosphere	36.5	36.5
diabetes	29.3	29.3
glass	30.1	30.1

Nearest neighbor is more accurate than single trees in 3 of the 6 data sets, but bagged trees are more accurate in all of the 6 data sets.

Cycles did not have to be expended to find that bagging nearest neighbors does not change things. Some simple computations show why. Given N possible outcomes of a trial (the N cases $(y_n, \boldsymbol{x}_n)$ in the learning set) and N trials, the probability that the nth outcome is selected $0, 1, 2, \ldots$ times is approximately Poisson distributed with $\lambda = 1$ for large N. The probability that the nth outcome will occur at least once is $1 - (1/e) \simeq .632$.

If there are N_B bootstrap repetitions in a 2-class problem, then a test case may change classification only if its nearest neighbor in the learning set is not in the bootstrap sample in at least half of the N_B replications. This probability is given by the probability that the number of heads in N_B tosses of a coin with probability .632 of heads is less than $.5N_B$. As N_B gets larger, this probability gets very small. Analogous results hold for J-class problems.

The stability of nearest neighbor classification methods with respect to perturbations of the data distinguishes them from competitors such as trees and neural nets.

6.5. Conclusions

Bagging goes a ways toward making a silk purse out of a sow's ear, especially if the sow's ear is twitchy. It is a relatively easy way to improve an existing method, since all that needs adding is a loop in front that selects the bootstrap sample and sends it to the procedure and a back end that does the aggregation. What one loses, with the trees, is a simple and interpretable structure. What one gains is increased accuracy.

Appendix

Descriptions of Data Sets

A. Classification Data Sets

Waveform This is simulated 21 variable data with 300 cases and 3 classes each having probability 1/3. It is described in Breiman et al [1984] (a C subroutine for generating the data is in the UCI repository subdirectory/waveform).

Heart This is data from the study referred to in the opening paragraphs of the CART book (Breiman et. al. [1984]). To quote:

> At the University of California, San Diego Medical Center, when a heart attack patient is admitted, 19 variables are measured during the first 24 hours. These include blood pressure, age, and 17 other ordered and binary variables summarizing the medical symptoms considered as important indicators of the patient's condition.
>
> The goal of a recent medical study (see Chapter 6) was the development of a method to identify high risk patients (those who will not survive at least 30 days) on the basis of the initial 24-hour data.

The data base has also been studied in Olshen et al [1985]. It was gathered on a project (SCOR) headed by John Ross Jr. Elizabeth Gilpin and Richard Olshen were instrumental in my obtaining the data. The data used had 18 variables. Two variables with high proportions of had missing data were deleted, together with a few other cases that missing values. This left 779 complete cases — 77 deaths and 702 survivors. To equalize class sizes, each case of death was replicated 9 times giving 693 deaths for a total of 1395 cases.

Breast Cancer This is data given to the UCI repository by Willian H. Wolberg, University of Wisconsin Hospitals, Madison (see Wolberg and Mangasarian [1990]). It is two class data with 699 cases (458 benign and 241 malignant). It has 9 variables consisting of cellular characteristics.

Ionosphere This is radar data gathered by the Space Physics Group at Johns Hopkins University (see Sigillito et. al. [1989]). There are 351 cases with 34 variables, consisting of 2 attributes for each at 17 pulse numbers. There are two classes: good = some type of structure in the ionosphere (226); bad = no structure (125).

Diabetes This is a data base gathered among the Pima Indians by the National Institute of Diabetes and Digestive and Kidney Diseases. (See Smith et. al. [1988]). The data base consists of 768 cases, 8 variables and two classes. The variables are medical measurements on the patient plus age and pregnancy information. The classes are: tested positive for diabetes (268) or negative (500).

Glass This data base was created in the Central Research Establishment, Home Office Forensic Science Service Aldermaston, Reading, Berkshire. Each case consists of 9 chemical measurements on one of 6 types of glass. There are 214 cases.

Soybean The soybean data set consists of 683 cases, 35 variables and 19 classes. The classes are various types of soybean diseases. The variables are observations on the plants together with some climatic variables. All are nominal. Some missing values were filled in by their modal values.

Letters This data set was constructed by David J. Slate, Odesta Corporation. Binary pixel displays of the 26 capital English letters were created using 20 different fonts and then randomly distorted to create 20,000 images. Sixteen features, consisting of statistical moments and edge counts, were extracted from each image.

Satellite This is data extracted from Landsat images. For the 3×3 pixel area examined, intensity readings are given in 4 spectral bands for each pixel. The middle pixel is classified as one of 6 different soil types. The multiple authorship of the data set is explained in the documentation in the repository.

Shuttle This data set involves shuttle controls concerning the position of radiators within the Space Shuttle. The data originated from NASA and was provided to the archives by J. Catlett.

DNA The classes in this data set are types of boundaries in a spliced DNA sequence. The input variables consists of a window of 60 nucleotides each having one of 4 categorical values (A, G, C, T). The problem is to classify the middle point of the window as one of two types of boundaries or neither. The data is part of the Genbank and was donated by G. Towell, M. Noordewier and J. Shavlik.

Because some classifiers in the Statlog project could accept only numeric inputs, each of the 60 categoricals was coded into 3 binary variables, resulting in 180 input variables. For reasons not explained, some of the tree algorithms run on the DNA data were given the 60 categoricals as input while the CART algorithm was given the 180 binary inputs. In my runs the 60 categorical inputs were used.

B. Regression Data Sets

Boston Housing This data became well-known through its use in the book by Belsley, Kuh, and Welsch [1980]. It has 506 cases corresponding to census tracts in the greater Boston area. The y-variable is median housing price in the tract. There are 12 predictor

variables, mainly socio-economic. The data has since been used in many studies. (UCI repository/housing).

Ozone The ozone data consists of 366 readings of maximum daily ozone at a hot spot in the Los Angeles basin and 9 predictor variables — all meteorlogical, i.e. temperature, humidity, etc. It is described in Breiman and Friedman [1985] and has also been used in many subsequent studies. Eliminating one variable with many missing values and a few other cases leaves a data set with 330 complete cases and 8 variables.

Friedman #1 All three Friedman data sets are simulated data that appear in the MARS paper (Friedman [1991]). In the first data set, there are ten independent predictor variables $x_1, \ldots, x_{10}$ each of which is uniformly distributed over $[0, 1]$. The response is given by

$$y = 10\sin(\pi x_1 x_2) + 20(x_3 - .5)^2 + 10x_4 + 5x_5 + \epsilon$$

where ϵ is $N(0, 1)$. Friedman gives results for this model for sample sizes 50, 100, 200. We use sample size 200.

Friedman #2, #3 These two examples are taken to simulate the impedance and phase shift in an alternating current circuit. They are 4 variable data with

#2 $$y = (x_1^2 + (x_2 x_3 - (1/x_2 x_4))^2)^{1/2} + \epsilon_2$$

#3 $$y = \tan^{-1}\left(\frac{x_2 x_3 - (1/x_2 x_4)}{x_1}\right) + \epsilon_3$$

where x_1, x_2, x_3, x_4 are uniformly distributed over the ranges

$$\begin{aligned} 0 &\le x_1 \le 100 \\ 20 &\le (x_2/2\pi) \le 280 \\ 0 &\le x_3 \le 1 \\ 1 &\le x_4 \le 11 \end{aligned}$$

The noise ϵ_2, ϵ_3 are distributed as $N(0, \sigma_2^2)$, $N(0, \sigma_3^2)$ with σ_2, σ_3 selected to give 3:1 signal/noise ratios.

References

Belsley, D., Kuh, E., & Welsch, R. (1980). "Regression Diagnostics", John Wiley and Sons.

Breiman, L. (1994). Heuristics of instability in model selection, Technical Report, Statistics Department, University of California at Berkeley (to appear, Annals of Statistics).

Breiman, L., Friedman, J., Olshen, R., & Stone, C. (1984). "Classification and Regression Trees", Wadsworth.

Breiman, L. & Friedman, J. (1985). Estimating optimal transformations in multiple regression and correlation (with discussion), *Journal of the American Statistical Association*, **80**, 580-619 .

Breiman, L. & Spector, P (1992). Submodel Selection and Evaluation in Regression – the X-Random Case, *International Statistical Review*, **3**, 291-319

Buntine, W. (1991). "Learning classification trees", Artificial Intelligence Frontiers in Statistics, ed D.J. Hand, Chapman and Hall, London, 182-201.

Dietterich, T.G. & Bakiri, G. (1991). Error-correcting output codes: A general method for improving multiclass inductive learning programs, Proceedings of the Ninth National Conference on Artificial Intelligence (AAAI-91), Anaheim, CA: AAAI Press.

Efron, B., & Tibshirani, R. (1993). "An Introduction to the Bootstrap". Chapman and Hall.

Friedman, J. (1991). Multivariate adaptive regression splines (with discussion), *Annals of Statistics*, **19**, 1-141.

Heath, D., Kasif, S., & Salzberg, S. (1993). k-dt: a multi-tree learning method. Proceedings of the Second International Workshop on Multistrategy Learning, 1002-1007, Chambery, France, Morgan Kaufman.

Kwok, S., & Carter, C. (1990). Multiple decision trees, Uncertainty in Artificial Intelligence 4, ed. Shachter, R., Levitt, T., Kanal, L., and Lemmer, J., North-Holland, 327-335.

Michie, D., Spiegelhalter, D.J. & Taylor, C.C. (1994). Machine Learning, Neural and Statistical Classification. Ellis Horwood Limited.

Olshen, R., Gilpin, A., Henning, H., LeWinter, M., Collins, D., & Ross, J. (1985). Twelve-month prognosis following myocardial infarction: Classification trees, logistic regression, and stepwise linear discrimination, Proceedings of the Berkeley conference in honor of Jerzy Neyman and Jack Kiefer, L. Le Cam;R. Olshen, (Ed), Wadsworth, 245-267.

Smith, J., Everhart, J., Dickson, W., Knowler, W., & Johannes, R. (1988). Using the ADAP learning algorithm to forecast the onset of diabetes mellitus. In Proceedings of the Symposium on Computer Applications and Medical Care 261–265. IEEE Computer Society Press.

Sigillito, V. G., Wing, S. P., Hutton, L. V., & Baker, K. B. (1989). Classification of radar returns from the ionosphere using neural networks. *Johns Hopkins APL Technical Digest*, **10**, 262-266.

Wolberg, W. & Mangasarian, O (1990). Multisurface method of pattern separation for medical diagnosis applied to breast cytology, Proceedings of the National Academy of Sciences, U.S.A., Volume 87, December 1990, pp 9193-9196.

Received September 19, 1994
Accepted January 2, 1995
Final Manuscript November 21, 1995
