# Supplementary material for: A comprehensive simulation study on classification of RNA-Seq data
Source: PLoS One. 2017 Aug 23;12(8):e0182507. doi: 10.1371/journal.pone.0182507 (PMC5568128; doi:10.1371/journal.pone.0182507)
Supplement: S3 File — (ZIP) [file pone.0182507.s003.zip › R Codes/PDFs/SupportVectorMachineEnsemblewithBagging.pdf]

Support Vector Machine Ensemble with Bagging

Hyun-Chul Kim, Shaoning Pang, Hong-Mo Je,
Daijin Kim, and Sung-Yang Bang

Department of Computer Science and Engineering
Pohang University of Science and Technology
San 31, Hyoja-Dong, Nam-Gu, Pohang, 790-784, Korea
{grass,invu71,dkim,sybang}@postech.ac.kr

Abstract. Even the support vector machine (SVM) has been proposed to provide a good generalization performance, the classification result of the practically implemented SVM is often far from the theoretically expected level because their implementations are based on the approximated algorithms due to the high complexity of time and space. To improve the limited classification performance of the real SVM, we propose to use the SVM ensembles with bagging (bootstrap aggregating). Each individual SVM is trained independently using the randomly chosen training samples via a bootstrap technique. Then, they are aggregated into to make a collective decision in several ways such as the majority voting, the LSE(least squares estimation)-based weighting, and the double-layer hierarchical combining. Various simulation results for the IRIS data classification and the hand-written digit recognitionshow that the proposed SVM ensembles with bagging outperforms a single SVM in terms of classification accuracy greatly.

1 Introduction

The support vector machine is a new and promising classification and regression technique proposed by Vapnik and his group at AT&T Bell Laboratories [1]. The SVM learns a separating hyperplane to maximize the margin and to produce a good generalization ability [2]. Recent theoretical research work has solved the existing difficulties of using the SVM in practical applications [3, 4]. By now, it has been successfully applied in many areas, such as the face detection, the hand-writing digital character recognition, and the data mining, etc.

However, the SVM has two drawbacks. First, since it is originally a model for the binary-class classification, we should use a combination of SVMs for the multi-class classification. There are methods for combining binary classifiers for the multi-class classification [6, 7], but when it has been applied to SVM, the performance has not seemed to improve as much as in the binary classification. Second, since learning SVM is time-consuming for a large scale of data, we should use some approximate algorithms [2]. Using the approximate algorithms can reduce the computation time, but degrade the classification performance.

To overcome the above drawbacks, we propose to use the SVM ensembles. We expect that the SVM ensemble can improve the classification performance

S.-W. Lee and A. Verri (Eds.): SVM 2002, LNCS 2388, pp. 397–408, 2002.
© Springer-Verlag Berlin Heidelberg 2002

greatly than using a single SVM by the following fact. Each individual SVM has been trained independently from the randomly chosen training samples and the correctly- classified area in the space of data samples of each SVM becomes limited to a certain area. We can imagine that a combination of several SVMs will expand the correctly-classified area incrementally. This implies the improvement of classification performance by using the SVM ensemble. Likewise, we also expect that the SVM ensemble will improve the classification performance in case of the multi-class classification.

The idea of the SVM ensemble has been proposed in [8]. They used the boosting technique to train each individual SVM and took another SVM for combining several SVMs. In this paper, we propose to use the SVM ensemble based on the bagging technique where each individual SVM is trained over the randomly chosen training samples via a bootstrap technique and the independently trained several SVMs are aggregated in various ways such as the majority voting, the LSE-based weighting, and the double-layer hierarchical combining.

This paper is organized as follows. Section 2 describes the theoretical background of the SVM. Section 3 describes the SVM ensembles, the bagging method and three different combination methods. Section 4 shows the simulation results when the proposed SVM ensemble are applied to the classification problems such as the IRIS data classification and the hand-written digit recognition.Finally, a conclusion is drawn.

2 Support Vector Machines

The classical emprical risk minimization approach, which determines the classification decision function by minimizing the empirical risk as

$$R = \frac{1}{l} \sum_{i=1}^{L} |f(\mathbf{x}_i) - y_i|, \tag{1}$$

where L and f are the size of examples and the classification decision function, respectively. For SVM, determining an optimal separating hyperplane that gives low generalization error is the primary concern. Usually, the classification decision function in the linearly separable problem is represented by

$$f_{w,b} = sign(\mathbf{w} \cdot \mathbf{x} + b). \tag{2}$$

In SVM, the optimal separating hyperplane is determined by giving the largest margin of separation between different classes. This optimal hyperplane bisects the shortest line between the convex hulls of the two classes. The optimal hyperplane is required to satisfy the following constrained minimization as

$$Min : \frac{1}{2}\mathbf{w}^T \mathbf{w},$$

$$y_i(\mathbf{w} \cdot \mathbf{x}_i + b) \geq 1. \tag{3}$$

For the linearly non-separable case, the minimization problem needs to be modified to allow the misclassified data points. This modification results in a soft margin classifier that allows but penalizes errors by introducing a new set of variables $\xi_{i=1}^{l}$ as the measurement of violation of the constraints.

$$Min : \frac{1}{2}\mathbf{w}^T\mathbf{w} + C(\sum_{i=1}^{L}\xi_i)^k,$$

$$y_i(\mathbf{w}^T\varphi(\mathbf{x}_i) + b) \geq 1 - \xi_i, \tag{4}$$

where C and k are used to weight the penalizing variables ξ_i, $\varphi(\cdot)$ is a nonlinear function which maps the input space into a higher dimensional space. Minimizing the first term in Eq.(4) is corresponding to minimizing the VC-dimension of the learning machine and minimizing the second term in Eq.(4) controls the empirical risk. Therefore, in order to solve problem Eq.(4), we need to construct a set of functions, and implement the classical risk minimization on the set of functions. Here, a Lagrangian method is used to solve the above problem. Then, Eq.(4) can be written as

$$Max : F(\wedge) = \wedge \cdot 1 - \frac{1}{2} \wedge \cdot D\wedge,$$

$$\wedge \cdot y = 0; \wedge \leq C; \wedge \geq 0, \tag{5}$$

where $\wedge = (\lambda_1, ..., \lambda_L)$,$D = y_i y_j x_i \cdot x_j$. For the binary classification, the decision function Eq.(2) can be rewritten as

$$f(\mathbf{x}) = sign(\sum_{i=1}^{l} y_i \lambda_i^*(\mathbf{x}) + b^*)). \tag{6}$$

where $\lambda_i^*(\mathbf{x}) = \lambda_i y_i K(\mathbf{x}, \mathbf{x}_i)$, and $K(\mathbf{x}, \mathbf{x}_i) = \phi(\mathbf{x})\phi(\mathbf{x}_i)$. ($K(\mathbf{x}, \mathbf{x}_i)$ can be simplifed by the kernel trick [9].)

For the multi-class classification, we can extend the SVM in the following two ways. One method is called the "one-against-all" method [6], where we have as many SVMs as the number of classes. The ith SVM is trained from the training samples where some examples contained in the ith class have "+1" labels, and other examples contained in the other classes have "-1" labels. Then, Eq.(4) is modified into

$$\begin{aligned} Min : F(\wedge) = \frac{1}{2}(\mathbf{w}_i)^T\mathbf{w}_i + C(\sum_{t=1}^{L}(\xi^i)_t)^k \\ y_i((\mathbf{w}_i)^T\varphi(\mathbf{x}_t) + b_i) \geq 1 - (\xi^i)_t,\ if\ \mathbf{x}_i \in C_i, \\ y_i((\mathbf{w}_i)^T\varphi(\mathbf{x}_t) + b_i) \leq 1 - (\xi^i)_t,\ if\ \mathbf{x}_i \in \bar{C}_i, \\ (\xi^i)_t > 0,\ i = 1, ..., L, \end{aligned} \tag{7}$$

where C_i is the set of data of class i. The decision function of (7) becomes

$$f(\mathbf{x}) = sign(\underset{j\in 1,2,\ldots,C}{Max}((\mathbf{w}^j)^T \cdot \varphi(\mathbf{x}_j) + b_j)), \tag{8}$$

where C is the number of the classes.

Another method is called the one-against-one method [7]. When the number of classes is C, this method constructs $C(C-1)/2$ SVM classifiers. The ijth SVM is trained from the training samples where some examples contained in the ith class have "+1" labels and other examples contained in the jth class have "-1" labels. Then, Eq.(4) is modified into

$$\begin{aligned} Min: F(\wedge) = \frac{1}{2}(\mathbf{w}_{ij})^T\mathbf{w}_{ij} + C(\sum_{t=1}^{L}(\xi^{ij})_t)^k \\ y_t((\mathbf{w}_{ij})^T\varphi(\mathbf{x}_t) + b_{ij}) \geq 1 - (\xi^{ij})_t,\ if\ x_t \in C_i, \\ y_t((\mathbf{w}_{ij})^T\varphi(\mathbf{x}_t) + b_{ij}) \leq 1 - (\xi^{ij})_t,\ if\ x_t \in C_j, \\ (\xi^{ij})_t > 0, t = 1, ..., L. \end{aligned} \tag{9}$$

The class decision in this type of multi-class classifier can be performed in the following two ways. The first decision is based on the "Max Wins" voting strategy, in which $C(C-1)/2$ binary SVM classifiers will vote for each class, and the winner class having the maximum votes is the last classification decision. The second method uses the tournament match, which reduces the classification time to the log scale.

3 Support Vector Machine Ensemble

An ensemble of classifiers is a collection of several classifiers whose individual decisions are combined in some way to classify the test examples [10]. It is known that an ensemble often shows much better performance than the individual classifiers that make it up. Hansen et. al. [11] shows why the ensemble shows better performance than individual classifiers as follows. Assume that there are an ensemble of n classifiers: $\{f_1, f_2, \ldots, f_n\}$ and consider a test data $\mathbf{x}$. If all the classifiers are identical, they are wrong at the same data, where an ensemble will show the same performance as individual classifiers. However, if classifiers are different and their errors are uncorrelated, then when $f_i(\mathbf{x})$ is wrong, most of other classifiers except for $f_i(\mathbf{x})$ may be correct. Then, the result of majority voting can be correct. More precisely, if the error of individual classifier is $p < 1/2$ and the errors are independent, then the probability p_E that the result of majority voting is incorrect is $\sum_{k=\lceil n/2 \rceil}^{n} p^k(1-p)^{(n-k)}$ $(< \sum_{k=\lceil n/2 \rceil}^{n} (\frac{1}{2})^k(\frac{1}{2})^{(n-k)} = \sum_{k=\lceil n/2 \rceil}^{n} (\frac{1}{2})^n)$. When the size of classifiers n is large, the probability p_E becomes very small.

The SVM has been known to show a good generalization performance and is easy to learn exact parameters for the global optimum[2]. Because of these

advantages, their ensemble may not be considered as a method for improving the classification performance greatly. However, since the practical SVM has been implemented using the approximated algorithms in order to reduce the computation complexity of time and space, a single SVM may not learn exact parameters for the global optimum. Sometimes, the support vectors obtained from the learning is not sufficient to classify all unknown test examples completely. So, we can not guarantee that a single SVM always provides the global optimal classification performance over all test examples.

To overcome this limitation, we propose to use an ensemble of support vector machines. Similar arguments mentioned above about the general ensemble of classifiers can also be applied to the ensemble of support vector machines. Figure 1 shows a general architecture of the proposed SVM ensemble. During the training phase, each individual SVM is trained independently by its own replicated training data set via a bootstrap method explained in the Section 3.1. All constituent SVMs will be aggregated by various combination strategies explained in the Section 3.2. During the testing phase, a test example is applied to all SVMs simultaneously and a collective decision is obtained based on the aggregation strategy.

On the other hand, the advantage of using the SVM ensemble over a single SVM can be achieved equally in the case of multi-class classification. Since the SVM is originally a binary classifier, many SVMs should be combined for the multi-class classification as mentioned in 2.2. The SVM classifier for the multi-

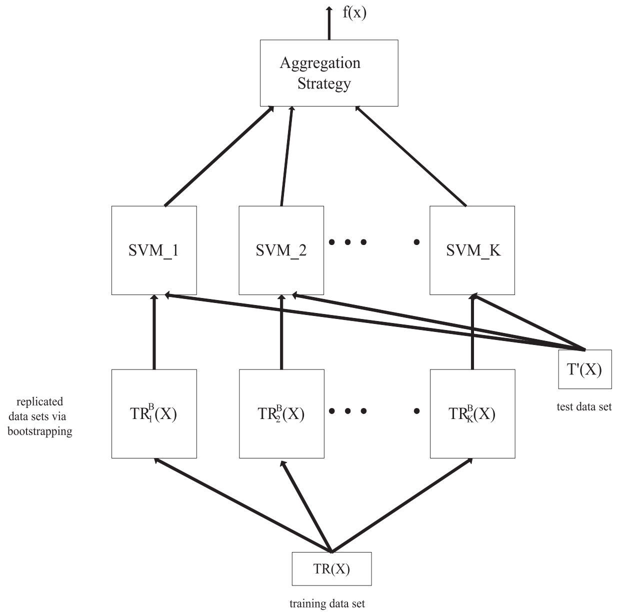

Fig. 1. A general architecture of the SVM ensemble

class classification does not show as good performance as that for the binary-class classification. So, we can also improve the classification performance in the multi-class classification by taking the SVM ensemble where each SVM classifier is designed for the multi-class classification.

3.1 Constructing the SVM Ensembles Using Bagging

In this work, we adopt a bagging technique [12] to construct the SVM ensemble. In bagging, several SVMs are trained independently via a bootstrap method and then they are aggregated via an appropriate combination technique.

Usually, we have a single training set Usually, we have a single training set $TR = \{(\mathbf{x}_i; y_i) | i = 1, 2, \ldots, l\}$. But we need K training samples sets to construct the SVM ensemble with K independent SVMs. From the statistical fact, we need to make the training sample sets different as much as possible in order to obtain higher improvement of the aggregation result. For doing this, we often use the bootstrap technique as follows.

Bootstrapping builds K replicate training data sets $\{TR_k^B | k = 1, 2, \ldots, K\}$ by randomly resampling, but with replacement, from the given training data set TR repeatedly. Each example $\mathbf{x}_i$ in the given training set TR may appear repeated times or not at all in any particular replicate training data set. Each replicate training set will be used to train a certain SVM.

3.2 Aggregating Support Vector Machines

After training, we need to aggregate several independently trained SVMs in an appropriate combination manner. We consider two types of combination techniques such as the linear and nonlinear combination method. The linear combination method, as a linear combination of several SVMs, includes the majority voting and the LSE-based weighting. The majority voting and the LSE-based weighting are often used for the bagging and the boosting, respectively. A nonlinear method, as a nonlinear combination of several SVMs, includes the double-layer hierarchical combining that use another upper-layer SVM to combine several lower-layer SVMs.

Majority Voting Majority voting is the simplest method for combining several SVMs. Let $f_k (k = 1, 2, \ldots, K)$ be a decision function of the kth SVM in the SVM ensemble and $C_j (j = 1, 2, \ldots, C)$ denote a label of the j-th class. Then, let $N_j = \#\{k | f_k(\mathbf{x}) = C_j\}$, i.e. the number of SVMs whose decisions are known to the jth class. Then, the final decision of the SVM ensemble $f_{mv}(\mathbf{x})$ for a given test vector $\mathbf{x}$ due to the majority voting is determined by

$$f_{mv}(\mathbf{x}) = \underset{j}{argmax} N_j. \tag{10}$$

The LSE-Based Weighting The LSE-based weighting treats several SVMs in the SVM ensemble with different weights. Often, the weights of several SVMs are determined in proportional to their accuracies of classifications [13]. Here, we propose to learn the weights using the LSE method as follows.

Let $f_k(k = 1, 2, \ldots, K)$ be a decision function of the kth SVM in the SVM ensemble that is trained by a replicate training data set $Thau_k^B = \{(\mathbf{x}_i'; y_i')|i = 1, 2, \ldots, L\}$. The weight vector $\mathbf{w}$ can be obtained by $\mathbf{w}_E = \mathbf{A}^{-1}\mathbf{y}$, where $\mathbf{A} = (f_i(\mathbf{x}_j))_{K \times L}$, and $\mathbf{y} = (y_j)_{1 \times L}$. Then, the final decision of the SVM ensemble $f_{mv}(\mathbf{x})$ for a given test vector $\mathbf{x}$ due to the LSE-based weighting is determined by

$$f_{LSE}(\mathbf{x}) = sign(\mathbf{w} \cdot [(f_i(\mathbf{x}))_{K \times 1}]) \tag{11}$$

The Double-Layer Hierarchical Combining We can use another SVM to aggregate the outputs of several SVMs in the SVM ensemble. So, this combination consists of a double-layer of SVMs hierarchically where the outputs of several SVMs in the lower layer feed into a super SVM in the upper layer. This type of combination looks similar of the mixture of experts introduced by M. Jordan et. al. [14].

Let $f_k(k = 1, 2, \ldots, K)$ be a decision function of the kth SVM in the SVM ensemble and F be a decision function of the super SVM in the upper layer. Then, the final decision of the SVM ensemble $f_{SVM}(\mathbf{x})$ for a given test vector $\mathbf{x}$ due to the double-layer hierarchical combining is determined by

$$f_{SVM}(\mathbf{x}) = F((f_1(\mathbf{x}), f_2(\mathbf{x}), \ldots, f_K(\mathbf{x}))), \tag{12}$$

where K is the number of SVMs in the SVM ensemble.

3.3 Extension of the SVM Ensemlbe to the Multi-class Classification

In section 2, we explained two kinds of extension methods such as "one-against-all and one-against-one methods" in order to apply the SVM to the multi-class classification problem. We can use these extension methods equally in the case of the SVM ensemble for the multi-class classification. For the C-class classification problem, we can have two types of extensions according to the insertion level of the SVM ensemble: (1) the binary-classifier-level SVM ensemble and (2) the multi-classifier-level SVM ensemble.

The binary-classifier-level SVM ensemble consists of C SVM ensembles in the case of "one-against-all" method or $C(C-1)/2$ SVM ensembles in the case of "one-against-one" method. And, each SVM ensemble consists of K independent SVMs. So, the SVM ensemble is built in the level of binary classifiers. We obtain the final decision from the decision results of many SVM ensembles via either the "Max Wins" voting strategy or the tournament match.

The multi-classifier-level SVM ensemble consists of K multi-class classifiers. And each multi-class classifier consists of C binary classifiers in the case of

"one-against-all" method or for $C(C-1)/2$ binary classifiers in the case of "one-against-one" method. So, the SVM ensemble is built in the level of multi-class classifiers. We obtain the final decision from the decision results of many multi-class classifiers via an appropriate aggregating strategy of the SVM ensemble.

4 Simulation Results

To evaluate the efficacy of the proposed SVM ensemble using the bagging technique, we have performed three different classification problems such as the IRIS data classification, the UCI hand-written digit recognition.We used four different classification methods such as a single SVM, and three different SVM ensembles with different aggregating strategies like the majority voting, the LSE-based weighting, and the double-layer hierarchical combining.

4.1 IRIS Data Classification

The IRIS data set[15, 16] is one of the best known databases to be found in the pattern recognition literature. The IRIS data set contains 3 classes where each class consists of 50 instances. Each class refers to a type of IRIS planet. One class is linearly separable from the other classes but they are not linearly separable from each other.

We used the one-against-one method for the multi-class extension and took the binary-classifier-level SVM ensemble for the classification. So, for the 3-class classification problem and one-against-one extension method, there are three binary-classifier-level SVM ensembles $(SVM_{C1,C1}, SVM_{C1,C2}, SVM_{C1,C3})$ where each SVM ensemble consists of 5 independent SVMs. The final decision are obtained from the decision results of three SVM ensembles via a tournament matching scheme. Each SVM used 2-d polynomial kernel function.

We selected randomly 90 data samples for the training set. For bootstrapping, we re-sampled randomly 60 data samples with replacement from the training data set. We trained each SVM independently over the replicated training data set and aggregated several trained SVMs via three different combination methods. We test four different classification methods using the test data set consisting of 60 IRIS data. Table 1 shows the classification results of four different classification methods for the IRIS data classification.

Table 1. The classification results of IRIS data classification

Method	Classification rate
Single SVM	96.73%
Majoirity voting	98.0%
LSE-based weighting	98.66%
Hierarchical SVM	98.66%

4.2 UCI Hand-Written Digit Recognition

We used the UCI hand-written digit data [16]. Some digits in the database are shown in Figure 2. Among the UCI hand-written digits, we chose randomly 3,828 digits as a training data set and the remaining 1,797 digits as a test data set. The original image of each digit has the size of 32×32 pixels. It is reduced to the size of 8×8 pixels where each pixel is obtained from the average of the block of 4×4 pixels in the original image. So each digit is represented by a feature vector with the size of 64×1.

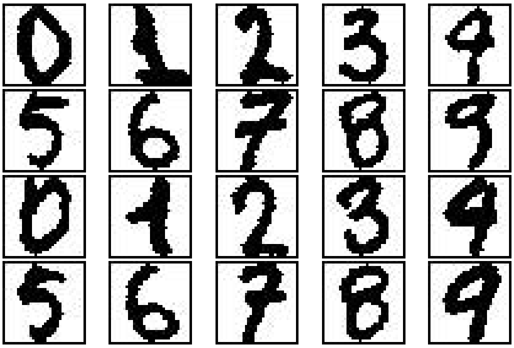

Fig. 2. Examples of hand-written digits in UCI database

We used one-against-one methods. We constructed a SVM ensemble, consiting of 11 SVMs, for one-against-one classification, and used the tournament scheme for decision. Each SVM used 2-d polynomial kernel function. For bagging, we sampled 2000 data randomly for an individual SVM. We trained each SVM and combined three methods, such as unweighted voting, weighted voting (using LSE), and a combining SVM. We applied a single SVM for the comparison. Table 2 shows the performance of a single SVM and bagging SVMs with three combining methods for the IRIS data classification.

We used the one-against-one method for the multi-class extension and took the binary-classifier-level SVM ensemble for the classification. So, for the 10-class classification problem and one-against-one extension method, there are 45 binary-classifier-level SVM ensembles $(SVM_{C0,C1}, SVM_{C0,C2}, \ldots, SVM_{C8,C9})$ where each SVM ensemble consists of 11 independent SVMs. The final decision are obtained from the decision results of three SVM ensembles via a tournament matching scheme. Each SVM used 2-d polynomial kernel function.

For bootstrapping, we re-sampled randomly 2,000 digits samples with replacement from the training data set consisting of 3,828 digit samples. We trained each SVM independently over the replicated training data set and aggregated several trained SVMs via three different combination methods. We test four different classification methods using the test data set consisting of 1,797 digits.

Table 2 shows the classification results of four different classification methods for the hand-written digit data classification.

Table 2. The classification results of UCI hand-written digit recognition

Method	Classification rate
Single SVM	96.99%
Majority voting	97.55%
LSE-based weighting	97.82%
Hierarchical SVM	98.01%

5 Conclusion

Usually, the practical SVM has been implemented based on the approximation algorithm to reduce the cost of time and space. So, the obtained classification performance is far from the theoretically expected level of it. To overcome this limitation, we addressed the SVM ensemble that consists of several independently trained SVMs. For training each SVM, we generated many replicated training sample sets via the bootstrapping technique. Then, all independently trained SVMs over the replicated training sample sets were aggregated by three combination techniques such as the majority voting, the LSE-based weighting, and the double-layer hierarchical combining.

We also extended the SVM ensemble to the multi-class classification problem: the binary-classifier-level SVM ensemble and the multi-classifier-level SVM ensemble. The former did build the SVM ensemble in the level of binary classifiers and the latter did build the SVM ensemble in the level of multi-class classifiers. The former had C SVM ensembles in the case of "one-against-all" method or $C(C-1)/2$ SVM ensembles in the case of "one-against-one" method. And, each SVM ensemble consisted of K independent SVMs. The latter consisted of K multi-class classifiers. And each multi-class classifier consists of C binary classifiers in the case of "one-against-all" method or for $C(C-1)/2$ binary classifiers in the case of "one-against-one" method.

We evaluated the classification performance of the proposed SVM ensemble over three different multi-class classification problems such as the IRIS data classification, the hand-written digit recognition. The SVM ensembles outperform a single SVM for all applications in terms of classification accuracy. For three different aggregation methods, the classification performance is superior in the order of the double-layer hierarchical combining, the LSE-based weighting, and the majority voting. In the future, we will consider other aggregation scheme like boosting for constructing the SVM ensemble.

Acknowledgements

The authors would like to thank the Ministry of Education of Korea for its financial support toward the Electrical and Computer Engineering Division at POSTECH through its BK21 program. This research was also partially supported by the Brain Science and Engineering Research Program of the Ministry of Science and Technology, Korea, and by the Basic Research Institute Support Program of the Korea Research Foundation.

References

[1] Cortes, C., Vapnik, V.: Support vector network. Machine Learning. **20** (1995) 273–297 397
[2] Burges, C.: A Tutorial on Support Vector Machines for Pattern Recognition. Data Mining and Knowledge Discovery. **2**(2) (1998) 121–167 397, 400
[3] Joachims, T.: Making large-scale support vector machine learning practical. Advances in Kernel Methods: Support Vector Machines, MIT Press, Cambridge, MA (1999) 397
[4] Platt, J.: Fast training of support vector machines using sequential minimal optimization. Advances in Kernel Methods: Support Vector Machines, MIT Press, Cambridge, MA (1999) 397
[5] Weston, J., Watkins, C.: Support Vector Machines for Multi-Class Pattern Recognition. Proceedings of the 7th European Symposium on Artificial Neural Networks (1999)
[6] Bottou, L., Cortes, C., Denker, J., Drucker, H., Guyon, I., Jackel, Lawrence D., LeCun, Y., Müller U., Säckinger E., Simard, P., Vapnik, V.: Comparison of classifier methods: a case study in handwriting digit recognition. Proceedings of the 13th International Conference on Pattern Recognition. IEEE Computer Society Press (1994) 77–87 397, 399
[7] Knerrm, S., Personnaz, L., Dreyfus, G.: Single-layer learning revisited: a stepwise procedure for building and training a neural network. In: Fogelman, J.(eds.): Neurocomputing: Algorithms, Architechtures and Application, Springer-Verlag (1990) 397, 400
[8] Vapnik, V.: The Nature of Statistical Learning Theory. Springer, New York, 1999 398
[9] Schölkopf, B., Smola A., and Muller K.: Nonlinear component analysis as a kernel eigenvalue problem. Neural Computation, **10**(5) (1998) 1299-1319 399
[10] Dietterich, T.: Machine Learning Research: Four Current Directions. The AI Magazine, **18**(4) (1998) 97–136 400
[11] Hansen, L., Salamon, P.: Neural network ensembles. IEEE Transactions on Pattern Analysis and Machine Intelligence, **12** (1990) 993–1001 400
[12] Breiman, L.: Bagging predictors. Machine Learning, **24**(2) (1996) 123–140, 1996 402
[13] Kim, D., Kim, C.: Forecasting time series with genetic fuzzy predictor ensemble. IEEE Transaction on Fuzzy Systems, **5**(4) (1997) 523–535 403
[14] Jordan, M., Jacobs, R., Hierarchical mixtures of experts and the EM algorithm. Neural Computation **6**(5) (1994) 181–214 403
[15] Fisher, R.: The use of multiple measurements in taxonomic problems. Annual Eugenics, 7, Part II (1936) 179–188 404

[16] Bay, B.: The UCI KDD Archive [http://kdd.ics.uci.edu]. Irvine, CA: University of California, Department of Information and Computer Science (1999) 404, 405
