# Supplementary material for: A comprehensive simulation study on classification of RNA-Seq data
Source: PLoS One. 2017 Aug 23;12(8):e0182507. doi: 10.1371/journal.pone.0182507 (PMC5568128; doi:10.1371/journal.pone.0182507)
Supplement: S4 File — (DOCX) [file pone.0182507.s004.docx]

Table 1. Computational properties of the used workstations in analysis

| **Workstation** | **Operating  system** | **CPU** | **Memory** | **Number  of cores** |
| --- | --- | --- | --- | --- |
| Hacettepe University,  Department of Biostatistics | Windows 7 | Core i7 3960X, 3.30 GHz | 64 GB | 12 |
| Personal Computer | Ubuntu 14.04 LTS | Core i7 4770,  3.40 GHz | 16 GB | 8 |
| Erciyes University,  Department of Biostatistics | OS X Yosemite 10.10.2 | Core i7 Quad Core, 4 GHz | 32 GB | 8 |
| Erciyes University,  Genome and Stem Cell Center,  Division of Bioinformatics | Windows 8 | Xeon E5-1650, 3.20 GHz | 64 GB | 12 |
| Erciyes University,  Genome and Stem Cell Center,  Division of Bioinformatics | Ubuntu 14.10 | Xeon E5-1650, 3.20 GHz | 16 GB | 12 |
| Marmara University,  Department of Physics | Windows 7 | Core i7 3930K, 3.20 GHz | 16 GB | 8 |
| University of California, San Diego Supercomputer Center | OS X Yosemite 10.10.2 | Xeon Quad Core, 2x2.66 GHz | 16 GB | 8 |
