# Supplementary material for: A comprehensive simulation study on classification of RNA-Seq data
Source: PLoS One. 2017 Aug 23;12(8):e0182507. doi: 10.1371/journal.pone.0182507 (PMC5568128; doi:10.1371/journal.pone.0182507)
Supplement: S5 File — (DOCX) [file pone.0182507.s005.docx]

**Table 1. Computational costs of classifiers for real datasets**

| **Classifier** | **Cervical** | **Alzheimer** | **Renal Cell Cancer** | **Lung Cancer** |
| --- | --- | --- | --- | --- |
| **NBLDA** | 0.63 | 0.29 | 0.94 | 0.60 |
| **PLDA1** | 1.23 | 0.76 | 20.76 | 16.70 |
| **PLDA2** | 1.49 | 0.96 | 29.17 | 21.65 |
| **CART** | 0.34 | 0.12 | 0.49 | 0.34 |
| **RF** | 1.41 | 0.88 | 116.82 | 94.20 |
| **SVM** | 2.14 | 1.06 | 7.02 | 5.63 |
| **bagSVM** | 122.46 | 68.33 | 398.55 | 301.01 |

Values are given in seconds.
